# Parameterized model checking of rendezvous systems

**DOI:** 10.1007/s00446-017-0302-6

**Published:** 2017-06-06

**Authors:** Benjamin Aminof, Tomer Kotek, Sasha Rubin, Francesco Spegni, Helmut Veith

**Affiliations:** 1 Technische Universität Wien, Vienna, Austria; 2 Università degli Studi di Napoli “Federico II”, Naples, Italy; 3 Università Politecnica delle Marche, Ancona, Italy

## Abstract

Parameterized model checking is the problem of deciding if a given formula holds irrespective of the number of participating processes. A standard approach for solving the parameterized model checking problem is to reduce it to model checking finitely many finite-state systems. This work considers the theoretical power and limitations of this technique. We focus on concurrent systems in which processes communicate via pairwise rendezvous, as well as the special cases of disjunctive guards and token passing; specifications are expressed in indexed temporal logic without the next operator; and the underlying network topologies are generated by suitable formulas and graph operations. First, we settle the exact computational complexity of the parameterized model checking problem for some of our concurrent systems, and establish new decidability results for others. Second, we consider the cases where model checking the parameterized system can be reduced to model checking some fixed number of processes, the number is known as a cutoff. We provide many cases for when such cutoffs can be computed, establish lower bounds on the size of such cutoffs, and identify cases where no cutoff exists. Third, we consider cases for which the parameterized system is equivalent to a single finite-state system (more precisely a Büchi word automaton), and establish tight bounds on the sizes of such automata.

## Introduction

Many concurrent systems consist of an arbitrary number of identical processes running in parallel, possibly in the presence of an environment or control process. The parameterized model checking problem (PMCP) for concurrent systems is to decide if a given temporal logic specification holds irrespective of the number of participating processes.

Although the PMCP is undecidable in general (see [28, 52]) it becomes decidable for some combinations of communication primitives, network topologies, and specification languages, e.g., [1, 8, 14, 21, 22, 30, 51]. Often, it is proved decidable by a reduction to model checking finitely many finite-state systems [2, 16, 24, 28, 36]. In many of these cases it is even possible to reduce the problem of whether a parameterized system satisfies a temporal specification for any number of processes to the same problem for systems with at most *c* processes. In fact, it is usually of interest to find such a number *c* that works for every specification formula of a given temporal logic [2, 10, 16, 26, 28]. Such a number is known as a cutoff for the given parameterized system.^1^ In other cases the reduction produces a single finite-state system, often in the form of a fair transition system (such as a Büchi automaton), that represents the set of all execution traces of systems of all sizes. Note that PMCP is at least as hard as ordinary model checking since one can consider a replicated process that does not communicate with any others.

The goal of this paper is to better understand the power and limitations of these techniques, and this is guided by three concrete questions.

*Question 1*. For which combinations of communication primitives, specification languages, and network topologies is the PMCP decidable? In case of a decidable configuration, what is the computational complexity of the PMCP?

In case a cutoff *c* exists, the PMCP is decidable by a reduction to model checking *c* many finite-state systems. The complexity of this procedure depends on the size of the cutoff. Thus we ask:

*Question 2*. When do cutoffs exist? In case a cutoff exists, can one compute a cutoff? And if so, is the computed cutoff the smallest possible?

The set of execution traces of a parameterized system (for a given process type *P*) is defined as the projection onto the local states of *P* of all (infinite) runs of systems of all sizes.^2^ In case this set is $\omega$-regular, one can reduce the PMCP of certain specifications (including classic ones such as coverability) to the language containment problem for automata (this is the approach taken in [36, Section 4]). Thus we ask:

*Question 3*. Is the set of executions of the system $\omega$-regular? And if so, how big is a non-deterministic Büchi word automaton recognizing this set?

**System model**

In order to model and verify a concurrent system we should specify three items: (i) the communication primitive, (ii) the specification language, and (iii) the set of topologies.

(i) In this work we focus on concurrent systems in which processes have finitely many states and communicate via pairwise rendezvous [36]. Pairwise-rendezvous is like CSP message passing, and can model, for instance, the communication in population protocols [9, 32], vector-addition systems with states (or equivalently, Petri nets) [29], and concurrent Boolean programs with locks or shared variables [12, 42]. We also treat two other communication primitives which are expressible in terms of pairwise rendezvous, namely disjunctive guards [24] and token-passing systems [2, 16, 28]. A much more powerful communication is broadcast [27, 31], which is like Ethernet broadcast and the notifyAll method in Concurrent Java [20]. The relative expressive power of pairwise-rendezvous and various other communication primitives, including disjunctive guards and broadcast, is studied in [7], and decidability of the PMCP with all the mentioned communication primitives, as well as others, is summarised in [14].
(ii) Specifications of parameterized systems are typically expressed in indexed temporal logic [15] which allows one to quantify over processes. For instance, the formula $\forall i \ne j.\; \textsf {A}\textsf {G}(\lnot \text{(critical }, i) \vee \lnot \text{(critical }, j))$ says that no two processes are in their critical sections at the same time. We focus on a fragment of this logic where the process quantifiers only appear at the front of a temporal logic formula—allowing the process quantifiers to appear in the scope of path quantifiers results in undecidability even with no communication between processes [41].
(iii) The sets of topologies we consider all have either bounded tree-width, or more generally bounded clique-width, and are expressible in one of three ways.^3^

1. Using $\textsf {MSO}$, a powerful and general formalism for describing sets of topologies, which can express e.g., planarity, acyclicity and $\ell$-connectivity.
2. As *iteratively constructible* sets of topologies, an intuitive formalism which creates graph sequences by iterating graph operations [34]; many typical classes of topologies (e.g., all rings, all stars, all cliques) are iteratively constructible.
3. As *homogeneous* sets of topologies, which includes, e.g., the set of cliques and the set of stars, but excludes the set of rings.

Iteratively constructible and homogeneous sets of topologies are $\textsf {MSO}$-definable, the former in the presence of certain auxiliary relations.

**Prior work and our contributions**

For each communication primitive (rendezvous, disjunctive guards, token passing) and each question (decidability and complexity, cutoffs, equivalent automata) we summarise the known answers and our contributions. Obviously, the breadth of questions along these axes is great, and we had to limit our choices as to what to address. Thus, this article is not meant to be a comprehensive taxonomy of PMCP. That is, it is not a mapping of the imaginary hypercube representing all possible choices along these axes. Instead, we started from the points in this hypercube that represent the most prominent known results and, guided by the three main questions mentioned earlier, have explored the unknown areas in each point’s neighborhood.

**Pairwise rendezvous**

*Decidability and complexity*. The PMCP for systems communicating by pairwise rendezvous, on clique topologies, with a controller *C*,^4^ for 1-index $\textsf {LTL}\backslash \textsf {X}$ specifications is Expspace-complete [30, 36] (Pspace-complete without a controller [36, Section 4]). We show the PMCP is undecidable if we allow the more general 1-index $\textsf {CTL}^*\backslash \textsf {X}$ specifications. Thus, for the results on pairwise rendezvous we fix the specification language to be 1-index $\textsf {LTL}\backslash \textsf {X}$. We prove that the PMCP of 1-index $\textsf {LTL}\backslash \textsf {X}$ remains in Expspace even if one allows homogeneous topologies (Pspace-complete without a controller). We also prove that the program complexity is in Expspace (Ptime without a controller). In contrast, if one allows non-homogeneous topologies, the PMCP is much harder, e.g., it is undecidable for the simple case of unidirectional rings and 1-index safety specifications (this is implied by [28, 52]).

*Cutoffs*. We show that even for clique topologies there are not always cutoffs.

*Equivalent automata*. We prove that the set of executions of systems with a controller are not, in general, $\omega$-regular, already for clique topologies. On the other hand, we extend the known result that the set of executions for systems with only user processes *U* (i.e., without a controller) is $\omega$-regular for clique topologies [36] to homogeneous topologies, and give an effective construction of the corresponding Büchi automaton.

**Disjunctive guards**

*Decidability and complexity*. We show that, similar to pairwise-rendezvous systems, the PMCP is undecidable if we allow 1-index $\textsf {CTL}^*\backslash \textsf {X}$ specifications already for clique topologies, and for 1-index $\textsf {LTL}\backslash \textsf {X}$ specifications already for uni-directional ring topologies. Thus, we restrict our attention to specifications in 1-index $\textsf {LTL}\backslash \textsf {X}$ and homogeneous topologies. We prove that the complexity of the PMCP is Pspace-complete for homogeneous topologies (irrespective of whether or not there is a controller). The program complexity is in Ptime without a controller, and in co-NP with a controller, and is co-NP hard already for the restricted case of a parameterized clique topology.

*Cutoffs*. It is known that cutoffs exist for disjunctively guarded clique topologies and are of size $|U|+2$ [24]. We prove that these cutoffs are tight. We then go on and prove a more general cutoff theorem for disjunctively guarded systems in homogeneous parameterized topologies.

*Equivalent automaton*. We prove that the set of executions is accepted by an effectively constructible Büchi automaton of size $O(|C|^2 \times 2^{|U|})$. It is very interesting to note that this size is smaller than the smallest system size one gets (in the worst-case) from the cutoff result, namely $|C| \times |U|^{|U|+2}$. Hence, the PMCP algorithm obtained from the cutoff is less efficient than the one obtained from going directly to a Büchi automaton. As far as we know, this is the first theoretical proof of the existence of the phenomenon that cutoffs may not yield optimal algorithms. We also prove that, in general, our construction is optimal, i.e., that in some cases every automaton for the set of executions must be of size $2^{\varOmega {(|U|+|C|)}}$.

**Token passing**

In this section we focus on $\textsf {MSO}$-definable sets of topologies of bounded tree-width or clique-width, as well as on iteratively-constructible sets of topologies.

*Decidability and complexity*. We prove that the PMCP is decidable for indexed $\textsf {CTL}^*\backslash \textsf {X}$on such topologies. This considerably generalizes the results of [2], where decidability for this logic was shown for a few concrete topologies such as rings and cliques.

*Cutoffs*. We prove that the PMCPs have *computable* cutoffs for indexed $\textsf {CTL}^*\backslash \textsf {X}$. From [2] we know that there is a (computable) set of topologies and a system template such that there is no algorithm that given an indexed $\textsf {CTL}^*\backslash \textsf {X}$ formula can compute the associated cutoff (even though a cutoff for the given formula exists). This justifies our search of sets of topologies for which the PMCP for $\textsf {CTL}^*\backslash \textsf {X}$ has computable cutoffs. We also give a lower bound on cutoffs for iteratively-constructible sets and indexed $\textsf {LTL}\backslash \textsf {X}$.

*Equivalent automaton*. Our ability to compute cutoffs for 1-index $\textsf {LTL}\backslash \textsf {X}$ formulas implies that the sets of execution traces are $\omega$-regular, and the construction of Büchi automata which compute these traces is effective.

## Definitions and preliminaries

A *labeled transition system (LTS)* is a tuple $(S,R,I,\varPhi ,\textsf {AP},\varSigma )$, where *S* is the set of *states*, $R \subseteq S \times \varSigma \times S$ is the *transition relation*, $I \subseteq S$ are the *initial states*, $\varPhi :S \rightarrow 2^{\textsf {AP}}$ is the *state-labeling*, $\textsf {AP}$ is a set of *atomic propositions* or *atoms*, and $\varSigma$ is the *alphabet of transition labels*. When $\textsf {AP}$ and $\varSigma$ are clear from the context we drop them. A *finite LTS* is an LTS in which $S,R,\varSigma$ are finite and $\varPhi (s)$ is finite for every $s \in S$. Transitions $(s,a,s^{\prime }) \in R$ may be written $s \xrightarrow {a} s^{\prime }$. A *transition system (TS)* $(S,R,I,\varSigma )$ is an LTS without the labeling function and without the set of atomic propositions.

A *path of an LTS* is a finite sequence of the form $s_0 a_0 s_1 a_1 \ldots s_n \in (S \varSigma )^*S$ or an infinite sequence of the form $s_0a_0 s_1 a_1 \ldots \in (S \varSigma )^\omega$ such that $(s_i,a_i,s_{i+1}) \in R$ for all *i*. A *state-labeled path* of an LTS is the projection $s_0 s_1 \ldots$ of a path onto states *S*. An *action-labeled path* of an LTS is the projection $a_0 a_1 \ldots$ of a path onto transition labels $\varSigma$. A *run* is an infinite path that starts in an initial state. Similarly, a *state-labeled run* is a state-labeled path that is infinite and starts in an initial state. However when it is clear from the context we will say ‘run’ instead of ‘state-labeled run’ (e.g., LTL formulas are interpreted over runs $s_0s_1 \ldots$). For a path $\pi$, write $\varPhi (\pi )$ for the induced sequence of labels, i.e., $\varPhi (s_0) \varPhi (s_1) \ldots$.

If $\rho = f_0 f_1 \ldots$ is a sequence of vectors, i.e., $f_i: X \rightarrow Y$ (for some fixed sets *X*, *Y*), and $x \in X$, define the *projection of* $\rho$ *to* *x*, written $proj_x(\rho )$, to be the sequence $f_0(x) f_1(x) \ldots$ of elements of *Y*.

We now introduce notation for automata over infinite and finite strings. For a finite set $\varSigma$, write $\varSigma ^\omega$ for the set of infinite strings over $\varSigma$. Subsets of $\varSigma ^\omega$ are called *languages*. A *(nondeterministic) Büchi word-automaton (NBW)* *A* is a tuple $(\varSigma ,Q,I,\varDelta ,F)$ where $\varSigma$ is a finite *input alphabet*, *Q* is a finite set of *states*, $I \subseteq Q$ is the set of *initial states*, $\varDelta \subseteq Q \times \varSigma \times Q$ is the *transition relation*, and $F \subseteq Q$ are the *accepting states*. A *run* in *A* is an infinite path through *A* that starts in an initial state. The run is *successful* if a state from *F* appears infinitely often. The *language* accepted by the automaton *A* is the set of all infinite strings $\alpha \in \varSigma ^\omega$ that label successful runs in *A*. Languages accepted by NBWs are called $\omega$-*regular*. A *nondeterministic word automaton (NFW)* is similar, except that runs are finite, and a successful run is one that ends in a state of *F*. The language of an NFW is thus a subset of $\varSigma ^*$, i.e., a set of finite strings over alphabet $\varSigma$. Languages accepted by NFWs are called *regular*.

Undecidability proofs will make use of reductions from two-counter machines, known to be Turing powerful [46]. A 2CM is a finite set of *instructions*, say $I_1, \ldots , I_m$, where each instruction is from the following *instruction set*: $\mathsf {HALT}$ (the machine stops when it reaches this instruction), $\mathsf {INC}(i)$ (increment counter *i* by one), $\mathsf {DEC}(i)$ (decrement counter *i* by one), and $\mathsf {JZ}(i, k)$ (if counter *i* is zero then goto instruction $I_k$). Note that after performing each instruction (except for $\mathsf {HALT}$ or a $\mathsf {JZ}$ that performs a goto) the 2CM moves from it’s current instruction $I_j$ to the next instruction $I_{j+1}$. We assume w.l.o.g. (by guarding every decrement with a test for zero) that the machine never tries to decrement a zero counter.

### Process template, topology, pairwise rendezvous system

We define how to (asynchronously) compose processes that communicate via pairwise rendezvous into a single system. We consider discrete time (i.e., not continuous). Processes are not necessarily identical, but we assume there are only a finite number of different process types. Roughly, at every vertex of a topology (a directed graph with vertices labeled by process types) there is a process of the given type running; at every time step, either, and the choice is nondeterministic, exactly one process makes an internal transition, or exactly two processes with an edge between them in the topology perform a synchronizing transition, i.e., they instantaneously synchronize on a message (sometimes called an action) $\mathsf {m} \in \varSigma _{\textsf {sync}}$. The sender of the message $\mathsf {m}$ performs an $\mathsf {m!}$ transition, and the receiver an $\mathsf {m?}$ transition. In this model processes have no IDs, and thus in particular, the sender can not direct the message to a specific neighbouring process (nor can the receiver choose from where to receive it), but the pair is chosen non-deterministically.^5^

In the following we fix a countable set of atoms $\textsf {AP}_{\textsf {pr}}$, as well as a finite synchronization alphabet $\varSigma _{\textsf {sync}}$ (that does not include the symbol $\tau$). Define the *communication alphabet*: $\varSigma = \{\mathsf {m!}, \mathsf {m?} \, \vert \,\mathsf {m} \in \varSigma _{\textsf {sync}}\}$

*Process template and system template* A *pairwise-rendezvous process template* is a finite LTS of the form $P = (S,R,\{\iota \},\varPhi ,\textsf {AP}_{\textsf {pr}},\varSigma \cup \{\tau \})$. Since $\textsf {AP}_{\textsf {pr}}$ and the communication alphabet are typically fixed, we will usually omit them. The *pairwise-rendezvous system arity* is a natural number $r \in \mathbb {N}$. It refers to the number of different process types in the system. We call the transitions of a process template *local transitions*. A *pairwise-rendezvous* *r*-*ary system template* is a tuple of process templates $\overline{P}= (P_1,\ldots ,P_r)$ where *r* is the system arity. The process template $P_i = (S_i,R_i,\{\iota _i\},\varPhi _i)$ is called the *i* *th process template*. We sometimes drop the adjectives “*r*-ary” and “pairwise-rendezvous”.

*Topology* An *r* *-topology* is a finite structure $G= (V,E, T_1,\ldots ,T_r)$ where $E \subseteq V \times V$, and the $T_i \subseteq V$ partition *V*.^6^ The *type* of $v \in V$ denoted *type*(*v*) is the unique $j \le r$ such that $v \in T_j$. We might write $V_G, E_G$ and $type_G$ to stress $G$.

We write [*n*] to denote the set $\{1, \ldots , n\}$, for any $n \in \mathbb {N}$. We sometimes assume that $V {:=} [n]$ for some $n \in \mathbb {N}$. For instance the 1*-ary ring topology* with $V = \{1,\ldots , n\}$ has $E = \{(i,j) \in [n]^2 \, \vert \,j = i + 1 \mod n\}$ and $T_1 = V$.

*Pairwise-rendezvous system* Given a system arity *r*, a system template $\overline{P}= (P_1,\ldots ,P_r)$ with $P_i = (S_i,R_i,\{\iota _i\},\varPhi _i)$, and an *r*-topology $G= (V,E,\overline{T})$, define the *system* $\overline{P}^G$ as the LTS $(Q,\varDelta ,Q_0,\varLambda ,\textsf {AP}_{\textsf {pr}}\times V, \varSigma _{\textsf {sync}}\cup \{\tau \})$ where

- The set *Q* is the set of functions $f:V \rightarrow \cup _{i \le r} S_i$ such that $f(v) \in S_i$ iff $type(v) = i$ (for $v \in V, i \le r$). Such functions (sometimes written as vectors) are called *configurations*.
- The set $Q_0$ consists of the unique *initial configuration* $f_\iota$ defined as $f_\iota (v) = \iota _{type(v)}$ (for all $v \in V$).
- The set of *global transitions* $\varDelta$ are tuples $(f,\mathsf {m},g) \in Q \times (\varSigma _{\textsf {sync}}\cup \{\tau \}) \times Q$ where one of the following two conditions hold:
  - $\mathsf {m} = \tau$ and there exists $v \in V$ such that $f(v) \xrightarrow {\tau } g(v)$ is a transition of the process template $P_{type(v)}$, and for all $w \ne v$, $f(w) = g(w)$; this is called an *internal transition*,
  - $\mathsf {m} \in \varSigma _{\textsf {sync}}$ and there exists $v \ne w \in V$ with $(v,w) \in E$ such that $f(v) \xrightarrow {m!} g(v)$ is a transition of $P_{type(v)}$ and $f(w) \xrightarrow {m?} g(w)$ is a transition of $P_{type(w)}$ and for all $z \notin \{v,w\}$, $f(z) = g(z)$; this is called a *synchronous transition*. We say that the process at *v* *sends the message* $\mathsf {m}$ and the process at *w* *receives the message* $\mathsf {m}$.
- The labeling function $\varLambda :Q \rightarrow 2^{\textsf {AP}_{\textsf {pr}}\times V}$ is defined by $(p,v) \in \varLambda (f) \iff p \in \varPhi _{type(v)}(f(v))$ (for configurations *f*, atoms $p \in \textsf {AP}_{\textsf {pr}}$ and vertices $v \in V$).

In words then, a topology of size *n* specifies *n*-many processes, which processes have which type, and how the processes are connected. In the internal transition above only the process at vertex *v* makes a transition, and in the synchronous transition above only the process at vertex *v* and its neighbour at *w* make a transition. Let $\pi = f_0 f_1 \ldots$ be a state-labeled path in $\overline{P}^G$. The projection of $\pi$ to vertex $v \in V$, denoted $proj_v(\pi )$, is the sequence $f_0(v) f_1(v) \ldots$ of states of $P_{type(v)}$. If $type(v) = j$ we say that the *vertex* *v* *runs (a copy of) the process* $P_j$, or that *the process template at* *v* *is* $P_j$. We sometimes drop the adjective “pairwise-rendezvous” and simply talk about a system.

### Disjunctively-guarded systems and token passing systems

We define disjunctively-guarded systems and token-passing systems as restricted forms of pairwise rendezvous systems. In fact, the restrictions are on the synchronization alphabet, the system template, and in case of token passing systems also on the topology. Write $P_{i}=(S_{i},R_{i},\{\iota _{i}\},\varPhi _{i},\textsf {AP}_{\textsf {pr}},\varSigma \cup \{\tau \})$.

*Disjunctively-guarded system*. A *disjunctively-guarded system template* is a system-template ${\mathcal {P}}$ such that

- The synchronization alphabet is $\varSigma _{\textsf {sync}}$ is $\cup _{i \le r} S_i$, and the communication-alphabet $\varSigma$ is $\{\tau \} \cup \{\mathsf {q!}, \mathsf {q?} \, \vert \,q \in \cup S_i\}$.
- The state sets of the process templates are pairwise disjoint, i.e., $S_i \cap S_j = \emptyset$ for $1 \le i < j \le r$.
- For every state $s \in S_i$ ($i \le r$), there is a transition in $S_i$ labeled $s \xrightarrow {\mathsf {s?}} s$.
- For every state $s \in S_i$ ($i \le r$), the only transitions in $S_i$ labeled $\mathsf {s?}$ are of the form $s \xrightarrow {\mathsf {s?}} s$.

Observe that a process can take a transition $q \xrightarrow {\mathsf {s!}} q^{\prime }$ iff there is some other process in state *s*, and that the receiver of a message *s* stays in state *s*. We say that the transition $q \xrightarrow {\mathsf {s!}} q^{\prime }$ is *guarded* by the state *s*. We say that a process in a disjunctively-guarded system that is in a state *s* *opens* the gate *s*.

In the following, given any pair of states *q* and $q^{\prime }$, and given some finite set of states $Y = \{y_1, \ldots , y_n\}$, we usually write $q \xrightarrow {Y} q^{\prime }$ instead of writing the multiple transitions $q \xrightarrow {\mathsf {y!}} q^{\prime }$ for $y \in Y$. We usually also forgo writing the $\tau$-label (and thus write $q \xrightarrow {} p$ instead of $q \xrightarrow {\tau } p$).

A *disjunctively-guarded system* is a system formed using disjunctively-guarded system templates. Our definition of disjunctively-guarded systems on a clique topology is a reformulation of the definition of concrete system in [24, Section 2]: there, local transitions of process templates can be guarded by disjunctive boolean formulas that observe the local state of some other process. In our encoding, the *observer* and the *observed* processes synchronize: the former does the desired local transition, while the latter self-loops, not changing its local state. For this encoding to work, we require that the transition labels have the same name of the local states of the observed process templates.

*Token passing system*. In this work we only consider the case of token passing systems (TPS) with a single valueless token [2, 28]. A *token passing system template* is a system template ${\mathcal {P}}$ such that

- $\varSigma _{\textsf {sync}}=\{\mathsf {tok}\}$, i.e., the only synchronization operation is passing the token.
- The system arity *r* satisfies $r \ge 2$.
- Every set $S_{i}$ is partitioned into $S_{i}^{\mathsf {tok}}\subseteq \{\mathsf {tok}\}\times \mathbb {N}$ and $S_{i}^{\mathsf {ntok}}\subseteq \{\mathsf {ntok}\}\times \mathbb {N}$. We think of $S_{i}^{\mathsf {tok}}$ (resp. $S_{i}^{\mathsf {ntok}}$) as the states in which the process has (resp. does not have) the token.
- If $(s_{1},\tau ,s_{2})\in R_{i}$, then $s_{1},s_{2}\in S_{i}^{\mathsf {tok}}$ or $s_{1},s_{2}\in S^{\mathsf {ntok}}$, i.e., internal transitions do not affect whether the process has the token.
- If $(s_{1},\mathsf {tok}!,s_{2})\in R_{i}$, then $s_{1}\in S_{i}^{\mathsf {tok}}$ and $s_{2}\in S_{i}^{\mathsf {ntok}}$, i.e., $\mathsf {tok}!$ is the action of *token sending*.
- If $(s_{1},\mathsf {tok}?,s_{2})\in R_{i}$, then $s_{1}\in S_{i}^{\mathsf {ntok}}$ and $s_{2}\in S_{i}^{\mathsf {tok}}$. i.e., $\mathsf {tok}?$ is the action of *token receiving*.
- $\iota _{1}\in S_{1}^{\mathsf {tok}}$ and for every $i>1$, $\iota _{i}\in S_{i}^{\mathsf {ntok}}$, i.e., a process with template $P_1$ starts with the token.

A *token passing system* is a system formed using a token passing system template and a topology *G* such that $|T_{1}|=1$, i.e., exactly one process can start with the token.

Intuitively, at any time during the computation, exactly one vertex has the token. The token starts with the unique process $P_{1}$, and later may be passed to processes in $P_{2}, \ldots , P_{r}$. This means that the token passing systems considered in this work inherently requires *topologies with controllers* (see Sect. 2.8 for details). At each time step either exactly one process makes an internal transition, or exactly two processes synchronize when one process sends the token to another along an edge of *G*.

### Parameterized topologies

*Parameterized topology*. An *r*-*ary parameterized topology* ${\mathcal G}$ is a set of *r*-topologies such that membership in ${\mathcal G}$ is decidable. We may drop the adjective “*r*-ary”. The following are typical examples of parameterized topologies.

- The set of all 1-ary ring topologies.
- The set of all *r*-ary clique topologies.
- The set of all 2-ary ring topologies $(V,E,T_1,T_2)$ such that $|T_1| = 1$. In a given topology of this form, the unique process at the vertex of type 1 is called a *controller*, and the processes at the vertices of type 2 are called *users*. See Sect. 2.8 for more on controllers and users.

*Homogeneous parameterized topology*. We now define the homogeneous parameterized topologies which generalize the clique parameterized topologies.

An *r*-ary parameterized topology ${\mathcal G}$ is *homogeneous* if there is a directed graph *H* with vertex set $V_H = [r]$ and edge set $E_H$ and a partition $B_{sng},B_{clq},B_{ind}$ of [*r*] such that an *r*-ary topology $G = (V,E,T_1,\ldots ,T_r) \in {\mathcal G}$ if and only if

1. For every $i \in B_{sng}$ there exists a unique $v \in V$ such that $v \in T_i$;
2. For every $i \in B_{clq}$ and $u,v \in T_i$: *E*(*u*, *v*) and *E*(*v*, *u*);
3. For every $i \in B_{ind}$ and $u,v \in T_i$: $\lnot E(u,v)$ and $\lnot E(v,u)$;
4. For every $i \ne j \in V_H$ and $u \in T_i, v \in T_j$: *E*(*u*, *v*) if and only if $E_H(i,j)$.

In other words, *G* is formed from *H* by substituting each vertex in $B_{clq}$ with a *clique*, each vertex in $B_{ind}$ with an *independent* set, leaving every vertex in $B_{sng}$ as a *single* vertex, and connecting vertices as they were connected in *H*.

We say that ${\mathcal G}$ is *generated by* *H* *and* $B_{sng},B_{clq},B_{ind}$. The cardinality of $B_{sng}$ is the *number of controllers* in ${\mathcal G}$. In case $B_{sng} = \emptyset$ we say that ${\mathcal G}$ is *controllerless*, and otherwise we say that ${\mathcal G}$ is *controlled*. If $B_{ind} = \emptyset$ and *H* is a clique, then we say that ${\mathcal G}$ is an *r* *-ary clique parameterized topology*. If $B_{ind} = B_{sng} = \emptyset$ then ${\mathcal G}$ is called *the* *r* *-ary controllerless-clique parameterized topology*.

We now give some examples. Fix the 2-topology *H* with vertex set $V_H = \{1,2\}$ and edge set $\{(1,2),(2,1)\}$ and $type(i) = i$ for $i \in [2]$.

*Example* (**Cliques**) The set of 2-ary cliques in which exactly one index has type 1 is homogeneous generated by the *H* above, $B_{clq}=\{2\}$, $B_{ind}=\emptyset$ and $B_{sng}=\{1\}$.

*Example* (**Stars**) The set of stars in which exactly one index has type 1 is homogeneous using *H* above, $B_{clq}=\emptyset$, $B_{ind}=\{2\}$ and $B_{sng} = \{1\}$.

*Example* (**Bipartite graphs**) The set of topologies that are complete bipartite graphs is homogeneous using *H* above, $B_{ind}=\{1,2\}$, and $B_{clq}=B_{sng}=\emptyset$.

*Example* (**Rings are not homogeneous**) The length of the longest simple path in any member of an homogeneous parameterized topology generated by *H* is at most the number of vertices in *H*. Thus the set of rings is not homogeneous (for any *H*).

Call a homogeneous parameterized topology *H* *non-trivial* if there exists $i \in B_{sng}$ and $j \in B_{clq} \cup B_{ind}$ such that $(i,j) \in E_H$ and $(j,i) \in E_H$. Informally, a non-trivial homogeneous parameterized topology means that there is bi-directional communication between a controller and arbitrarily many user processes. This notion is used for some of the lower-bounds in Sects. 3 and 4.

### Indexed temporal logic

We assume the reader is familiar with the syntax and semantics of $\textsf {CTL}^*$ and LTL, see e.g., [11]. Indexed temporal logics were introduced by [15] to model specifications of certain distributed systems. They are obtained by adding *vertex quantifiers* to a given temporal logic over indexed atomic propositions. For example, in a system with two process templates, the formula  states that every process of type 1 on all computations at all points of time satisfies the atom *good*. In a system with one process template, the formula  states that it is never the case that two processes both satisfy the atom  at the same time.^7^

*Syntax* Fix an infinite set $\textsf {Vars}= \{i,j,\ldots \}$ of vertex variables (called index variables). A *vertex quantifier* is an expression of the form $\exists x:type(x)=m$ or $\forall x: type(x)=m$ where $m \in \mathbb {N}$. An *indexed* $\textsf {CTL}^*$ *formula over vertex variables* $\textsf {Vars}$ *and atomic propositions* $\textsf {AP}_{\textsf {pr}}$ is a formula of the form $Q_1 i_1, \ldots , Q_k i_k .\, \varphi$, where each $i_n \in \textsf {Vars}$, each $Q_{i_n}$ is an vertex quantifier, and $\varphi$ is a $\textsf {CTL}^*$ state formula over atomic predicates $\textsf {AP}_{\textsf {pr}}\times \textsf {Vars}$.

*Semantics* An indexed $\textsf {CTL}^*$ formula $\phi$ is interpreted over a system $\overline{P}^G= (Q,\varDelta ,\{f_\iota \},\varLambda ,\textsf {AP}_{\textsf {pr}}\times V, \varSigma _{\textsf {sync}}\cup \{\tau \})$ (for *r*-ary system template $\overline{P}$ and *r*-topology $G= (V,E,\overline{T})$). A *valuation* is a function $e:\textsf {Vars}\rightarrow V$.

For some state formula $\phi$, and valuation *e* define $(\overline{P}^G,e,q) \models \phi$ inductively. Path formulas are interpreted similarly, but over $(\overline{P}^G,e,\pi )$, where $\pi$ is a run of $\overline{P}^G$. The only new cases are the *base case* and the *quantifier case*.

Base case: For $(p,i) \in \textsf {AP}_{\textsf {pr}}\times \textsf {Vars}$ define $(\overline{P}^G,e,q) \models (p,i)$ to mean that $(p,e(i)) \in \varLambda (q)$. In words, the atom *p* holds at the state of the process at vertex $e(i) \in V$.
Quantifier case: An *i*-*variant* of a valuation *e* is a valuation $e^{\prime }$ with $e^{\prime }(j)=e(j)$ for all $j \in \textsf {Vars}$ with $j \ne i$. Define  to mean that for all *i*-variants $e^{\prime }$ of *e*, if $type_G(e^{\prime }(i)) = m$ then $(\overline{P}^G,e^{\prime },q) \models \phi$. The semantics of the quantifiers  are defined similarly.

Finally, define $\overline{P}^G\models \phi$ if it holds $(\overline{P}^G, f_\iota ) \models \phi$ for the initial state $f_\iota$ of $\overline{P}^G$.

#### Notation

In the rest of the paper we will apply the following conventions, for the sake of readability.

- An atom $(p,j) \in \textsf {AP}_{\textsf {pr}}\times \textsf {Vars}$ is sometimes also written as $p_j$.
- A formula  is called a *sentence* if for every atom $(p,j) \in \textsf {AP}_{\textsf {pr}}\times \textsf {Vars}$ that occurs in the formula, there is a quantifier that binds *j*, that is, $j \in \{i_1, \ldots , i_k\}$.
- The formula is called *universal* (resp. *existential*) if all the vertex quantifiers $Q_1 i_1, \ldots , Q_k i_k$ are universal (resp. existential).
- For $k > 1$ we allow more general quantification such as $\forall i \ne j$. The semantics are defined in the natural way, see the full version of [2].
- In case of 1-ary systems, we may write $\forall x$ instead of $\forall x: type(x) = 1$.
- In the syntax of indexed formulas we sometimes write $type(x) = P_m$ instead of $type(x) = m$.
- Write ${\textsf {i-CTL}}^*$ for the set of all indexed $\textsf {CTL}^*$ sentences, and $\text{ k- }\textsf {CTL}^*$ for the set of all *k*-indexed formulas in ${\textsf {i-CTL}}^*$, i.e., formulas with *k* many quantifiers. Write $\textsf {CTL}^*_d$ for the fragment of $\textsf {CTL}^*$ with path-quantifier nesting-depth at most *d* [49]. We similarly define indexed versions of the various natural fragments of $\textsf {CTL}^*$, e.g., ${\textsf {i-LTL}}$, $\text{ k- }{\textsf {LTL}}\backslash \textsf {X}$ and $\text{ k- }\textsf {CTL}^*_d\backslash \textsf {X}$.
- Say that $\overline{P}^{G}$ is $\text{ k- }\textsf {CTL}^*_d\backslash \textsf {X}$-*equivalent* to $\overline{P}^{G^{\prime }}$, written $\overline{P}^{G} \equiv _{\text{ k- }\textsf {CTL}^*_d\backslash \textsf {X}} \overline{P}^{G^{\prime }}$, if they agree on all $\text{ k- }\textsf {CTL}^*_d\backslash \textsf {X}$ formulas: for every $\text{ k- }\textsf {CTL}^*_d\backslash \textsf {X}$ formula $\phi$ it holds that $\overline{P}^{G} \models \phi$ iff $\overline{P}^{G^{\prime }} \models \phi$.

*Note*. The index variables are bound *outside* of all the temporal path quantifiers ($\textsf {A}$ and $\textsf {E}$). In particular, for an existentially quantified ${\textsf {LTL}}$ formula to be satisfied there must exist a valuation of the index variables such that $\phi$ holds for all runs (and not one valuation for each run). Thus this logic is sometimes called *prenex indexed temporal logic*. Note that if one allows vertex quantifiers inside the scope of temporal path quantifiers then one quickly reaches undecidability even for systems with no communication [41].

For the remainder of this paper specifications only come from ${\textsf {i-CTL}}^*\backslash \textsf {X}$, i.e., without the next-time operator $\textsf {X}$. It is usual in the context of parameterized systems to consider specification logics without the next-time operator. The reason is that since we discretize time, when a process makes an internal transition time proceeds by one step. However, a formula that talks about one processes should usually not be able to (as $\textsf {X}$ allows) refer to the time advance caused by other processes making internal moves.

### The parameterized model checking problem

Fix an *r*-ary parameterized topology ${\mathcal G}$, a set of *r*-ary system templates ${\mathcal {P}}$, and a set of indexed temporal logic sentences ${\mathcal F}$. The **parameterized model checking problem (PMCP)**, written ${\textsf {PMCP}}_{{\mathcal G}}({\mathcal {P}},{\mathcal F})$, is to decide, given a formula $\varphi \in {\mathcal F}$ and a system template $\overline{P}\in {\mathcal {P}}$, whether for all $G\in {\mathcal G}$, $\overline{P}^G\models \varphi$. The complexity of the ${\textsf {PMCP}}_{{\mathcal G}}({\mathcal {P}},{\mathcal F})$, where the formula $\varphi \in {\mathcal F}$ is fixed and only the system template is given as an input, is called the *program complexity*.

### Process executions

In this section we define process executions, and show in Lemma 2 how to reduce reasoning about Indexed Temporal Logic over homogeneous parameterized topologies to reasoning about process executions. In Corollary 1 we show how to apply the automata-theoretic approach to reasoning about the PMCP, and in Lemma 3 we show how to reduce homogeneous systems to clique systems.

First we define the destuttering of a word, which roughly means that one removes identical consecutive letters. The *destuttering of a word* $\alpha \in \varSigma ^\omega \cup \varSigma ^*$ is the word $\alpha ^\delta \in \varSigma ^\omega \cup \varSigma ^*$, also denoted $\mathsf {destutter}(\alpha )$, defined by replacing every maximal finite consecutive sequence of repeated symbols in $\alpha$ by one copy of that symbol. Note that if $\alpha$ is infinite, then $\alpha ^\delta$ is also infinite. Thus, the destuttering of $(aaba)^\omega$ is $(ab)^\omega$; the destuttering of $aab^\omega$ is $ab^\omega$; and the destuttering of $(aaba)^k$ is $(ab)^ka$. The *destuttering of the set* $L \subseteq \varSigma ^\omega$, written $L^\delta$, is the set $\{\alpha ^\delta \, \vert \,\alpha \in L\}$. The *stuttering closure* of *L*, written $L^{\delta c}$, is the set $\{\alpha \, \vert \,\alpha ^\delta \in L^\delta \}$.

#### Lemma 1

If $L \subseteq \varSigma ^\omega$ is $\omega$-regular then so are the sets $L^{\delta c}$ and $L^\delta$. Moreover, let *Q* be the set of states of the Büchi automaton for *L*, then $L^\delta$ and $L^{\delta c}$ are recognized by a Büchi automaton in time $O(|Q| \times |\varSigma |)$.

#### Proof

Given a Büchi automaton *A* recognizing *L* with states *Q*, we first obtain an automaton $A^{\prime }$ (with states $Q \times \varSigma$) also recognizing *L* by refining the states of *A* to remember the last symbol read. We can obtain an automaton *B* for the stuttering closure $L^{\delta c}$ by adding transitions to $A^{\prime }$: for every state (*q*, *a*) of $A^{\prime }$ add the transition $(q,a) \xrightarrow {a} (q,a)$; and for every transition of $A^{\prime }$ of the form $(q,a) \xrightarrow {a} (q^{\prime },a)$ add the epsilon-transition $(q,a) \xrightarrow {\epsilon } (q^{\prime },a)$. To obtain an automaton for the destuttering $L^\delta$, intersect *B* with an automaton for those strings $\beta \in \varSigma ^\omega$ such that if $\beta _i = \beta _{i+1}$ then $\beta _i = \beta _j$ for all $j > i$. $\square$

It is known that $\textsf {LTL}\backslash \textsf {X}$ cannot distinguish between a word and its destuttering (this follows, e.g., from [49]), which is the main motivation for the next definition.

*Process executions*. Fix an *r*-ary parameterized topology ${\mathcal G}$ and an *r*-ary system template $\overline{P}= (P_1,\ldots ,P_r)$. Define the set of *(process) executions* associated with a *r*-topology $G\in {\mathcal G}$ and vertex $v \in V_G$:

$$\begin{aligned} \begin{array}{l} \textsc {exec}_{G}(\overline{P},v) {:=} \{\mathsf {destutter}(proj_v(\pi )) \, \vert \,\\ \pi \text { is a state-labelled run of } \overline{P}^G\}. \end{array} \end{aligned}$$

For $t \le r$, define the *t-process executions* in $G$ to be the union of all process executions associated with vertices of type *t*:

$$\begin{aligned} \textsc {t-exec}_{G}{(\overline{P})} {:=} \bigcup \{\textsc {exec}_{G}(\overline{P},v) \, \vert \,v \in V_G, type(v) = t\}. \end{aligned}$$

Finally, define the set of t-process executions of the parameterized topology ${\mathcal G}$:

$$\begin{aligned} \textsc {t-exec}_{{\mathcal G}}{(\overline{P})} {:=} \bigcup _{G\in {\mathcal G}} \textsc {t-exec}_{G}{(\overline{P})}. \end{aligned}$$

Intuitively, $\textsc {t-exec}_{{\mathcal G}}{(\overline{P})}$ contains all sequences of $P_t$ states visited by some instance of $P_t$ along some run. When ${\mathcal G}$ or $\overline{P}$ are clear from the context we may omit them.

The following lemma says that we can reduce PMCP of 1-index $\textsf {LTL}\backslash \textsf {X}$ to model checking an ordinary $\textsf {LTL}\backslash \textsf {X}$ formula over the set $\textsc {t-exec}_{{\mathcal G}}{(\overline{P})}$. Although we state it for pairwise-rendezvous system templates, its proof only uses symmetry (i.e., on a homogeneous topology $G$, we have that $\textsc {t-exec}_{G}{(\overline{P})}$ is equal to the set of executions $\textsc {exec}_{G}(\overline{P},v)$ of any single process *v* of type *t*), and the fact that $\textsf {LTL}\backslash \textsf {X}$ can not distinguish between a word and its destuttering.

#### Lemma 2

Fix an *r*-ary homogeneous parameterized topology ${\mathcal G}$, pairwise-rendezvous system template $\overline{P}$, and 1-index $\textsf {LTL}\backslash \textsf {X}$ sentence of the form $\psi = Q x : type(x) = t {.\, \phi }$ (for $Q \in \{\exists , \forall \}$ and $t \le r$). Let $\phi ^{\prime }$ be the $\textsf {LTL}\backslash \textsf {X}$ formula in which every atom in $\phi$ of the form (*a*, *x*) has been replaced by the atom $a \in \textsf {AP}_{\textsf {pr}}$. The following are equivalent:

1. $\forall \pi \in \textsc {t-exec}_{{\mathcal G}}{(\overline{P})}$ we have that $\pi \models \phi ^{\prime }$,
2. For all $G\in {\mathcal G}$ and all $v \in V_G$ of type *t* in $G$, and all $\pi \in \textsc {exec}_{G}(\overline{P},v)$, we have that $\pi \models \phi ^{\prime }$.
3. $$\forall G\in {\mathcal G}, \overline{P}^G\models \forall x : type(x) = t {.\, \phi }$$
4. $$\forall G\in {\mathcal G}, \overline{P}^G\models \exists x : type(x) = t {.\, \phi }$$

#### Proof

It is easy to see that, for the case of a clique topology $G$, the truth value of a 1-indexed $\textsf {LTL}\backslash \textsf {X}$ sentence of the form $\forall x : type(x) =t {.\, \phi }$ depends only on the set $\textsc {t-exec}_{{\mathcal G}}{(\overline{P})}$. Observe that, on a homogeneous topology $G$, due to symmetry, we have that $\textsc {t-exec}_{G}{(\overline{P})}$ is equal to the set of executions $\textsc {exec}_{G}(\overline{P},v)$ of any single process *v* of type *t*. Indeed, if $v,v^{\prime }$ are both of type *t*, then for every run $\pi$ of $\overline{P}^G$, there is a run $\pi ^{\prime }$ of $\overline{P}^G$, such that $proj_v(\pi ) = proj_{v^{\prime }}(\pi ^{\prime })$. The run $\pi ^{\prime }$ is obtained by simply replacing the roles of *v* and $v^{\prime }$ on $\pi$ (i.e., by replacing any local transition of *v* with one of $v^{\prime }$, and vice-versa). This is possible since, being of the same type *t*, the vertices $v,v^{\prime }$ have exactly the same neighbouring vertices. $\square$

In the automata-theoretic approach to model checking [53], one reduces the question of whether every execution of a system satisfies an ${\textsf {LTL}}$ formula $\phi$ to the emptiness of the intersection of *A* and $A_{\lnot \phi }$, where *A* is a non-deterministic Büchi word automaton (NBW) accepting the set of executions of the system and $A_{\lnot \phi }$ is an NBW accepting the set of words for which $\lnot \phi$ holds. Together with Lemma 2, this gives us the following corollary:

#### Corollary 1

Assume the hypotheses of Lemma 2. Then $[\forall G\in {\mathcal G}\cdot ~ \overline{P}^G\models \psi ]$ if and only if the intersection of *A* and $A_{\lnot \phi ^{\prime }}$ is empty, where *A* is an NBW for the language $\{\varPhi (\alpha ): \alpha \in \textsc {t-exec}_{{\mathcal G}}{(\overline{P})}\}$ (and $\varPhi$ is the state-labeling of the process template with type *t*).

*From homogeneous to clique*. We show a general reduction from homogeneous systems to clique systems. Moreover, if the homogeneous system is controllerless then so is the clique-system. Recall that homogeneous parameterized topologies are generated by an *r*-ary topology *H* (where each vertex has a unique type) and a partition $B_{ind},B_{clq},B_{sng}$ of [*r*].

#### Lemma 3

Let ${\mathcal G}$ be an *r*-ary homogeneous parameterized topology, and let $\overline{P}= (P_1,\ldots ,P_r)$ be a pairwise-rendezvous *r*-ary system template.

1. If ${\mathcal G}$ is controllerless, then there exists a pairwise-rendezvous template $\overline{P}^{\prime }$ such that $\textsc {1-exec}_{{\mathcal G}^\prime }{(\overline{P}^{\prime })} = \bigcup \{\textsc {i-exec}_{{\mathcal G}}{(\overline{P})}: i \in B_{clq} \cup B_{ind}\}$, where ${\mathcal G}^\prime$ is the 1-ary controllerless-clique parameterized topology. Also, $|\overline{P}^{\prime }| = \sum _i |P_i|$.
2. If ${\mathcal G}$ is controlled, then there exists a pairwise-rendezvous 2-ary system template $\overline{P}^{\prime } = (P^{\prime }_1,P^{\prime }_2)$ and a 2-ary controlled-clique parameterized topology ${\mathcal G}^\prime$, such that $\textsc {1-exec}_{{\mathcal G}^\prime }{(\overline{P}^{\prime })}$ is equal to $\bigcup \{\textsc {i-exec}_{{\mathcal G}}{(\overline{P})}: i \in B_{clq} \cup B_{ind}\}$, and for every $i \in B_{sng}$,
  $$\begin{aligned} \textsc {i-exec}_{{\mathcal G}}{(\overline{P})} = \mathsf {destutter}(proj_i(\textsc {2-exec}_{{\mathcal G}^{\prime }}{(\overline{P}^{\prime })})). \end{aligned}$$
  Moreover, $|P^{\prime }_1|$ is equal to $\sum \limits _{i \in B_{clq} \cup B_{ind}} |P_i|$ and $|P^{\prime }_2|$ is equal to $\prod \limits _{i \in B_{sng}}|P_i|$.
3. If $\overline{P}$ is a disjunctively-guarded system template, then also the system template $\overline{P}^{\prime }$ in items 1 and 2 above can be taken to be disjunctively-guarded.

#### Proof

We prove the controlled case (the controllerless case is simpler). Given an *r*-ary system template $\overline{P}$, let the controlled homogeneous parameterized topology ${\mathcal G}$ be generated by $H = (V_H,E_H)$ and some partition $B_{sng}$, $B_{clq}$, $B_{ind}$ of $V_H$.

The basic idea behind the reduction is that the process template $P^{\prime }_1$ is the disjoint union of the $P_i$ with $i \in B_{clq} \cup B_{ind}$, and the process template $P^{\prime }_2$ is the product of the $P_i$ with $i \in B_{sng}$. Thus, every process running $P^{\prime }_1$ can nondeterministically decide (by starting in the corresponding component) which of the $P_i$s to simulate, and the single process running $P^{\prime }_2$ simulates all of the $P_i$s with $i \in B_{sng}$. Note that (by definition of homogeneous) every $G \in {\mathcal G}$ is formed from some function $S_G: V_H \rightarrow \mathbb {N}$ such that every vertex *i* in *H* is replaced with a clique (if $i \in B_{clq}$) or an independent set (if $i \in B_{ind}$), of size $S_G(i)$. In this case let $G^{\prime }$ be the 2-ary clique of size $1+\varSigma _{i=1}^r S_G(i)$ (i.e., it has one vertex of type 1, and the rest are of type 2). Let ${\mathcal G}^{\prime }$ be the set of all such $G^{\prime }$ and note that ${\mathcal G}^{\prime }$ is a 2-ary controlled clique parameterized topology. We can simulate every computation of $\overline{P}^G$ by a corresponding computation of ${\overline{P}^\prime }^{G^{\prime }}$, and vice versa, in which, for every $i \in B_{clq} \cup B_{ind}$, exactly $S_G(i)$ processes make the nondeterministic choice to use the portion of $P_1^\prime$ that is $P_i$. Observe that, in $G$, a process associated with a vertex $i \in V_H$ can send a message $\mathsf {m}$ to another process, which is associated with a vertex *j* of *H*, if and only if either $(i,j) \in E_H$, or, $i \in B_{clq}$ and $i=j$. We mirror this restriction in ${\overline{P}^\prime }^{G^{\prime }}$ as follows: in $P_1^\prime$, every state that is in the $P_i$-component attaches *i* to every message that it sends, and for every $j \in B_{clq} \cup B_{ind}$, a state that is in the $P_j$-component can receive a message $\mathsf {(m,i)}$ if and only if either $(i,j) \in E_H$, or, $i=j$ and $j \in B_{clq}$. Similarly, in $P_2^{\prime }$, the *i*th co-ordinate (for $i \in B_{sng}$) attaches *i* to the messages it sends, and for every $j \in B_{sng}$, the *j*th co-ordinate of $P_2^{\prime }$ can receive message $\mathsf {(m,i)}$ if and only if $(i,j) \in E_H$.

Formally, suppose (for $i \in V_H$) that $P_i$ is the *i*th template in $\overline{P}$, say with state set $S_i$. Assume w.l.o.g. that $S_i \cap S_j = \emptyset$ for all $i \ne j$, and that $\varSigma = \{ \mathsf {m!}, \mathsf {m?} \, \vert \,\mathsf {m} \in \varSigma _{\textsf {sync}}\}$. Define the state set of $P_1^\prime$ to be the disjoint union of the $S_i$s for $i \in B_{clq} \cup B_{ind}$. Thus $P_1^\prime$ has multiple initial states.^8^ Define the state set of $P_2^\prime$ to be the product of the $S_i$s for $i \in B_{sng}$. Formally, the states are functions $t:B_{sng} \rightarrow \cup _{i \in B_{sng}} S_i$ such that $t(i) \in S_i$. The initial state of $P^{\prime }_2$ is the function $\iota ^{\prime }_2$ assigning to each $i\in B_{sng}$ the initial state of $P_i$.

The communication alphabet is the set $\varSigma ^{\prime } {:=} \{\mathsf {(m,i)!}, \mathsf {(m,i)?} \, \vert \,\mathsf {m} \in \varSigma _{\textsf {sync}}, i \in V_H\}$. The transitions of $\overline{P}^G$ are simulated by ${\overline{P}^\prime }^{G^{\prime }}$ below. In the following, transitions sending a message from *i* to *j* are simulated according to the sets $B_{ind},B_{clq},B_{snd}$ to which *i* and *j* belong: transitions from $i \notin B_{sng}$ to $j \in B_{sng}$ are simulated in items (A) and (D); transitions from $i \in B_{sng}$ to $j \notin B_{sng}$ are simulated in items (B) and (C); transitions from $i \notin B_{sng}$ to $j \notin B_{sng}$ are simulated in items (A) and (C); and transitions from $i \in B_{sng}$ to $j \in B_{sng}$ are simulated in item (E). Internal transitions are simulated in items (F) and (G). The transitions from the dummy initial state of $P^{\prime }_1$ to the initial states of $i\notin B_{sng}$ are given in item (H).

(A) If $i \in B_{ind} \cup B_{clq}$, and $s \xrightarrow {\mathsf {m!}} s^{\prime }$ is a transition of $P_i$, then $s \xrightarrow {\mathsf {(m,i)!}} s^{\prime }$ is a transition of $P_1^\prime$;
(B) If $i \in B_{sng}$, and $s \xrightarrow {\mathsf {m!}} s^{\prime }$ is a transition of $P_i$, then $t \xrightarrow {\mathsf {(m,i)!}} t^{\prime }$ is a transition of $P^{\prime }_2$ where $t(i) = s, t^{\prime }(i)=s^{\prime }$ and $t(l) = t^{\prime }(l)$ for $l \ne i$;
(C) if $i=j \in B_{clq}$, or $(i,j) \in E_H$ for $i \in V_H, j \in B_{ind} \cup B_{clq}$, and $s \xrightarrow {\mathsf {m?}} s^{\prime }$ is a transition of $P_{j}$, then $s \xrightarrow {\mathsf {(m,i)?}} s^{\prime }$ is a transition of $P_1^\prime$;
(D) if $(i,j) \in E_H$, $i \in B_{ind}\cup B_{clq}$, $j \in B_{sng}$, and $s \xrightarrow {\mathsf {m?}} s^{\prime }$ is a transition of $P_{j}$, then $t \xrightarrow {\mathsf {(m,i)?}} t^{\prime }$ is a transition of $P_2^\prime$ where $t(j) = s, t^{\prime }(j)=s^{\prime }$ and $t(l) = t^{\prime }(l)$ for $l \ne j$;
(E) If $i\not =j \in B_{sng}$, and $s \xrightarrow {\mathsf {m!}} s^{\prime }$ is a transition of $P_i$, and $r \xrightarrow {\mathsf {m?}} r^{\prime }$ is a transition of $P_j$, then $t \xrightarrow {\tau } t^{\prime }$ is a transition of $P^{\prime }_2$ where $t(i) = s, t^{\prime }(i)=s^{\prime }$, $t(j) = r, t^{\prime }(j)=r^{\prime }$, and $t(l) = t^{\prime }(l)$ for $l \not \in \{i,j\}$;
(F) If $i\in B_{ind}\cup B_{clq}$, and $s \xrightarrow {\tau } s^{\prime }$ is a transition of $P_i$, then $s \xrightarrow {\tau } s^{\prime }$ is a transition of $P^{\prime }_1$;
(G) If $i\in B_{sng}$, and $s \xrightarrow {\tau } s^{\prime }$ is a transition of $P_i$, then $t \xrightarrow {\tau } t^{\prime }$ is a transition of $P^{\prime }_2$ where $t(i) = s, t^{\prime }(i)=s^{\prime }$ and $t(l) = t^{\prime }(l)$ for $l \not = i$;
(H) If $i\in B_{ind}\cup B_{clq}$ and $\iota _i$ is the initial state of $P_i$ then $\iota ^{\prime }_1 \xrightarrow {\tau } \iota _i$ is a transition of $P^{\prime }_1$.

It is straightforward to check that we can indeed simulate every computation of $\overline{P}^G$ by a corresponding computation of $(P^{\prime }_1,P^{\prime }_2)^{G^{\prime }}$, and vice versa, which takes care of items 1 and 2 in the statement.

For item 3, observe that the communication primitive used by $\overline{P}^{\prime }$ is the same as the one used in $\overline{P}$. $\square$

### Cutoffs and decidability

#### Cutoff

A *cutoff* for ${\textsf {PMCP}}_{\mathcal G}({\mathcal {P}},{\mathcal F})$ is a natural number *c* such that for every $\overline{P}\in {\mathcal {P}}$ and $\varphi \in {\mathcal F}$, the following are equivalent:

(i) $\overline{P}^G\models \varphi$ for all $G\in {\mathcal G}$ with $|V_G| \le c$;
(ii) $\overline{P}^G\models \varphi$ for all $G\in {\mathcal G}$.

Observe that the model checking problem $\overline{P}^G\models \varphi$ (for $\varphi$ an indexed $\textsf {CTL}^*\backslash \textsf {X}$ formula) is decidable since $\overline{P}^G$ is a finite structure and $\varphi$ can be replaced by a Boolean combination of $\textsf {CTL}^*\backslash \textsf {X}$ formulas, e.g., $\exists x. \phi$ becomes $\bigvee _{x \in G} \phi$.

#### Proposition 1

Let ${\mathcal F}$ be a set of indexed-$\textsf {CTL}^*\backslash \textsf {X}$ formulas. If ${\textsf {PMCP}}_{\mathcal G}({\mathcal {P}},{\mathcal F})$ has a cutoff then ${\textsf {PMCP}}_{\mathcal G}({\mathcal {P}},{\mathcal F})$ is decidable.

#### Proof

If *c* is a cutoff, let $G_1, \ldots , G_n$ be all topologies $G$ in ${\mathcal G}$ such that $|V_G| \le c$. The algorithm that solves PMCP takes $\overline{P},\varphi$ as input and checks whether or not $\overline{P}^{G_i} \models \varphi$ for all $1 \le i \le n$. $\square$

We remark that the previous proposition is not constructive. It simply says that from the existence of a cutoff one can deduce the *existence* of an algorithm.

The following theorem says that if there is a cutoff for the set of 1-indexed $\textsf {LTL}\backslash \textsf {X}$ formulas then the set of executions is $\omega$-regular. Although it is stated for pairwise-rendezvous system templates, the proof is generic.

#### Theorem 1

Fix an *r*-ary parameterized topology ${\mathcal G}$, let $\overline{P}$ be a pairwise-rendezvous *r*-ary system template such that the set of atomic propositions $\textsf {AP}_{\textsf {pr}}$ contains all the states of process templates in $\overline{P}$,^9^ and let ${\mathcal F}$ be the set of 1-index $\textsf {LTL}\backslash \textsf {X}$ formulas over $\textsf {AP}_{\textsf {pr}}$. If ${\textsf {PMCP}}_{\mathcal G}(\{\overline{P}\},{\mathcal F})$ has a cutoff, then for every $t \le r$ the set of executions $\textsc {t-exec}_{{\mathcal G}}{(\overline{P})}$ is $\omega$-regular.

#### Proof

Let *c* be a cutoff, and let $\tilde{{\mathcal G}} = \{G\in {\mathcal G}\mid |V_G| \le c\}$ be the set of the topologies in ${\mathcal G}$ below the cutoff. Observe that it is enough to prove that $\textsc {t-exec}_{{\mathcal G}}{(\overline{P})} = \textsc {t-exec}_{\tilde{{\mathcal G}}}{(\overline{P})}$. Indeed, it is not hard to see that given $G\in \tilde{{\mathcal G}}$, and a vertex $v \in V_G$, we can easily modify $\overline{P}^G$ to an NBW that recognizes the language $\textsc {exec}_{{\mathcal G}}(\overline{P},v)$. Thus, by taking the disjoint union of all these NBW for every $G\in \tilde{{\mathcal G}}$ and every vertex $v \in V_G$ of type *t*, we can obtain an NBW accepting $\textsc {t-exec}_{\tilde{{\mathcal G}}}{(\overline{P})}$ (the finiteness of $\tilde{{\mathcal G}}$ ensures that we indeed get a finite state automaton).

We now show that $\textsc {t-exec}_{{\mathcal G}}{(\overline{P})} = \textsc {t-exec}_{\tilde{{\mathcal G}}}{(\overline{P})}$. The fact that $\textsc {t-exec}_{\tilde{{\mathcal G}}}{(\overline{P})} \subseteq \textsc {t-exec}_{{\mathcal G}}{(\overline{P})}$ is obvious. For the other direction, fix some $G\in {\mathcal G}$, and consider some $w \in \textsc {t-exec}_{{\mathcal G}}{(\overline{P})}$. We claim that every prefix *u* of *w* is also the prefix of some word $w^{\prime } \in \textsc {t-exec}_{\tilde{{\mathcal G}}}{(\overline{P})}$. The claim implies that $w \in \textsc {t-exec}_{\tilde{{\mathcal G}}}{(\overline{P})}$ (and thus completes the proof) as follows. For every ${\mathcal G}\in \tilde{{\mathcal G}}$, consider the tree of words in $\textsc {t-exec}_{{\mathcal G}}{(\overline{P})}$ that are prefixes of *w*. Observe that, by the claim above, all (the infinitely many) prefixes of *w* appear in one of these trees, and recall that $\tilde{{\mathcal G}}$ is finite. Hence, by Kőnig’s lemma, there is an infinite path in one of these trees that contains infinitely many such prefixes, and thus all such prefixes. It follows that $w \in \textsc {t-exec}_{\tilde{{\mathcal G}}}{(\overline{P})}$.

It remains to prove the claim. Let $u {:=} u_1 u_2 \ldots u_k$ be a prefix of $w \in \textsc {t-exec}_{{\mathcal G}}{(\overline{P})}$, and let $\psi ^u = \forall x : type(x) = t {.\, \textsf {A}\lnot \phi }$ where

$$\begin{aligned} \phi = (u_1,x) \textsf {U}((u_2,x) \textsf {U}((u_3,x) \textsf {U}(\ldots \textsf {U}(u_k,x)) \ldots ). \end{aligned}$$

Intuitively, this formula says that no *t*-execution starts with *u*. Note that $\psi ^u$ is a 1-indexed $\textsf {LTL}\backslash \textsf {X}$ formula over $\textsf {AP}_{\textsf {pr}}$ (by our assumption on $\textsf {AP}_{\textsf {pr}}$). Observe that since *u* is a prefix of *w*, there exists $G\in {{\mathcal G}}$ such that $\overline{P}^G\not \models \psi ^u$. Thus, since *c* is a cutoff, it is also the case that $\overline{P}^G\not \models \psi ^u$ for some $G\in \tilde{{\mathcal G}}$, i.e., *u* is also a prefix of some word in $\textsc {t-exec}_{\tilde{{\mathcal G}}}{(\overline{P})}$. $\square$

### Two prominent kinds of pairwise rendezvous systems

The parameterized verification literature often distinguishes between two kinds of concurrent systems: those with identical processes, and those in which there is a single process that acts as a controller or environment.

*Identical processes*. Concurrent systems in which all processes are identical are modeled with system arity $r = 1$. In this case there is a single process template *P*, and the topology is $G = (V,E,T_1)$ with $T_1 = V$.

*Identical processes with a controller*. Concurrent systems in which all processes are identical except for one process (typically called a controller, or the environment) can be modeled with system arity $r = 2$, and system templates of the form $(P_1,P_2)$, and we restrict the topologies so that exactly one vertex has type 1 (i.e., runs the controller). We call such topologies *controlled*. We often write (*C*, *U*) instead of $(P_1,P_2)$. We write $\textsc {controller-exec}_{{\mathcal G}}(C,U)$ for the set of executions of the controller process, i.e., $\textsc {1-exec}_{{\mathcal G}}{((C,U))}$. We write $\textsc {user-exec}_{{\mathcal G}}(C,U)$ for the set of executions of the user processes in this 2-ary system, i.e., $\textsc {2-exec}_{{\mathcal G}}{((C,U))}$. When the parameterized topology ${\mathcal G}$ is clear from the context, we omit it.

Let us emphasize that the token passing systems with valueless tokens considered in this work are meaningful only for controlled topologies, since by definition (see Sect. 2.2) there exists exactly one copy of process template $P_1$; this copy is the unique process where the token is at the start of the run.

## Pairwise rendezvous systems

The known decidability results for model checking parameterized pairwise rendezvous systems are for clique topologies and specifications from 1-indexed $\textsf {LTL}\backslash \textsf {X}$ [36]. Thus, we might hope to generalize these results in two directions: more general specification languages and more general topologies. Unfortunately, as the following theorems show, we can not go too far in these directions. In Theorem 2 we show that one can reduce the non-halting problem of two-counter machines (2CMs), known to be undecidable, to the PMCP of 1-indexed $\textsf {CTL}^*_{2}\backslash \textsf {X}$ formulas, i.e., 1-indexed formulas with at most 2 nestings of path quantifiers. Thus, allowing quite limited branching time specifications results in undecidability (already for cliques). This leads to the conclusion that we should restrict the specification logic if we want decidability (e.g., to LTL which has just 1 path quantifier), and try instead to look at topologies other than cliques. Unfortunately, as Theorem 3 shows, we also can not go too far in this direction.

### Theorem 2

${\textsf {PMCP}}_{\mathcal G}({\mathcal {P}},{\mathcal F})$ is undecidable where ${\mathcal F}$ is the set of 1-indexed $\textsf {CTL}^*_{2}\backslash \textsf {X}$ formulas, ${\mathcal G}$ is the set of 1-ary clique parameterized topologies, and ${\mathcal {P}}$ is the set of pairwise-rendezvous 1-ary system templates.

### Proof

We first prove the claim for systems with a controller. Thus, ${\mathcal {P}}$ consists of system templates of the form (*C*, *U*) and ${\mathcal G}$ is the set of 2-ary clique topologies with a single vertex, say vertex 1, that runs the controller *C*.

We reduce the non-halting problem of two-counter machines (2CMs) [46], which is known to be undecidable, to the PMCP. See the preliminaries (Sect. 2) for the definition of 2CM.

**Fig. 1:**
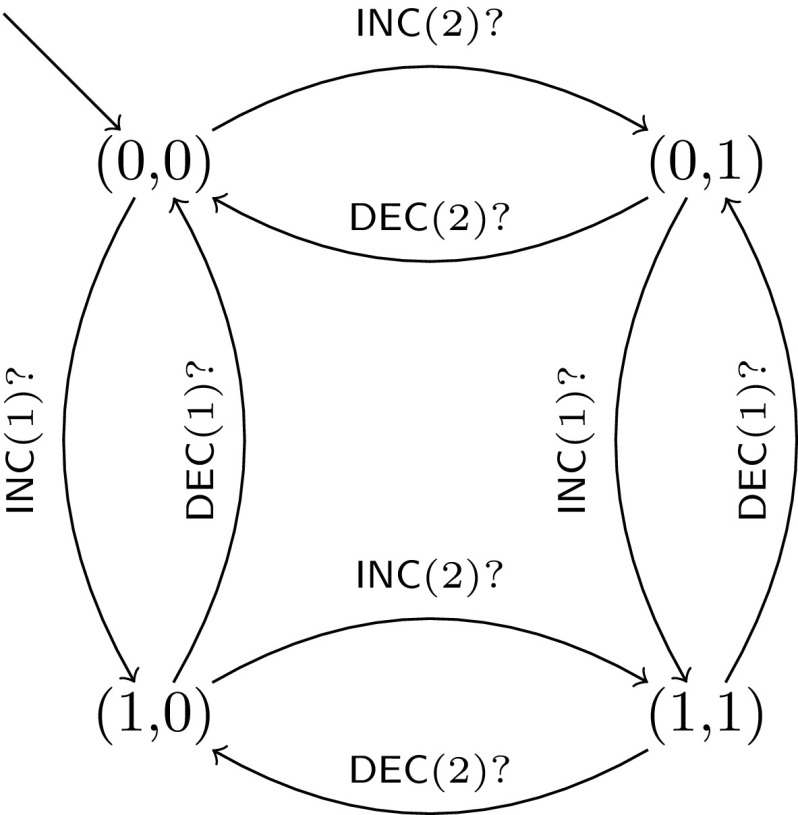
Process template of memory processes for the proof of Theorem 2

The basic idea, based on the reduction in [28], is that the controller simulates the control flow of the 2CM, and the other processes (called *memory processes*) are each holding one bit of each of the two counters, and collectively storing the counter values using a unary encoding (see Fig. 1). The formula $\psi$ to be model-checked is used to specify that the simulated computation of the 2CM halts, as well as to enforce correct simulation of $\mathsf {JZ}$ instructions. Due to the unary encoding we employ, a clique of $n+1$ vertices can simulate the 2CM with counter values up to *n*. Since the 2CM halts if and only if it halts with some bound *n* on the counter values, we can reduce the non-halting problem of the 2CM to the PMCP.

Using pairwise rendezvous, simulating $\mathsf {INC}(i)$ and $\mathsf {DEC}(i)$ is straightforward. For example, $\mathsf {INC}(2)$ can be simulated by a synchronous transition using the message $\mathsf {INC}(2)$, where the process at vertex *v* that is running the controller sends $\mathsf {INC}(2)$ and updates it’s state from $I_j$ to $I_{j+1}$ (simulating the 2CM move to the next instruction), and some vertex *w* running a memory process with a 0-valued counter 2 bit receiving $\mathsf {INC}(2)$ and updating this bit to 1. Simulating a $\mathsf {JZ}$ instruction is slightly more involved since there is no direct way for the controller to query all memory processes for their bit values. However, if all the bits of counter *i* in all memory processes are 0, then none of these processes is in a state with an outgoing local transition labeled by $\mathsf {DEC}(i)?$ and thus, even if the controller is willing to perform a synchronized transition on the message $\mathsf {DEC}(i)$, it will not be able to. In order to make use of this observation, for every instruction of the form $I_j = \mathsf {JZ}(i,k)$, the controller process *C* has the following 3 outgoing transitions: $I_j \xrightarrow {\tau } I_{j+1}, I_j \xrightarrow {\tau } I_k, I_j \xrightarrow {\mathsf {DEC}(i)!} NZ$, where *NZ* is a special sink state labeled by the atomic proposition *nz*. Thus (assuming that for $1 \le l \le m$ we label the state $I_l$ of *C* by the atomic proposition *l*), the formula $\psi _1 = \textsf {G}((j \textsf {U}j+1) \implies \textsf {E}(j \textsf {U}nz))$ specifies that the move from $I_j$ to $I_{j+1}$ is taken only when the counter *i* is not zero, and the formula $\psi _2 = \textsf {G}((j \textsf {U}k) \implies \lnot \textsf {E}(j \textsf {U}nz))$ specifies that the move from $I_j$ to $I_k$ is taken only when counter *i* is zero. The full specification formula is thus $\forall x : type(x) = C {.\, \textsf {A}\left[ \lnot \psi ^{\prime }_1 \vee \lnot \psi ^{\prime }_2 \vee \lnot \textsf {F}(halt,x)\right] }$ where *halt* is an atomic proposition that holds in states corresponding to $\mathsf {HALT}$ instructions and, for $i \in \{1,2\}$, $\psi ^{\prime }_i$ is the formula $\psi _i$ in which every atomic proposition $a \in \textsf {AP}_{\textsf {pr}}$ is replaced by the atomic proposition (*a*, *x*).

We now consider the case of 1-ary clique topology and a single process template *P*, without controller. In this case, *P* is simply the union of the controller and memory process templates *C*, *U* used in the 2-ary clique case above, with an extra initial state $q_0$ that has two outgoing transitions $q_0 \xrightarrow {\tau } I_1, q_0 \xrightarrow {\tau } (0,0)$ one to each of the initial states of *C* and *U*. Thus, each process can nondeterministically decide to play the role of a controller or that of memory. Enforcing that exactly one process plays the controller is done as follows. We introduce a new state $\bot$ labeled by the atomic proposition $\textit{conflict}$, and add the transitions $I_1 \xrightarrow {problem!} \bot , s \xrightarrow {problem?} s$, where *s* ranges over all states of *P* except for (0, 0). Hence, the formula $\psi _3 = 1 \wedge \lnot \textsf {E}(1 \textsf {U}\textit{conflict})$ is satisfied in a computation of the system exactly at a point where one process (playing the controller) is at state $I_1$ (recall that the label 1 holds only in the state $I_1$), and the rest (playing memory) are at state (0, 0). The full formula to be model-checked is thus $\forall x \cdot \lnot \psi ^{\prime }_1 \vee \lnot \psi ^{\prime }_2 \vee \lnot \textsf {F}\psi ^{\prime }_3 \vee \lnot \textsf {F}(halt,x)$ where $\psi ^{\prime }_3$ is $\psi _3$ but with every atom $a \in \textsf {AP}_{\textsf {pr}}$ replaced by the indexed atom $(a,x) \in \textsf {AP}_{\textsf {pr}}\times \textsf {Vars}$. $\square$

Second, pairwise rendezvous systems can easily simulate systems communicating using tokens with values. Thus, the fact that PMCP on token passing systems and parameterized ring topologies and safety property (for the controller) is undecidable (see [28, 52]) implies the same for pairwise rendezvous systems.

### Theorem 3

${\textsf {PMCP}}_{\mathcal G}({\mathcal {P}},{\mathcal F})$ is undecidable where ${\mathcal F}$ is the set of 1-indexed LTL formulas, ${\mathcal G}$ is the 2-ary controlled ring parameterized topology, and ${\mathcal {P}}$ is the set of pairwise-rendezvous 2-ary system templates.

Thus, in the rest of this section, we focus on linear logics and homogeneous parameterized topologies.

### Cutoffs

Unlike systems using disjunctive guards or a valueless token for communication, systems using pairwise rendezvous do not always have a cutoff for 1-index $\textsf {LTL}\backslash \textsf {X}$ formulas.

**Fig. 2:**
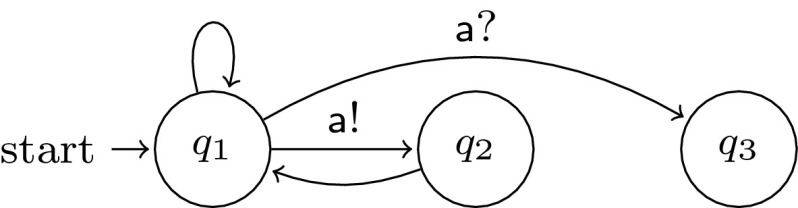
Process template *U*, used to prove Theorem 4

#### Theorem 4

Let ${\mathcal G}$ be the 1-ary controllerless clique parameterized topology, and let ${\mathcal F}$ be the set of 1-index $\textsf {LTL}\backslash \textsf {X}$ formulas. There exists a pairwise-rendezvous process template *U* such that ${\textsf {PMCP}}_{\mathcal G}(\{U\},{\mathcal F})$ has no cutoff.

#### Proof

Define process template $U = (S,R,I,\varPhi )$ as in Fig. 2, where $\varPhi (q_i) = \{q_i\}$. In a system with $n+1$ such processes, one possible behaviour is, up to stuttering, $(q_1 q_2)^n q_1^\omega$. This run does not appear in any system with at most *n* processes. Thus we can define a formula:

$$\begin{aligned} \phi _m = \underbrace{q_1 \wedge (q_1 ~ \textsf {U}~ \cdots ~ \textsf {U}~ (q_1 \wedge (q_1 ~ \textsf {U}~ (q_2 \wedge (q_2 ~ \textsf {U}~ q_1))) \ldots )}_{2m ~ \textit{alternations}}). \end{aligned}$$

Intuitively, $\phi _m$ states that in the system some process (and thus any process) is able to alternatively visit states $q_1$ and $q_2$ *m* times, building the desired prefix $(q_1 q_2)^m q_1$. For a parameterized system with template *U*, every number $c \in \mathbb {N}_0$, fails to be a cutoff, since $\forall n \le c \cdot U^n \not \models \phi _c$ but $U^{c+1} \models \phi _c$. $\square$

Theorem 4 can easily be adapted to controlled topologies by assigning the controller with the same process template *U* as the users:

#### Theorem 5

Let ${\mathcal G}$ be a controlled 2-ary clique parameterized topology and let ${\mathcal F}$ be the set of 1-index $\textsf {LTL}\backslash \textsf {X}$ formulas. There exist pairwise-rendezvous process templates *C* and *U* such that ${\textsf {PMCP}}_{\mathcal G}(\{(C,U)\},{\mathcal F})$ has no cutoff.

### Equivalence to finite-state systems

We first recall the main result in [36, Section 4] (stated as comments after [36, Theorem 4.8]), restated using our notation:

#### Theorem 6

([36]) Let ${\mathcal G}$ be the 1-ary controllerless clique parameterized topology. For every pairwise-rendezvous system template *P* there is an NBW (of linear size and computable in Ptime from *P*) that recognizes the set $\textsc {1-exec}_{{\mathcal G}}{(P)}$.

We now show that a similar result holds for controllerless homogeneous parameterized topologies. Using Lemma 3, we get:

#### Theorem 7

For every *r*-ary controllerless homogeneous parameterized topology ${\mathcal G}$, every pairwise-rendezvous *r*-ary system template $\overline{P}$, and every $i \le r$, there is a linearly sized NBW (computable in Ptime) that recognizes the set $\textsc {i-exec}_{{\mathcal G}}{(\overline{P})}$.

#### Proof

We reduce the case of controllerless homogeneous parameterized topology to 1-ary clique parameterized topology using Lemma 3: given the theorem assumptions, there exists a process template $P^\prime$ and a controllerless clique parameterized topology ${\mathcal G}^\prime$ such that $\textsc {1-exec}_{{\mathcal G}^\prime }{(P^\prime )} = \cup _{i \in [r]} \textsc {i-exec}_{{\mathcal G}}{(\overline{P})}$. By Theorem 6, there is a linearly sized NBW for $\textsc {1-exec}_{{\mathcal G}^\prime }{(P^\prime )}$, and thus, by intersecting it with the constant size NBW that accepts all strings that start with the label of $\iota _i$, we obtain the theorem (we assume without loss of generality—simply by adding new atomic propositions if needed, and then projecting them out of the final NBW—that if $i \ne j$ then $\varPhi _i(\iota _i) \ne \varPhi _j(\iota _j)$). $\square$

Recall that by Theorem 1, if there is a cutoff for the set of 1-indexed $\textsf {LTL}\backslash \textsf {X}$ formulas then the set of executions is $\omega$-regular for any *r*-ary parameterized topology. However, by Theorem 5, 2-ary controlled clique parameterized topologies (and pairwise rendezvous communication) do not always have a cutoff. Furthermore, by constructing an appropriate system template, and using a pumping argument, we are able to show that the set of executions of systems with a controller is not, in general, $\omega$-regular:

#### Theorem 8

Let ${\mathcal G}$ be the 2-ary controlled clique parameterized topology. There exists a pairwise-rendezvous system template (*C*, *U*) for which $\textsc {controller-exec}_{}(C,U)$ is not $\omega$-regular, and a pairwise-rendezvous system template (*C*, *U*) for which $\textsc {user-exec}_{}(C,U)$ is not $\omega$-regular.

#### Proof

**Fig. 3:**
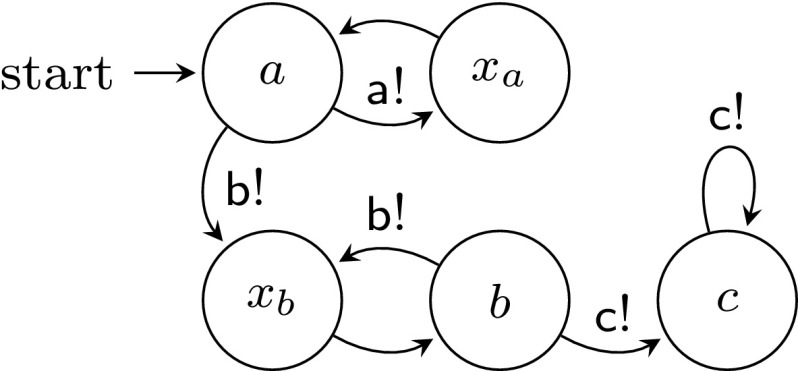
Controller process template

**Fig. 4:**
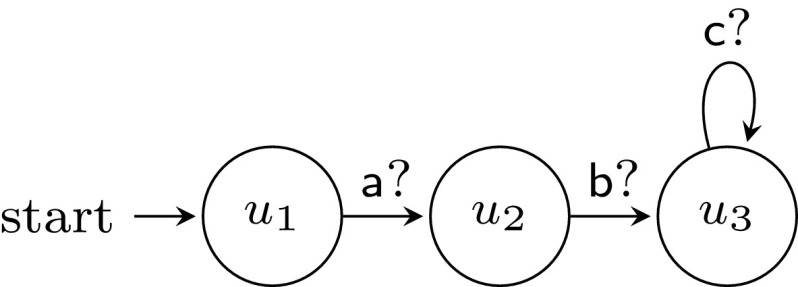
User process template

We first show a system template for which $\textsc {controller-exec}_{}(C,U)$ is not $\omega$-regular. Consider the process templates in Figs. 3 and 4, assuming states are labeled by their names. It is not hard to see that $\textsc {controller-exec}_{}(C,U) = \{a(x_aa)^n (x_bb)^m c^\omega : 0 < m \le n\}$. The following standard pumping argument shows that $\textsc {controller-exec}_{}(C,U)$ is not $\omega$-regular. Assume by way of contradiction that it is, and let $k > 1$ be the number of states of an NBW *A* accepting $\textsc {controller-exec}_{}(C,U)$. Consider an accepting run of *A* on the word $w = a(x_aa)^k (x_bb)^k c^\omega$, and let $q_1, \ldots , q_{k+1}$ be the first $k+1$ states that *A* visits after reading the first *b*. Note that when reaching $q_{k+1}$, the automaton has not read any *c* yet. By the pigeonhole principle, there are $1 \le i < j \le k+1$ such that $q_i = q_j$ and thus, by pumping the loop $q_i \ldots q_j$, one can get accepting runs of *A* on words which are not in $\textsc {controller-exec}_{}(C,U)$, which is a contradiction.

We now show how to obtain $U^{\prime }, C^{\prime }$ such that the language $\textsc {user-exec}_{}(C^{\prime },U^{\prime })$ is not $\omega$-regular. The idea is simply to have the controller switch places with some user process as follows. Have both $C^{\prime }$ and $U^{\prime }$ contain two disjoint copies of *C* and *U* (as defined above), and add new initial states $a^{\prime }, u_1^{\prime }$ to $C^{\prime }$ and $U^{\prime }$ (respectively), with the following transitions: $a^{\prime } \xrightarrow {\mathsf {switch!}} u_1$, $u_1^{\prime } \xrightarrow {\mathsf {switch?}} a$, and $u_1^{\prime } \xrightarrow {\tau } u_1$. Thus, when the new controller sends the message $\mathsf {switch}$, it starts behaving like a *U* process, and the receiving new user process starts behaving like a *C* process. All other user processes can now only take the transition $u_1^{\prime } \xrightarrow {\tau } u_1$, and start behaving like *U* processes. Hence, the language $\textsc {user-exec}_{}(C^{\prime },U^{\prime })$ is the union of $L{:=} u_1^{\prime } \textsc {controller-exec}_{}(C,U)$ and $L^{\prime } {:=} u_1^{\prime } u_1 u_2 u_3^\omega$. Note that $L^{\prime }$ is $\omega$-regular, that $L = \textsc {user-exec}_{}(C^{\prime },U^{\prime }) \cap \lnot L^{\prime }$, and that *L* is not $\omega$-regular. Hence, since $\omega$-regular languages are closed under negations and intersection, it must be that the language $\textsc {user-exec}_{}(C^{\prime },U^{\prime })$ is not $\omega$-regular. $\square$

### Complexity of PMCP

The complexity of PMCP for clique parameterized topologies is studied in [36]:

#### Theorem 9

([36]) Fix an *r*-ary controlled clique parameterized topology ${\mathcal G}$, let ${\mathcal F}$ be the set of 1-index $\textsf {LTL}\backslash \textsf {X}$ formulas, and ${\mathcal {P}}$ the set of pairwise-rendezvous *r*-ary system templates. Then ${\textsf {PMCP}}_{\mathcal G}({\mathcal {P}},{\mathcal F})$ (as well as its program complexity) are Expspace-complete.

Recall that the PMCP program complexity is the complexity when the size of the specification formulas is ignored. The lower bound (for PMCP and its program complexity) follows from the fact that PMCP is Expspace-hard already for clique topologies and the coverability problem [30]. The upper bound for PMCP (and thus also for its program complexity) is proved in [36, Theorem 3.6].

Combing this with Lemma 3 we immediately get:

#### Theorem 10

Fix an *r*-ary controlled homogeneous parameterized topology ${\mathcal G}$, let ${\mathcal F}$ be the set of 1-index $\textsf {LTL}\backslash \textsf {X}$ formulas, and ${\mathcal {P}}$ the set of pairwise-rendezvous *r*-ary system templates. Then ${\textsf {PMCP}}_{\mathcal G}({\mathcal {P}},{\mathcal F})$ (as well as its program complexity) are in Expspace.

**Fig. 5:**
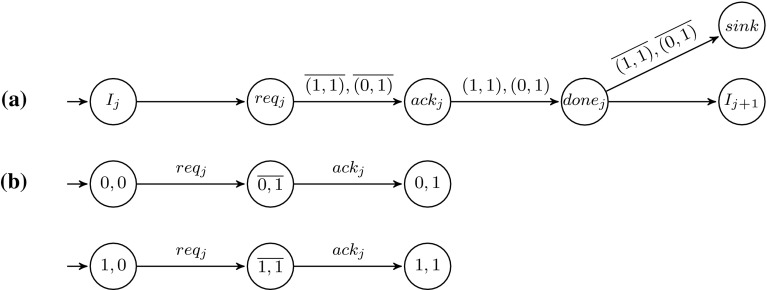
Gadgets used in the proof of Theorem 12

The following theorem shows that for controllerless homogeneous parameterized topologies (i.e., ones with $B_{sng} = \emptyset$) the complexity of PMCP is better.

#### Theorem 11

Fix an *r*-ary controllerless homogeneous parameterized topology ${\mathcal G}$, let ${\mathcal F}$ be the set of 1-index $\textsf {LTL}\backslash \textsf {X}$ formulas, and ${\mathcal {P}}$ the set of pairwise-rendezvous *r*-ary system templates. Then ${\textsf {PMCP}}_{\mathcal G}({\mathcal {P}},{\mathcal F})$ is Pspace-complete, and its program complexity is in Ptime.

#### Proof

The lower bound for the PMCP follows from the fact that $\textsf {LTL}\backslash \textsf {X}$ model checking a single finite state system *P*, with no communication, is Pspace-hard [50]. For the upper bound, take some system template $\overline{P}\in {\mathcal {P}}$ and a specification formula $\psi {:=} Q x : type(x) = t {.\, \phi }$. By Lemma 2, it is enough to check that $\forall \pi \in \textsc {t-exec}_{{\mathcal G}}{(\overline{P})}$ we have that $\pi \models \phi ^{\prime }$. The theorem follows by noting that this can be done by checking for the non-emptiness of the product of an NBW for $\lnot \phi ^{\prime }$ (obtained using [53]), and the NBW for $\textsc {t-exec}_{{\mathcal G}}{(\overline{P})}$ (obtained from Theorem 7). $\square$

## Disjunctive guards

In this section we consider disjunctively-guarded systems (see Sect. 2.2) arranged in parameterized homogeneous topologies.

It is known that disjunctively-guarded systems are strictly less expressive than pairwise rendezvous ones [7]. Nonetheless, the following theorem states that also for disjunctive guards the PMCP of 1-indexed $\textsf {CTL}^*_{2}\backslash \textsf {X}$ formulas is undecidable. The proof uses a reduction from the non-halting problem of 2CMs, and follows a similar line to the one used to prove Theorem 2. The main complication here is that, unlike the case of pairwise rendezvous, mutual exclusion is not easily obtainable using disjunctive guards, and thus more complicated gadgets are needed to ensure that the counter operations are simulated correctly.

### Theorem 12

${\textsf {PMCP}}_{\mathcal G}({\mathcal {P}},{\mathcal F})$ is undecidable where ${\mathcal F}$ is the set of 1-indexed $\textsf {CTL}^*_{2}\backslash \textsf {X}$ formulas, ${\mathcal G}$ is the 1-ary clique parameterized topology, and ${\mathcal {P}}$ is the set of disjunctively-guarded 1-ary system templates.

### Proof

We adapt the proof of the similar statement (Theorem 2) for pairwise rendezvous. Recall that the proof of that theorem proceeded by building, given a 2CM, a process template *P* and a specification formula $\psi$, such that the 2CM does not halt iff $\overline{P}^G\models \psi$ for every 1-ary clique $G$. Here, we show how to adapt the process template *P* from that proof to use disjunctive guards instead of pairwise rendezvous (consequently, $\psi$ also changes).

At first glance, it may look like all we have to do is replace every transition of the form $s \xrightarrow {\mathsf {a!}} s^{\prime }$ (resp. $s \xrightarrow {\mathsf {a?}} s^{\prime }$) with the transition $s \xrightarrow {g} s^{\prime }$, where the guard *g* is the set of all states that have an outgoing transition labeled by $\mathsf {a?}$ (resp. $\mathsf {a!}$). Unfortunately, this introduces the following problem: once the controller is in a state $I_j$, associated with an increment or a decrement instruction, it opens the gate for *any number* of memory components to update their bits, instead of just one. Hence, we have to work harder.

What we do is to replace every transition of the form $s \xrightarrow {\mathsf {a!}} s^{\prime }$ (resp. $s \xrightarrow {\mathsf {a?}} s^{\prime }$) in the controller (resp. memory) component of *P*, with a series of guarded moves, for every $a \in \{\mathsf {INC}(1), \mathsf {DEC}(1), \mathsf {INC}(2), \mathsf {DEC}(2)\}$. The key point is that if more than one memory component enters the second stage of such a bit update sequence then it opens up the possibility for a computation segment that is impossible if only one memory component enters this stage, and the presence of such a segment can be detected by the specification formula.

We illustrate these sequences by considering the representative case of $\mathsf {INC}(2)$. Figure 5a shows the corresponding gadget used by the controller component, i.e., the sequence replacing a transition $(I_j \xrightarrow {\mathsf {\mathsf {INC}(2)}!} I_{j+1}$); and Fig. 5b shows the corresponding gadgets—one per *j* for which $I_j$ is an $\mathsf {INC}(2)$ instruction—used by the memory component, i.e., the sequences replacing the transitions $(0,0) \xrightarrow {\mathsf {\mathsf {INC}(2)}?} (0,1)$, and $(1,0) \xrightarrow {\mathsf {\mathsf {INC}(2)}?} (1,1)$. The formula that guarantees that instruction $I_j$ is simulated by incrementing at most one bit of counter 2 is $\phi _j {:=} G(ack_j \implies \lnot E (ack_j U (done_j U sink)))$. Similar gadgets and formulas are used for the other increment and decrement transitions. Finally, we replace a transition $I_j \xrightarrow {\mathsf {INC}(1)!} NZ$ with the transition $I_j \xrightarrow {\{(1,0), (1,1)\}} NZ$, and the transition $I_j \xrightarrow {\mathsf {INC}(2)!} NZ$ with the transition $I_j \xrightarrow {\{(0,1), (1,1)\}} NZ$ (recall that these transitions are used, for a $\mathsf {JZ}$ instruction $I_j$, to test if a given counter is indeed not zero). We conclude by modifying the specification formula $\psi$, used in the proof of Theorem 2, as follows. First, we update it to reflect the fact that we now use sequences of transitions to get from a state $I_j$ to $I_{j+1}$ in the controller; e.g., we replace $j\ \textsf {U}\ j+1$ with $j\ \textsf {U}\ req_j\ \textsf {U}\ ack_j\ \textsf {U}\ done_j\ \textsf {U}\ j+1$. Second, we make it disjunctive with $\bigvee _{j=1}^m \lnot \phi _j$ (where *m* is the number of instructions of the 2CM) $\square$

We conclude that we should restrict the specification logic if we want decidability, and in the rest of this section we focus on 1-indexed $\textsf {LTL}\backslash \textsf {X}$. The following theorem shows that we also have to limit the topologies.

### Theorem 13

${\textsf {PMCP}}_{\mathcal G}({\mathcal {P}},{\mathcal F})$ is undecidable where ${\mathcal F}$ is the set of 1-indexed $\textsf {LTL}\backslash \textsf {X}$ formulas, ${\mathcal G}$ is the controlled ring parameterized topology, and ${\mathcal {P}}$ is the set of disjunctively-guarded 2-ary system templates.

### Proof

As before, we give a reduction from the non-halting problem of 2CM. It will be useful later to assume w.l.o.g. that the 2CM contains no self-referring goto (i.e., an instruction of the form $I_j = \mathsf {JZ}(i, j)$). Given a 2CM, with instructions $I_1, \ldots I_m$, we build a system template $\overline{P}= (C,U)$, where *C* is a controller process template and *U* is a user process template. The basic encoding is as in the previous undecidability results—the controller simulates the control of the 2CM, and each user process stores one unary bit for each counter. We augment this basic encoding by also storing the currently simulated instruction, as well as a status flag, in each user process. For technical convenience, we also have the constant flag $\mathsf {false}$ in each controller state. More formally, each state of *U* is of the form $(j, f, c_1, c_2)$, where $j \in \{0, \ldots m\}$ is a number of an instruction of the 2CM (we consider $I_0$ to be a dummy instruction); $f \in \{\mathsf {true}, \mathsf {false}\}$ is a flag indicating the status of the currently simulated instruction, and will be explained later; and $c_1, c_2 \in \{0,1\}$ are the bits of the counters 1 and 2, respectively. The states of *C* are of the form (*j*, *f*), with the same meaning as in *U*, except that *f* is always false (i.e., the set of states is $\{0, \ldots m\} \times \{\mathsf {false}\}$). The initial state of *C* is $(0,\mathsf {false})$, and of *U* is $(0,\mathsf {false}, 0, 0)$.

The transitions in *U* and *C* are as follows. For every $i \in \{0, \ldots , m\}$, and $x \in \{\mathsf {true}, \mathsf {false}\}$, let $Y_{i,x}$ be the set of all states in *C* and *U* with the instruction number *i* and the flag *x*. For every such $Y_{i,x}$, there is a transition $(j, \mathsf {false}) \xrightarrow {Y_{i,x}} (j^{\prime }, \mathsf {false})$ in *C* iff $j = i$, and $I_{j^{\prime }}$ is the instruction that should execute after $I_j$, i.e., if $i=0$ then $j^{\prime }=1$; if $I_i$ is of the form $\mathsf {JZ}(h,k)$ then if $x = \mathsf {false}$ (indicating that counter *i* is zero) then $j^{\prime } = k$, and otherwise $j^{\prime } = i+1$; and if $i > 0$ and $I_i$ is not a $\mathsf {JZ}$ instruction and $x =\mathsf {true}$ then $j^{\prime } = i+1$, however, if $x = \mathsf {false}$ (indicating that the instruction could not be simulated, i.e., it was an increment and all bits were already 1) then there is no transition. In *U*, there is a transition $(j, f, c_1, c_2) \xrightarrow {Y_{i,x}} (j^{\prime }, f^{\prime }, c_1^{\prime }, c_2^{\prime })$ iff $j \ne i$, $j^{\prime } = i$ and *(i):* if $x = \mathsf {true}$ then $f^{\prime }=\mathsf {true}$, $c_1^{\prime } = c_1$, and $c_2^{\prime } = c_2$; *(ii):* if $x = \mathsf {false}$ then if the instruction $I_i$ can not be simulated by updating the bits $c_1$ or $c_2$ (e.g., $I_i = \mathsf {INC}(1)$ and $c_1=1$) then $f^{\prime }=\mathsf {false}$, $c_1^{\prime } = c_1$, and $c_2^{\prime } = c_2$, and otherwise $f^{\prime }=\mathsf {true}$, and the relevant counter bit is updated according to $I_i$ (e.g., $I_i = \mathsf {INC}(1)$, $c_1=0$, $c_1^{\prime }=1$ and $c_2^{\prime } = c_2$). Note that if the instruction $I_i$ is of the form $\mathsf {JZ}(h,k)$, then in case (ii) above we have that $c_1^{\prime } = c_1, c_2^{\prime } = c_2$; and if $c_j =1$ then $f^{\prime } = \mathsf {true}$, and otherwise $f^{\prime }= \mathsf {false}$.

We now describe how the simulation works. Let $G\in {\mathcal G}$ be a unidirectional ring with vertices $v_0, \ldots v_n$ arranged in a clockwise fashion, with edges going anti-clockwise (i.e., from $v_i$ to $v_{i-1}$), and assume that $v_0$ is the controller. The simulation of each 2CM instruction takes one “round” with *n* steps, where at each step $0 \le i \le n$ process $v_i$ moves. Observe that (by the structure of *C*) $v_0$ can only move when its neighbour $v_n$ has the *same* instruction number as it does, and a user process $v_i$ can only move when $v_{i-1}$ has a *different* instruction number than it does. Thus, the simulation can only proceed in this rounds’ structure since the following two invariants are maintained: *(i)* at the beginning of each round all processes have the same instruction number *j* and; *(ii)* at the end of every step $0 \le i \le n$ inside a round, processes $v_0, \ldots , v_i$ store the currently simulated instruction, and processes $v_{i+1}, \ldots , v_n$ store the previously simulated instruction. Recall that by our assumption, that the 2CM never jumps from an instruction $I_j$ back to itself, these two instruction numbers are always different.

A single round simulating an instruction $I_j$ begins by $v_0$ moving to the state $(j, \mathsf {false})$; then, at each step $1 \le i \le n$, the user process $v_i$ “looks” at the state of $v_{i-1}$, copies the instruction number *j*, and proceeds according to the flag *f* stored in $v_{i-1}$: if $f =\mathsf {false}$ it means that the counter operation was not yet simulated (or in the case of a $\mathsf {JZ}$ instruction, all the relevant bits in $v_1, \ldots , v_{i-1}$ were 0), whereas if $f =\mathsf {true}$ then the operation was simulated (or in the case of a $\mathsf {JZ}$, one of the previous bits was 1). Thus, in the first case $v_i$ tries to simulate the instruction if it can (e.g., if $I_j = \mathsf {DEC}(2)$ and $c_2=1$ then it changes $c_2$ to 0), and sets its flag to $\mathsf {true}$ if it succeeded, and to $\mathsf {false}$ otherwise; whereas in the second case it simply sets its flag to $\mathsf {true}$ (without changing the counters’ bits) to propagate the information that the Instruction is done/resolved. At the end of the round, the value of the flag *f* of $v_m$ holds the information needed for the controller to move at the beginning of the next round to the correct succeeding instruction. Namely, if $f = \mathsf {true}$ then it means that an increment or a decrement was successful, or that a $\mathsf {JZ}$ should not jump since the counter is not zero; and if $f = \mathsf {false}$ then it means that an increment command was not simulated since all the counter’s bits are already 1 (this deadlocks the simulation), or that a $\mathsf {JZ}$ should jump since the counter is zero.

Let $\psi {:=} \forall x : type(x) = C {.\, \textsf {A}\lnot \textsf {F}\ halt}$, where *halt* is an atomic proposition that holds in states corresponding to $\mathsf {HALT}$ instructions. Given $G\in {\mathcal G}$ of size *n*, it is not hard to see from the description above that $\overline{P}^G$ is deterministic, and that it simulates the run of the 2CM as long as both counters stay below *n*. Thus, the 2CM does not halt iff $\psi$ holds for all $G\in {\mathcal G}$. $\square$

It is worth noting that the proof above can be easily modified to use 1-ary topologies and process templates as long as the symmetry of the rings is broken somehow (e.g., using spoked wheels instead of rings), thus allowing a virtual controller to be designated. We conclude that we should restrict the topologies if we want decidability, and in the rest of this section we focus on homogeneous topologies.

### Cutoffs

By [24], for the *r*-ary clique parameterized topology and universal 2-indexed $\textsf {LTL}\backslash \textsf {X}$ formulas, there is a cutoff of size $|U|+2$ (where *U* is the process template). The following theorem extends this to homogeneous topologies (for the case of 1-indexed $\textsf {LTL}\backslash \textsf {X}$). Our proof (even when restricted to clique topologies) uses different and simpler reasoning than [24]. Note that Theorem 13 implies that an extension to general parameterized topologies is not possible.

First we need the following definitions. Given an *r*-ary parameterized homogeneous topology ${\mathcal G}$, generated by an *r*-ary topology $H = (V_H,E_H,\bar{T})$ with $B_{sng}$, $B_{clq}$, $B_{ind}$, let the *controller types* $I^C{:=} \{i \in [r] \mid type^{-1}(i) \in B_{sng}\}$ be the types in [*r*] that are associated with a singleton, and let the *user types* $I^U {:=} [r] {\setminus } I^C$ be the types associated with a clique or an independent set.

#### Theorem 14

Let ${\mathcal F}$ be the set of 1-index $\textsf {LTL}\backslash \textsf {X}$ formulas, $\overline{P}$ be a disjunctively-guarded *r*-ary system template, and ${\mathcal G}$ be an *r*-ary homogeneous parameterized topology. Then the expression $2 + |I^C| + \varSigma _{i \in I^U} |S_i|$, where $S_i$ are the states of $P_i$, is a cutoff for ${\textsf {PMCP}}_{\mathcal G}(\{\overline{P}\},{\mathcal F})$.

#### Proof

Assume w.l.o.g. (by renaming states and updating the guards on the transitions to match) that $i \ne j \implies S_i \cap S_j = \emptyset$. Let $S^U {:=} \cup _{i \in I^U} S_i$ be the set of all *user states*. Let ${\mathcal G}$ be generated by an *r*-ary topology $H = (V_H,E_H,\bar{T})$ with $B_{sng},B_{clq}, B_{ind}$. The cutoff number is $c = 2 + |I^C| + |S^U|$. Let ${\mathcal G}^{\prime }$ be the set of topologies of ${\mathcal G}$ with at most *c* vertices. In the following we will show that any trace *w* of a system with more than *c* vertices is also present in a system with *c* vertices. Formally: for every $t \in [r]$, $G\in {\mathcal G}$, and $w \in \textsc {t-exec}_{G}{(\overline{P})}$, we show that $w \in \textsc {t-exec}_{G^{\prime }}{(\overline{P})}$, for some topology $G^{\prime } \in {\mathcal G}^{\prime }$; and thus that $\textsc {t-exec}_{{\mathcal G}}{(\overline{P})} = \textsc {t-exec}_{{\mathcal G}^{\prime }}{(\overline{P})}$. Thus, given a 1-indexed formula $\phi$, by using Lemma 2 we get: $\forall G\in {\mathcal G}.\ \overline{P}^G\models \phi$ iff $\forall \pi \in \textsc {t-exec}_{{\mathcal G}}{(\overline{P})}.\ \pi \models \phi ^{\prime }$ iff $\forall \pi \in \textsc {t-exec}_{{\mathcal G}^{\prime }}{(\overline{P})}.\ \pi \models \phi ^{\prime }$ iff $\forall G\in {\mathcal G}^{\prime }.\ \overline{P}^G\models \phi$. This allows us to conclude that *c* is a cutoff.

Assume any $t \in [r], G\in {\mathcal G}$, and $w \in \textsc {t-exec}_{G}{(\overline{P})}$. Let $\pi$ be a state-labelled run of $\overline{P}^G$ such that $w = \mathsf {destutter}(proj_v(\pi ))$ for some $v \in V_G$, and let $S^U_\pi$ be the states in $S^U$ that are visited along $\pi$. We construct a run $\pi ^{\prime }$ in $\overline{P}^{G^{\prime }}$, of a suitably sized $G^{\prime }$, that induces *w*. The intuition is that $G^{\prime }$ simulates every controller process (i.e., a process associated with a vertex in $B_{sng}$) exactly; for every $s \in S^U_\pi$ it uses one process to reach the guard *s* and keep it open forever; and two more processes: a process *x* of type *t* whose moves will induce *w* and, in case that *x* can induce *w* only by moving finitely many times, another process *y* that moves infinitely often on $\pi$ (to ensure that $\pi ^{\prime }$ is infinite, and thus a run).

Consider the function ${ first}: S^U_\pi \mapsto V_G$ that maps a state *s* to a process that has visited *s* first in $\pi$, i.e., ${ first}(s) {:=} v$ where *v* is such that there is some $j \le |\pi |$ with $\pi _j(v) = s$, and in the prefix $\pi _1, \ldots \pi _{j-1}$ no process is in state *s*. Note that ${ first}(s)$ is uniquely determined except if *s* is an initial state of a process template (in which case it may be that *first*(*s*) can be chosen in more than one way). We first enlarge the topology $G$ to obtain a topology $\hat{G}$ as follows: for every $s \in S^U$, we add a process $P^s$ to the clique or independent set containing ${ first}(s)$. We say that $P^s$ is a *companion* of the process ${ first}(s)$. Observe that a process *j* may have zero, one, or many companion processes, and let *J* be the set of processes with at least one companion.

Let $\hat{\pi }$ be a run of $\overline{P}^{\hat{G}}$ that is obtained from $\pi$ by augmenting every move of a process $j \in J$ with a sequence of identical moves of its companion processes, with the restriction that when a process $P^s$ reaches the state *s* it stays there forever. More formally, we begin by designating all companion processes as *active*. We then consider the transitions of $\pi$ in order; a transition in which a process $j \in J$ changes states (by taking some internal or synchronizing local transition $p \xrightarrow {\alpha } q$) is replaced with the following sequence of transitions: if $P^q$ is an active companion of *j* then have $P^q$ take this local transition and designate it as *inactive*, then have all the remaining active companions of *j*, as well as *j*, take this transition (in some arbitrary order). It is easy to see that $\hat{\pi }$ is a run of $\overline{P}^{\hat{G}}$. Indeed, since all the companions of *j* are in the same clique (or independent set) as *j*, they have exactly the same neighbours as *j*, and thus see the same open guards—allowing them to mimic *j*. Furthermore, for every $v \in V_G$ we have that $\mathsf {destutter}(proj_v(\pi )) = \mathsf {destutter}(proj_v(\hat{\pi }))$, since the process at *v* made exactly the same moves in both runs—only sometimes waiting longer between moves in $\hat{\pi }$. Observe that (by the definition of *first* and by the construction of $\hat{\pi }$), for every state $s \in S^U$, the first process on $\hat{\pi }$ to visit *s* is the companion process $P^s$, and that once $P^s$ reaches *s* it never leaves. It follows that if we take any two processes *x*, *y* in $V_G$ (such that *y* moves infinitely many times on $\pi$), together with all the controller processes and all the companion processes, we can obtain—by simply deleting all transitions in $\hat{\pi }$ that involve the other processes—a run $\pi ^{\prime }$ in a system $G^{\prime }$ of size at most $2 + |I^C| + |S^U_\pi |$ in which $\mathsf {destutter}(proj_x(\pi ^{\prime })) = \mathsf {destutter}(proj_x(\hat{\pi }))$. The theorem follows by recalling that $\mathsf {destutter}(proj_x(\pi )) = \mathsf {destutter}(proj_x(\hat{\pi }))$ for all $x \in V_G$, and that $\mathsf {destutter}(proj_x(\pi )) = w$ for some $x \in V_G$. $\square$

The following theorem shows that the cutoff in Theorem 14 is tight for controllerless cliques, up to an additive constant. A similar result has been shown in a slightly more complex scenario (viz. controlled clique topologies with 2-indexed $\textsf {LTL}\backslash \textsf {X}$) [10].

#### Theorem 15

Let ${\mathcal G}$ be the 1-ary controllerless clique parameterized topology, let ${\mathcal F}$ be the set of 1-index $\textsf {LTL}\backslash \textsf {X}$ formulas, and let $d > 0$. There is a disjunctively-guarded system template *P* of size *d* such that $d+1$ is the smallest cutoff for ${\textsf {PMCP}}_{\mathcal G}(\{P\},{\mathcal F})$.

#### Proof

**Fig. 6:**
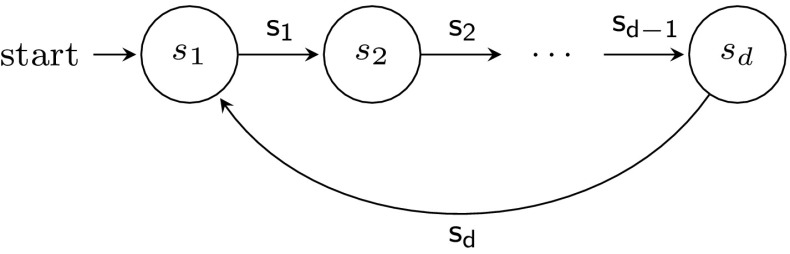
Process $U=(S_U,R_U,I_U,\varPhi _U)$ used to prove Theorem 15

Consider the process template depicted in Fig. 6, where $\varPhi _U(s_i) = \{s_i\}$. It is easy to see, by induction on *i*, that if a process can take the local transition guarded by $s_i$ then there must be at least one process in each state $s_j$ for all $j \le i$. Hence, for a process to take the transition from $s_d$ to $s_1$ there must be at least $d+1$ processes in the system. It follows that the formula $\phi _d = \forall x \cdot AG((s_d,x) \rightarrow G (s_d,x))$ holds in all systems with at most *d* processes, but not in a system with more than *d* processes. $\square$

### Equivalence to finite-state systems

There are several techniques for solving the PMCP for 1-indexed $\textsf {LTL}\backslash \textsf {X}$ formulas for systems using disjunctive guards. One of these is the automata theoretic approach. The main ingredient we need in order to apply this approach is to find an NBW that accepts the set of all possible executions of the system, for any number of copies of user processes *U*. We begin by showing that, in general, such an automaton is necessarily big, i.e., exponential in the size of the process templates. We show this by suitably encoding the language of palindromes.

#### Theorem 16

Let ${\mathcal G}$ be the 2-ary controlled clique parameterized topology. For every $l > 0$, there exist a disjunctively-guarded system template (*C*, *U*), where the sizes of *C* and *U* are $\varTheta (l)$, such that the smallest NBW whose language is $\textsc {controller-exec}_{}(C,U)$ has size at least $2^{\varOmega (l)}$.

#### Proof

**Fig. 7:**
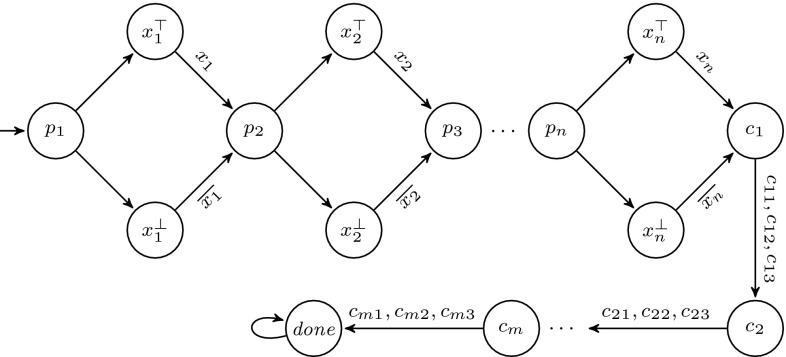
Process C in the proofs of Theorems 16 and 20

**Fig. 8:**
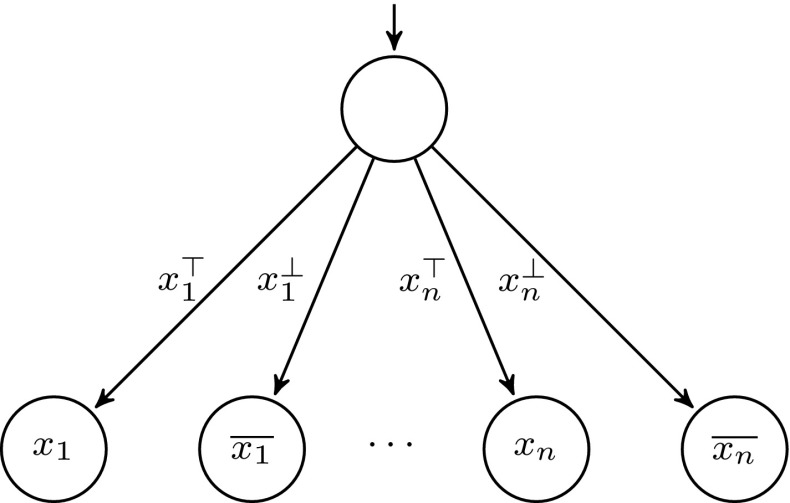
Process U in the proofs of Theorems 16 and 20

Fix $l \in \mathbb {N}$, and consider the Boolean formula $\phi _l {:=} \bigwedge _{i \in [l]} [x_i \iff x_{2l-i+1}]$. Observe that $\phi _l$ is equivalent to $\bigwedge _{i \in [l]} \left[ (\lnot x_i \vee x_{2l-i+1}) \wedge (x_i \vee \lnot x_{2l-i+1})\right]$, and let *C*, *U* be defined as in Figs. 7 and 8 (see also the description in the proof of Theorem 20) with respect to $\phi _l$. Note that the sizes of *C* and *U* are linear in *l*. Let *L* be the language of finite words of the form $p_1 a_1 p_2 a_2 \ldots p_{2l} a_{2l}$ where each $a_i \in \{x_i, \overline{x_i}\}$ ($1 \le i \le 2l$), and $a_i = x_i$ if and only if $a_{2l-i+1} = x_{2l-i+1}$ ($1 \le i \le l$).

Observe that $\textsc {controller-exec}_{}(C,U)$ is exactly the $\omega$-regular language $L \cdot c_1c_2\ldots c_m \cdot (done)^\omega$. By projecting out the letters $p_1, \ldots , p_n, c_1, \ldots , c_m$, and replacing all transitions on $x_1, \ldots , x_n$ with transitions on 0, and all transitions on $\overline{x_1}, \ldots , \overline{x_n}$ with transitions on 1, one obtains (with no blowup) a nondeterministic finite automaton for the language of palindromes in the set $\{0,1\}^{2l}$. It is well known that every NFW for this language requires at least $2^l$ states [37, Theorem 1, Example 2], thus concluding the proof. $\square$

Given $\overline{P}= (C,U)$, the proof in [24] of a $|U|+2$ cutoff for 1-indexed $\textsf {LTL}\backslash \textsf {X}$ actually shows the following stronger result: the set $\textsc {controller-exec}_{}(C,U)$, of controller executions of controlled cliques of all sizes, is equal to the set of controller executions of $\overline{P}^G$, where $G$ is a clique of size $|U|+2$. Observe that it is easy to modify $\overline{P}^G$ to obtain, with no blowup, an NBW accepting its set of controller executions. Thus, we get that $\textsc {controller-exec}_{}(C,U)$ is recognizable by an NBW of size $|C| \times {|U|}^{\varOmega (|U|)}$. Since (by Theorem 16) this cutoff is tight, there is no hope of obtaining a smaller NBW using this technique.

In the following we prove that, surprisingly, disjunctively-guarded systems in homogeneous parameterized topologies can be model checked using a smaller NBW, of size roughly $O({N_C}^2 \times 2^{N_U})$, where $N_C$ is the product of the sizes of controller process templates and $N_U$ is the sum of the sizes of all the user process templates. This result is given in two steps, first showing that this property holds for controlled clique parameterized topologies, and next generalizing it to the case of homogeneous parameterized topologies.

#### Theorem 17

Let ${\mathcal G}$ be the 2-ary controlled clique parameterized topology. For every disjunctively-guarded system template (*C*, *U*) there is an NBW *K*(*C*, *U*) of size $O(|C|^2 \times 2^{|U|})$ recognizing $\textsc {controller-exec}_{}(C,U)$. The same is true for $\textsc {user-exec}_{}(C,U)$.

Before we prove the theorem we note that it is sufficient to prove the theorem for $\textsc {controller-exec}_{}(C,U)$. Indeed, reduce the case of $\textsc {user-exec}_{}(C,U)$ by forming a new controller $C^{\prime }$ that simulates *C* and *U* using a product construction. Furthermore, an automaton for $\textsc {user-exec}_{}(C,U)$ can be obtained with a linear blowup from an automaton for $\textsc {controller-exec}_{}(C^{\prime },U)$ by projecting on the user component of $C^{\prime }$ and destuttering. Thus, in the following we focus on $\textsc {controller-exec}_{}(C,U)$.

We now give the intuition of the proof. Given *C*, *U* we build a transition system ${T}$ with states of the form $(c, Y) \in S_C \times 2^{S_U}$. The idea is that ${T}$ simulates *C*, and records in *Y* all the user states that could have been reached in a finite number of steps in systems of arbitrary size. We identify certain states (*c*, *Y*) of ${T}$ as good, i.e., either, in *C* there is a self loop from *c* to *c* with a guard in *Y*, or in *U* there is a cycle with guards from $Y \cup \{c\}$. We define a run of ${T}$ as good if it is not eventually constant or from some point on it only sees good states. The idea is that a run that is ultimately constant, say with state (*c*, *Y*), is called good if the controller stays in *c* forever. We prove that $\alpha \in \textsc {controller-exec}_{}(C,U)$ if and only if $\alpha$ is the destuttering of a good run of ${T}$.

We now give the proof.

#### Proof of Theorem 17

Fix $C = (S_C, R_C, \{\iota _C\}, \varPhi _C)$ and $U = (S_U, R_U, \{\iota _U\}, \varPhi _U)$. For non-empty $Y \subseteq S_U$ and $c \in S_C$ define

$$\begin{aligned} next(c,Y) := \{s \in S_U \, \vert \,\exists y \in Y, \exists \gamma \in Y \cup \{c,\tau \}, y \xrightarrow {\gamma } s\}, \end{aligned}$$

and define *Reach*(*c*, *Y*) inductively:

- $$Reach_1(c,Y) := Y$$,
- $$Reach_{n+1}(c,Y) := Reach_n(c,Y) ~ \cup ~$$
$$next(c,Reach_n(c,Y))$$, and
- $$Reach(c,Y) := \bigcup _{i \ge 1} Reach_i(c,Y)$$.

Observe that $Reach_i(c,Y)$ is non-decreasing in *i*, and contained in $S_U$. Thus for every *Y*, *c* there exists $k \le |S_U|$ such that $Reach(c,Y) = Reach_k(c,Y)$.

#### Lemma 4

Fix non-empty $Y \subseteq S_U$ and $c \in S_C$. For all $L \in \mathbb {N}$, and every configuration $(c,\bar{u})$ for which every $y \in Y$ appears in $\bar{u}$ at least $L |S_U|$ times, there is a finite path of a DG system starting with configuration $(c,\bar{u})$ such that the first co-ordinate of every configuration in the path is *c*, and the path ends with some configuration $(c,\bar{v})$ such that every $y^{\prime } \in Reach(c,Y)$ appears in $\bar{v}$ at least *L* times.

#### Proof of Lemma

Fix *Y*, *c*, *L* and $\bar{u}$ so that every $y \in Y$ appears in $\bar{u}$ at least $L|S_U|$ times. Say $Reach(c,Y) {\setminus } Y = \{s_1, \ldots , s_K\}$ and order this set according to the earliest stage in which an element appears in this set in the construction of *Reach*(*c*, *Y*), say $s_1< s_2< \ldots < s_K$ (in case that more than one element appears in a given stage, break ties arbitrarily). Build a state-labeled path $(c,\bar{u}_0) (c,\bar{u}_1) \ldots$ starting with $\bar{u}_0 {:=} \bar{u}$ such that, for each $s_i$ in turn, moves *L* many processes from a state in *Y* into state $s_i$. Note that this can be done because: (i) for each $s_i$ there exists a state *y* in *Y* and a sequence of enabled moves from *y* to $s_i$ (indeed, in this construction, once a guard is enabled it is never disabled), and (ii) there are enough processes in *y* (indeed, each $s_i$ uses *L* processes from *y*, and there are at most $|S_U|-1$ many $s_i$ to take care of, thus we are safe if initially there are at least $L (|S_U|-1)$ processes in state *y*). Also note that at the end of this process, for every $y \in Reach(c,Y)$ there are at least *L* processes in state *y* (indeed, if $y \in Y$ then at least *L* processes in *Y* did not move, and if $y \in Reach(c,Y) {\setminus } Y$ then the construction moved at least *L* processes into state *y*). This completes the proof of the Lemma. $\square$

Define a transition system ${T}= (S_{T}, R_{T}, I_{T},\varSigma _{T})$ whose labels are the states of the controller, as follows:

- $S_{T}{:=} (S_C \times 2^{S_U}) \cup \{\iota \}$ where $\iota$ is a new symbol,
- $$I_{T}= \{\iota \}$$,
- $$\varSigma _{T}= S_C$$,
- $$R_{T}= R^{\textsc {Loop}} \cup R^{\textsc {Prog}} \cup R^{\textsc {Init}}$$,

where

- $R^{\textsc {Loop}}$ consists of transitions $(c,Y) \xrightarrow { c} (c,Y)$ where $(c,Y) \in S_{T}$;
- $R^{\textsc {Prog}}$ consists of transitions $(c,Y) \xrightarrow { c^\prime } (c^\prime ,Y^\prime )$ for which $Y^\prime = \textit{Reach}(c^\prime , Y)$ and there exists $\gamma \in Y \cup \{\tau \}$ such that $(c,\gamma ,c^\prime ) \in R_C$;
- $$R^{\textsc {Init}} = \{\iota \xrightarrow {\iota _C} \textit{Reach}(\iota _C, \{\iota _U\})\}$$.

#### Definition 1

A state (*c*, *Y*) of ${T}$ is called $\textsc {good}$ iff it satisfies the following property: if there is no $\gamma \in Y \cup \{\tau \}$ such that $(c,\gamma ,c) \in R_C$, then there exists a cycle in *U*, starting and ending in an element of *Y*, and each transition of it is guarded by an element of $Y \cup \{c,\tau \}$.

An infinite word *w* is *eventually constant* if there exists $i \in \mathbb {N}$ such that $w_i = w_j$ for all $j \ge i$. In this case, we can call $w_i$ the *constant symbol* in *w*. A run $s_0a_0s_1a_1 \ldots$ of ${T}$ is good if the state-labeled run $s_0s_1 \ldots$ satisfies the following property: if it is eventually constant then its constant symbol (which is a state of ${T}$) is good.

#### Definition 2

Define $L_{T}\subseteq (S_C)^\omega$ to be the following language:

$$\begin{aligned} \begin{array}{l} L_{T}= \left\{ \xi \in (S_C)^\omega | \xi \textit{ is the sequence of labels of some} \right. \\ \qquad \qquad \qquad \qquad \qquad \qquad \qquad \qquad \qquad \qquad \left. \textsc {good}\ \textit{run of } {T}\right\} . \end{array} \end{aligned}$$

Note that $L_{T}$ is $\omega$-regular and can be recognized by an NBW *W* of size $O(|S_{T}|)$ that simulates ${T}$ and stores in its second component whether or not the last simulated transition changed the state of ${T}$; a run is accepting if either there are infinitely many changes or some good state is seen infinitely often. Formally, define the NBW $W = (\varSigma _W, Q_W, I_W, \varDelta _W^I \cup \varDelta _W^0 \cup \varDelta _W^1, F_W)$:

- $$\varSigma _W = \varSigma _{T}$$,
- $$Q_W = S_{T}\times \{0,1\}$$,
- $$I_W = \{(\iota ,0)\}$$,
- $\varDelta _W^I$ consists of transitions $(\iota ,0) \xrightarrow {\iota _C} (s,i)$ if and only if $(\iota ,\iota _C,s) \in R_{T}$, where $i \in \{0,1\}$,
- $\varDelta _W^0$ consists of transitions $(s,i) \xrightarrow {c} (s,0)$ if and only if $(s,c,s) \in R_{T}, s = (c,Y), i \in \{0,1\}$.
- $\varDelta _W^1$ consists of transitions $(s,i) \xrightarrow {c} (t,1)$ if and only if $(s,c,t) \in R_{T}, s \ne t, t = (c, Y), i \in \{0,1\}$,
- $$F_W = \{(s,0): s ~ \textit{is} ~ \textsc {good}\} \cup \{(s,1): s \in S_{T}\}$$.

Thus, by Lemma 1, $(L_{T})^\delta$ is recognized by an NBW *K*(*C*, *U*) whose size is linear in $|S_{T}| \times |\varSigma _{T}|$, i.e., $O(|C|^2 \times 2^{|U|})$.

To complete the proof of the theorem we show that $\textsc {controller-exec}_{}(C,U) = (L_{T})^\delta$.

#### Notation

For a tuple $\bar{u} = (u_1,\ldots , u_j)$ write $set(\bar{u})$ for the set $\{u_1,\ldots , u_j\}$.

#### Claim A

$$\textsc {controller-exec}_{}(C,U) \subseteq (L_{T})^\delta $$.

#### Proof of Claim A

Fix $w = c_0 c_1 \ldots$ from language $\textsc {controller-exec}_{}(C,U)$. Let $\pi$ be a state-labeled run in some DG system, say with *N* user processes, that generates *w*, i.e., $w = proj_v(\pi )$ where *v* is the vertex of the controller. Partition $\pi {:=} \rho _0 \rho _1 \rho _2 \ldots$ into segments so that if $(c,\bar{u})$ is in $\rho _i$ then $c = c_i$ (pick any partition if there is more than one, which happens if there are successive configurations of $\pi$ with the same controller components). Define sets $Y_i$ as follows: $Y_0 {:=} \textit{Reach}(c_0, \{\iota _U\})$ and $Y_{i+1} {:=} \textit{Reach}(c_{i+1}, Y_i)$. It is enough to show that $\alpha {:=} \iota \xrightarrow {c_0} (c_0,Y_0) \xrightarrow {c_1} (c_1,Y_1) \ldots$ is a good run of ${T}$ since its sequence of actions is exactly *w*.

**Note.** If $(c,\bar{u})$ occurs in $\rho _i$ and $s \in set(\bar{u})$ then $s \in Y_i$. This can easily be proved by induction on *i* (use the fact that $y \in Reach(c,Y)$ if there exists $M \in \mathbb {N}$ and a finite path in a DG system with *M* user processes starting with a configuration $(c,\bar{u}) \in S_C \times Y^M$ with two properties: (i) the first co-ordinate of every configuration in the path is *c*, and ii) the path ends in a configuration $(c,\bar{v})$ such that $v_j = y$ for some $j \le M$).

By the Note, the transition (in *C*) from the end of $\rho _i$ to the beginning of $\rho _{i+1}$ is guarded, if at all, by a state in $Y_i$. Thus, by the definition of $R_{T}$, $\alpha$ is a run of ${T}$. We now prove that $\alpha$ is good. To this end, suppose that the state-labelled run induced by $\alpha$, i.e., $\iota (c_0,Y_0) (c_1,Y_1) \ldots$, is eventually constant, say with constant symbol (*c*, *Y*), and that there is no $\gamma \in Y \cup \{\tau \}$ such that $(c,\gamma ,c) \in R_C$ (otherwise there is nothing to do). Thus, there exists $m \in \mathbb {N}$ such that for all $i \ge m$, $\pi _i \in \{c\} \times 2^{S_U}$, and each transition from $\pi _i$ to $\pi _{i+1}$ is due to some user process taking a transition. Since there are only finitely many processes (i.e., *N*), some user process, say the *K*th, must make be responsible for infinitely many transitions. However, since the process template *U* is finite, the *K*th user process must eventually trace a cycle in *U*. By the Note, the cycle can be chosen to start and end at some element of *Y*, and every guard on the cycle is in $Y \cup \{c,\tau \}$. Thus, (*c*, *Y*) is good. This completes the proof Claim A. $\square$

#### Claim B

$$(L_{T})^\delta \subseteq \textsc {controller-exec}_{}(C,U)$$.

#### Proof of Claim B

Fix $w \in (L_{T})^\delta$, say $w = c_0 c_1 \ldots$. In order to prove the claim, we will build a run $\pi$ in a system with one control process and $2 N^{N+1}$ user processes, where $N {:=} |U|$, so that *w* is the destuttering of the projection of $\pi$ onto *C*.

Let $\alpha$ be any run in ${T}$ such that *w* is the destuttering of the actions in $\alpha$, say $\alpha = \iota \xrightarrow {d_0} (d_0,Y_0) \xrightarrow {d_1} (d_1,Y_1)\ldots$. Note that there exists $m \in \mathbb {N}_0$ such that $Y_m = Y_j$ for all $j \ge m$ (this is because the sequence $\{Y_n\}_{n \ge 0}$ is monotone and contained in the finite set $S_U$).

Intuitively, in order to build $\pi$ so that controller traces a path whose destuttering is *w*, we will ensure that the $\pi$ reaches a configuration in which at least two processes are in every state of $Y_m$: we need at least one process in every state of $Y_m$ to enable all the guards that may be used in the future; but we may also need an additional process in a state *y* in case we need to make one process perform a cycle starting and ending with *y* (this case occurs if *w* is eventually constant, say with constant symbol (*c*, *Y*), and there is no self-loop in $R_C$ of the form $(c,\gamma ,c)$ for $\gamma \in Y \cup \{\tau \}$). In order to reach such a configuration we will repeatedly apply Lemma 4, starting with $L = 2$, for (at most) *N* steps. In particular, we may require $2N^{N}$ user processes to start with.

We first need some definitions. Define $X \subset \mathbb {N}_0$ to be the set of indices $i \in \mathbb {N}_0$ such that either $i = 0$ or $|Y_i| \ne | Y_{i-1} |$. Since $Y_0$ is non-empty, $|X| \le N$. List the elements of *X* as $x_0 = 0< x_1< x_2< \cdots < x_{|X|} \le m$. For $i \in \mathbb {N}$ write $\beta (i)$ for the largest integer $j \le |X|$ such that $x_j \le i$. For $i \in [0,N+1]$, define $\varGamma _i {:=} 2N^{N-i}$. Note that $N \varGamma _{i+1} =\varGamma _i$, and $\varGamma _{N} = 2$, and $\varGamma _0 = 2N^N$.

We now describe how to build $\pi$ by iterating over the transitions of $\alpha$. After step $i \le m$ (i.e., after considering the transition with target $(d_i,Y_i)$) we will have built a path that ends in a state whose first co-ordinate is $d_i$, say $(d_i,\bar{v})$, with the following *invariant*: every $y \in Y_i$ appears in $\bar{v}$ at least $\varGamma _{\beta (i)}$ times.

We begin with the transition $\iota \rightarrow (d_0,Y_0)$. Apply Lemma 4 with $L {:=} \varGamma _0$ and initial configuration $(\iota _C, \bar{u})$ where $set(\bar{u}) = \{\iota _U\}$ and $|\bar{u}| = LN$, to get a path starting with $(\iota _C, \bar{u})$ and ending with a configuration $(\iota _C,\bar{v})$ where $set(\bar{v}) = Reach(\iota _C,\{\iota _U\})$ and each element in this set appears in $\bar{v}$ at least $\varGamma _0$ times (thus maintaining the invariant).

Suppose we have processed the transition with target $(d_{i-1},Y_{i-1})$ and, thus, the path built so far, say $\pi ^{\prime }$, ends with $(d_{i-1},\bar{v})$ and satisfies the invariant, i.e., every $y \in Y_{i-1}$ appears in $\bar{v}$ at least $\varGamma _{\beta (i-1)}$ times. Consider the transition $(d_{i-1},Y_{i-1}) \xrightarrow {d_i} (d_i,Y_i)$ in $\pi$. There are two cases.

1. Case $i \in X$. Since, in this case, $Y_i \ne Y_{i-1}$, the transition must be in $R^{\textsc {Prog}}$, i.e., there is some $\gamma \in \{\tau \} \cup Y_{i-1}$ such that $(d_{i-1},\gamma ,d_{i}) \in R_C$. By the invariant, $Y_{i-1} \subseteq set(\bar{v})$. Conclude that $(d_{i-1},\bar{v}) \rightarrow (d_{i},\bar{v})$ is a transition of the DG system. Thus, first extend $\pi ^{\prime }$ by the state $(d_{i},\bar{v})$. Second, since $i \in X$ we have that $i = x_k$ for some *k*, and thus $\beta (i) = k$; also, $x_{k-1} \le i-1 < x_k$, and thus $\beta (i-1) = k-1$. Conclude that $\varGamma _{\beta (i-1)} = N\varGamma _{\beta (i)}$. By the invariant we can apply Lemma 4 with $L {:=} \varGamma _{\beta (i)}$ and configuration $(d_{i},\bar{v})$. This results in a path *p* that starts with $(d_{i},\bar{v})$ and ends with configuration of the form $(d_{i+1},\bar{w})$ where every state in $Y_{i+1} = Reach(d_{i+1},Y_i)$ appears in $\bar{w}$ at least $\varGamma _{\beta (i)}$ times. Now extend $\pi ^{\prime }$ by *p*. Note that the invariant is maintained.
2. Case $i \not \in X$. Thus, in this case, $Y {:=} Y_i = Y_{i-1}$. There are two subcases.
  Subcase $d_{i-1} \ne d_i$. In this subcase, the transition must be in $R^{\textsc {Prog}}$. As in the first half of the case $i \in X$, the DG systems has a transition $(d_{i-1},\bar{v}) \rightarrow (d_{i},\bar{v})$. Extend $\pi ^{\prime }$ by the state $(d_{i},\bar{v})$ and note that the invariant is maintained since no user process moved.
  Subcase $d {:=} d_{i-1} = d_i$. This subcase can be ignored, i.e., do not extend $\pi ^{\prime }$. Again, the invariant is maintained since no user process moved.

At this point we have constructed a finite path $\pi ^{\prime }$ that mimics the first *m* steps of $\alpha$. Recall that $Y_m = Y_j$ for all $j \ge m$. To finish, we identify two cases. If $\alpha$ is not eventually constant, then repeatedly apply the reasoning in case 2 above. On the other hand, if $\alpha$ is eventually constant, say with constant symbol $(d_n,Y_n)$ (for some $n \ge m$), then extend $\pi ^{\prime }$ to mimic the transitions between *m* and *n* (as in Case 2 above), and then proceed as follows. Since $(d_n,Y_n)$ is $\textsc {good}$ there are two subcases. If there is a self-loop $(d_n,\gamma ,d_n)$ in $R_C$ for some $\gamma \in \{\tau \} \cup Y_n$, then extend $\pi ^{\prime }$ by the infinite path $(d_n,Y_n)^\omega$. Otherwise, by the definition of $\textsc {good}$, there is a cycle in *U* starting and ending in some $y \in Y_n$ such that each transition is guarded by an element of $Y_n \cup \{d_n,\tau \}$; thus we can extend $\pi ^{\prime }$ by transitions in which a process at $y_n$ repeatedly makes this cycle (note that this is possible since up till now we guaranteed that there are at least 2 processes in every state of $Y_n$). This completes the proof Claim B. $\square$

To summarise, we have shown that ${\textsc {controller-exec}} (C,U) = (L_{T})^\delta$ and that there is an NBW of size $O(|S_C|^2 \times 2^{|S_U|})$ recognizing $(L_{T})^\delta$. $\square$

Now we can generalize the previous result, showing that model checking disjunctively-guarded systems in homogeneous parameterized topologies can be done with an NBW that is exponential in the size of the input models.

#### Theorem 18

Let ${\mathcal G}$ be the *r*-ary homogeneous parameterized topology. Let $\textit{Ctr} = B_{sng}$ and $\textit{Usr} = B_{ind} \cup B_{clq}$. For every disjunctively-guarded system template $\overline{P}= (P_1, \ldots , P_n)$, for each $i \in \textit{Ctr}$ (resp. $i \in \textit{Usr}$) there is an NBW $K(\overline{P})$ of size $O(c^2 \times 2^{u})$ recognizing $\textsc {i-exec}_{{\mathcal G}}{(\overline{P})}$, where $c = \varPi _{i \in \textit{Ctr}} |P_i|$ and $u = 1 + \varSigma _{i \in \textit{Usr}} |P_i|$.

#### Proof

By Lemma 3, we can reduce $\overline{P}$ to two templates: *C* is the product of the controllers, and *U* is the union of the user process. Next, we can apply Theorem 17 to produce an NBW *K*(*C*, *U*) of size $O(|C|^2 \times 2^{|U|})$ recognizing the executions of *C* (and similarly, an NBW for the executions of *U*). In case $i \in \textit{Ctr}$, form an NBW from *K*(*C*, *U*) by projecting onto the *i*-th component of the state, and thus isolate only the executions of the *i*-th controller of the original system. In case $i \in \textit{Usr}$, form an NBW from *K*(*C*, *U*) by intersecting with an automaton whose language is all runs through process $P_i$. In both cases, the produced NBW is linear in the size of *K*(*C*, *U*), concluding the proof. $\square$

### Complexity of PMCP

The following theorem states the complexity of PMCP for homogeneous parameterized topologies. It derives the complexity upper bound from the automata theoretic approach and constructing the NBW in Theorem 17 “on the fly”.

#### Theorem 19

Let ${\mathcal G}$ be an *r*-ary homogeneous parameterized topology (controlled or controllerless). Let ${\mathcal F}$ be the set of 1-index $\textsf {LTL}\backslash \textsf {X}$ formulas, and let ${\mathcal {P}}$ be the set of disjunctively-guarded *r*-ary system templates. The complexity of ${\textsf {PMCP}}_{\mathcal G}({\mathcal {P}},{\mathcal F})$ is Pspace-complete.

#### Proof

We begin with the lower bound. Pspace hardness follows from the fact that $\textsf {LTL}\backslash \textsf {X}$ model checking of a Kripke structures is Pspace-hard [50] and the observation that this problem is a special case of PMCP. Indeed, given a Kripke structure, one can think of it as a process template all of whose transitions are silent (i.e., there is no communication). Since there is no communication, the topology plays no role, and the execution traces of all the processes of the same type (be it user or controller) running at any node are exactly the same. Hence, in all cases, the PMCP degenerates to model checking of Kripke structures.

We now address the upper bound. First, observe that the controllerless case can be immediately reduced to the controlled case simply by having a controller that runs the same process template as the other processes (i.e., by having $C=U$). Second, by Lemma 3, it is enough to consider the case of a 2-ary controlled clique parameterized topology.

Given process templates *C*, *U*, by Theorem 17 there is an NBW *K*(*C*, *U*), whose language is

$\textsc {controller-exec}_{}(C,U)$ or $\textsc {user-exec}_{}(C,U)$, as we wish. Given a 1-index $\textsf {LTL}\backslash \textsf {X}$ specification formula $\psi$, we can decide, by Corollary 1, the PMCP for $\psi$ by checking for the non-emptiness of the product of $A_{\lnot \phi ^{\prime }}$ and $K^{\prime }(C,U)$: $\phi ^{\prime }$ is the maximal $\textsf {LTL}\backslash \textsf {X}$ subformula of $\psi$ such that every atom of the form (*a*, *x*) has been replaced by the atom *a*; $K^{\prime }(C,U)$ is the same NBW as *K*(*C*, *U*) except that every transition label $c \in S_C$ is replaced by $\varPhi _C(c) \in 2^{\textsf {AP}}$. Furthermore, by [53], this non-emptiness problem can be solved in Pspace as long as storing a state of *K*(*C*, *U*), as well as checking membership in the transition relation of *K*(*C*, *U*), can be done in polynomial space. Since it is not hard to see that this is indeed the case, the required upper bound follows.$\square$

The next theorem states the program complexity of PMCP for clique parameterized topologies. For controllerless systems we inherit the Ptime program complexity from the NBW used to recognize the executions of a process in homogeneous topologies with pairwise rendezvous (see Theorem 7). With a controller, the co-NP  upper bound results from a fine analysis of the construction in the proof of Theorem 17, and the co-NPhardness by coding of propositional unsatisfiability: the user processes store an assignment, and the controller verifies it is not satisfying.

#### Theorem 20

Fix ${\mathcal F}$ to be the set of 1-index $\textsf {LTL}\backslash \textsf {X}$ formulas. If ${\mathcal {P}}$ is the set of disjunctively-guarded 1-ary system templates, and ${\mathcal G}$ is the 1-ary clique parameterized topology, then the program complexity of ${\textsf {PMCP}}_{\mathcal G}({\mathcal {P}},{\mathcal F})$ is in Ptime. If ${\mathcal {P}}$ is the set of disjunctively-guarded 2-ary system templates, and ${\mathcal G}$ is the 2-ary controlled clique parameterized topology, then the program complexity of ${\textsf {PMCP}}_{\mathcal G}({\mathcal {P}},{\mathcal F})$ is co-NP-complete.

#### Proof

Without a controller, membership in P follows from [36, Section 4]. With a controller, membership in co-NPcan be derived using a more careful analysis of the complexity of the PMCP performed in the proof of Theorem 19, as follows.

In that proof it has been shown that deciding whether $\forall G\in {\mathcal G}, \overline{P}^G\models \psi$ can be done by checking for the non-emptiness of the product NBW $A = A_{\lnot \phi ^{\prime }} \times K^{\prime }(C,U)$, for a suitable formula $\phi ^{\prime }$ (from which the NBW $A_{\lnot \phi ^{\prime }}$ is derived) and NBW $K^{\prime }(C,U)$. We recall from that proof that $\phi ^{\prime }$ is the maximal $\textsf {LTL}\backslash \textsf {X}$ subformula of the 1-indexed $\textsf {LTL}\backslash \textsf {X}$ original formula $\psi$, whose atoms with form (*a*, *x*) are replaced by atom *a*. The NBW $K^{\prime }(C,U)$, instead, is derived from *K*(*C*, *U*) (see Theorem 17) where transition label $c \in S_C$ are replaced by $\varPhi _C(c) \in 2^\textsf {AP}$.

Checking for the non-emptiness of an NBW amounts to finding a lasso in it. I.e., to finding a state *s*, and two simple paths: one from the initial state to *s*, and the other from *s* back to itself. Observe that a lasso in the product automaton *A* induces lassos *x*, *y* in $A_{\lnot \phi ^{\prime }}$ and $K^{\prime }(C,U)$, respectively. Hence, checking that *A* is not empty can be done by guessing the lassos *x*, *y* and checking that their product is indeed a lasso in *A*.^10^ Looking at the proof of Theorem 17, one can see that (except for one state) all the states of the LTS ${T}$ are of the form (*c*, *Y*), where *c* is some state of *C*, and *Y* is a subset of the states of *U*. Furthermore, there is a transition between two such states $(c,Y), (c^{\prime },Y^{\prime })$ only if $Y \subseteq Y^{\prime }$. It follows that the longest simple path of ${T}$ is of length at most $|C| \times |U|$. Thus, since $K^{\prime }(C,U)$ is formed by taking the product of ${T}$ with an automaton of size *O*(|*C*|),^11^ the length of the longest simple path, and thus also of the longest lasso, of $K^{\prime }(C,U)$ is of length $O(|C| ^2 \times |U|)$. Overall, since we also have that querying the transition relation of $K^{\prime }(C,U)$ is cheap, we conclude that one can guess any lasso in $K^{\prime }(C,U)$ in time polynomial in $|C| \times |U|$.

Recall that when analyzing program complexity, we consider the formula $\psi$ to be constant. Thus, we get that one can, in nondeterministic polynomial time, guess any pair of lassos *x*, *y* in $A_{\lnot \phi ^{\prime }}$ and $K^{\prime }(C,U)$. It is not hard to see that given *x*, *y*, checking that their product is indeed a lasso in *A* can be done in time polynomial in the size of these lassos. It follows that one can guess and verify in nondeterministic polynomial time that the automaton *A* is not empty, and thus, that it is not the case that $\forall G\in {\mathcal G}, \overline{P}^G\models \psi$, which gives the desired membership of PMCP in co-NP.

For the lower bound, we reduce the unsatisfiability problem of a 3-SAT formula to the PMCP. Given a 3-SAT formula $\bigwedge _{i=1}^m (c_{i1} \vee c_{i2} \vee c_{i3})$ over the Boolean variables $x_1, \ldots , x_n$, we build the two process templates *C*, *U* given in Figs. 7 and 8, and consider computations in which the controller *C* reaches the state *done*. Note that, for every $1 \le i \le n$, the way to the state *done* goes either through $x_i^\top$ or through $x_i^\bot$, and that one can transition out of these states only if at least one user process *U* enables the corresponding guard ($x_i$ or $\overline{x_i}$). Also note that ($\dagger$): the guards on the transitions of *U* ensure that if the controller entered $x_i^\top$ (resp. $x_i^\bot$), then no user process can be (anywhere along the computation) in state $\overline{x_i}$ (resp. $x_i$).

**Fig. 9:**
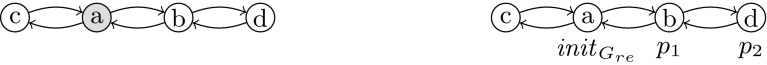
Running example: the topology $$G_{re}$$ and the graph LTS $${{G_{re}}}|{\bar{g}}$$ for $$\bar{g}=(b,d)$$. The 2-topology $$G_{re}$$ with $$T_1 = \{a\}$$ and $$T_2 = \{b,c,d\}$$ is depicted to the *left*. The graph LTS $${{G_{re}}}|{\bar{g}}$$ is depicted to the *right*. $${{G_{re}}}|{\bar{g}}$$ has atomic propositions $$\textsf {AP}= \{p_1,p_2\}$$, initial state $$ init _{G_{re}} = a$$, and state-labeling function $$\varLambda (a)=\varLambda (c)=\emptyset $$, $$\varLambda (b)=\{p_1\}$$ and $$\varLambda (d)=\{p_2\}$$. All of the transitions of $${{G_{re}}}|{\bar{g}}$$ are labelled $$\mathsf {tok}$$

**Fig. 10:**
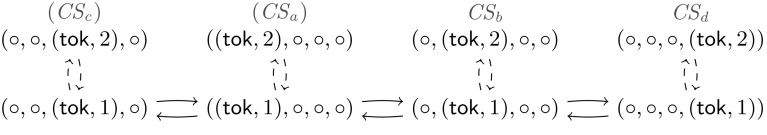
Running example: the token-passing system $$\overline{P}^{G_{re}}$$ and the projection LTS $${{\overline{P}^{G_{re}}}}|{\bar{g}}$$. This figure depicts the token-passing system with topology $$G_{re}$$ and process templates $$\overline{P} = (P_\mathsf {tok},P_\mathsf {ntok})$$. We represent the configurations of $$(P_\mathsf {tok},P_\mathsf {ntok})^{G_{re}}$$ as 4-vectors $$(s_a,s_b,s_c,s_d)$$ containing the states of the processes at *a*, *b*, *c*, and *d* respectively. As shorthand we write $$\circ $$ for the most common state $$(\mathsf {ntok},1)$$. We omit unreachable configurations, such as those in which no process has the token or two processes have the token. The *dashed* transitions are internal transitions. The *solid* transitions are synchronous transitions (i.e., transitions where the token passes from one process to another). The configuration $$((\mathsf {tok},1),\circ ,\circ ,\circ )$$ is the initial configuration. The labeling function $$\varLambda $$ assigns $$\emptyset $$ to any configuration containing $$(\mathsf {tok},1)$$. Otherwise it assigns a singleton set $$\{ CS_X \}$$ for the corresponding $$X\in \{a,b,c,d\}$$. The projection LTS $${{\overline{P}^{G_{re}}}}|{\bar{g}}$$ for $$\bar{g}=(b,d)$$ is obtained from this figure by removing the $$\varLambda $$ labellings $$ CS _a$$ and $$ CS _c$$

It follows that if the controller reaches state $c_1$ then there were at least *n* user processes, and that the user processes store an assignment to each variable $x_i$, $1 \le i \le n$, as follows: $x_i$ is true if there is some user process in state $x_i$, and it is false if there is some user process in state $\overline{x_i}$ (note that, by $\dagger$, these two options are mutually exclusive). Observe that the controller can reach the state *done* if and only if this assignment satisfies the 3-SAT formula. Indeed, for every $1 \le j \le m$, the guard on the outgoing transition from state $c_j$ ensures that this transition can be taken if and only if the stored assignment satisfies clause $c_j$. It follows that the 3-SAT formula is unsatisfiable if and only if the PMCP for the 1-ary clique topology with process templates (*C*, *U*), and the fixed formula $\psi = \textsf {G}\lnot done$, has a positive answer.$\square$

Combining Theorem 20 and Lemma 3 we get the following corollary, extending the complexity analysis to homogeneous parameterized topologies:

#### Corollary 2

Let ${\mathcal G}$ be an *r*-ary homogeneous parameterized topology, let ${\mathcal F}$ be the set of 1-index $\textsf {LTL}\backslash \textsf {X}$ formulas, and let ${\mathcal {P}}$ be the set of disjunctively-guarded *r*-ary system templates. If ${\mathcal G}$ is controllerless then the program complexity of ${\textsf {PMCP}}_{\mathcal G}({\mathcal {P}},{\mathcal F})$ is in Ptime, and otherwise (i.e., if ${\mathcal G}$ is controlled) it is in co-NP.

## Token passing systems

In this section we show that ${\textsf {PMCP}}_{{\mathcal G}}({\mathcal {P}},{\textsf {i-CTL}}^*\backslash \textsf {X})$ is decidable, where ${\mathcal {P}}$ is the set of all process templates, and ${\mathcal G}$ is either (1) $\textsf {MSO}$-definable and of bounded clique-width or (2) iteratively constructible. We encode a characterization of the $\textsf {CTL}^*_{d}\backslash \textsf {X}$-indistinguishability equivalence relation from [6] in $\textsf {MSO}$ and utilize the *composition* property of $\textsf {CTL}^*_{d}\backslash \textsf {X}$ proved there. We prove the existence of decidable cutoffs for the PMCP problem in this setting and show a lower bound on the cutoffs of iteratively-constructible parameterized topologies for indexed $\textsf {LTL}\backslash \textsf {X}$.

The section is organized as follows. Section 5.1 introduces the necessary background with regards to $\textsf {CTL}^*_{d}\backslash \textsf {X}$ on token-passing systems. Section 5.2 gives preliminaries regarding topologies of bounded clique-width and the Monadic Second Order Logic of topologies. Section 5.3 proves the decidability of the PMCP problem and the existence of computable cutoffs. Section 5.4 discusses sizes of the cutoffs.

In this section we will have a running example which we will revisited several times in Running Examples 1, 2, 3, 4, and 5, and Figs. 9 and 10.

### Running Example 1

Let $G_{re} = (V_{re},E_{re},T_1,T_2)$ be the 2-topology depicted in Fig. 9.

We define $P_1 = P_\mathsf {tok}$ to be the process template with:

- state set $$S=\{(\mathsf {ntok},1),(\mathsf {tok},1),(\mathsf {tok},2)\}$$,
- atomic propositions set $\textsf {AP}_{\textsf {pr}}= \{\mathrm {CS}\}$,
- state labeling $\varPhi ((\mathsf {ntok},1)) = \varPhi ((\mathsf {tok},1)) = \emptyset$ and $\varPhi ((\mathsf {tok},2)) = \{\mathrm {CS}\}$,
- synchronization alphabet $$\varSigma _{\textsf {sync}}= \{\mathsf {tok}\}$$,
- transition relation
  $$\begin{aligned} \begin{array}{lll} R &{}=&{} \{((\mathsf {ntok},1),\mathsf {tok}?,(\mathsf {tok},1)), ((\mathsf {tok},1),\mathsf {tok}!,(\mathsf {ntok},1)),\\ &{} &{} ((\mathsf {tok},1),\tau ,(\mathsf {tok},2)),((\mathsf {tok},2),\tau ,(\mathsf {tok},1))\}, \end{array} \end{aligned}$$
  and
- initial state $$\iota _\mathsf {tok}= (\mathsf {tok},1)$$.

The process template $P_2 = P_\mathsf {ntok}$ is obtained from $P_\mathsf {tok}$ by setting the initial state to $\iota _\mathsf {ntok}= (\mathsf {ntok},1)$. Let $\overline{P} = (P_\mathsf {tok},P_\mathsf {ntok})$. The token-passing system $\overline{P}^{G_{re}}$ is depicted in Fig. 10.

### Preliminaries I: $\textsf {CTL}^*_{d}\backslash \textsf {X}$ and token-passing systems

#### Two abstractions of a token-passing system

We now define for a given TPS $\overline{P}^{G}$ two abstractions used in the above-mentioned characterization [6]. The first abstraction simulates $\overline{P}^{G}$, keeping track only of the local states of processes indexed by $\bar{g}$. We call it the *projection* of $\overline{P}^{G}$ onto $\bar{g}$. The second abstraction only simulates the movement of the token in *G*, restricted to $\bar{g}$. We call it the *graph LTS* of *G* and $\bar{g}$.

*Notation*. For topologies *G* and $G^{\prime }$, let $\bar{g}$ denote a tuple $(g_{1},\ldots ,g_{k})$ of vertices of *G*, and $\bar{g}^{\prime }$ a *k*-tuple of distinct vertices of $G^{\prime }$. Write $v\in \bar{g}$ if $v=g_{i}$ for some *i*.

*The projection* $${{\overline{P}^{G}}}|{\bar{g}}$$

Informally, the *projection* of $\overline{P}^{G}$ onto a tuple of process indices $\bar{g}$ is the LTS $\overline{P}^{G}$ and a new labeling that removes all indexed atoms $p_{j}$ for $j\notin \bar{g}$.

More precisely, fix a system template $\overline{P}$, a topology *G*, and a *k*-tuple $\bar{g}$ over $V_{G}$. Say $\overline{P}^{G}=(Q,\varDelta ,Q_{0},\varLambda )$. Define the *projection of* $\overline{P}^{G}$ *onto* $\bar{g}$, written ${{\overline{P}^{G}}}|{\bar{g}}$ as the LTS $(Q,\varDelta ,Q_{0},L)$ where for all $q\in Q$ the labeling *L*(*q*) is defined as $L(q){:=}\varLambda (q)\cap \{p_{g_{i}}\mid p\in \textsf {AP}_{\textsf {pr}},i\in [k]\}$.

*The graph LTS* ${{G}}|{\bar{g}}$

Informally, ${{G}}|{\bar{g}}$ is an LTS where the states are the vertices of the *r*-topology *G*, and the transitions are the edges of *G*. The graph LTS simulates the passing of the token between the vertices of *G*, beginning with the token at the unique vertex $init _G \in V$ belonging to $T_1$. The vertices $g_1,\ldots ,g_k$ are assigned the atomic propositions $p_1,\ldots ,p_k$, respectively.

The precise definition of the graph LTS is as follows. Let $G = (V,E,T_1,\ldots ,T_r)$ be an *r*-topology such that $|T_1|=1$, and let $\bar{g}=(g_1,\ldots ,g_k)$ be a *k*-tuple of *G* vertices. The graph LTS ${{G}}|{\bar{g}}$ is the LTS $(V,\varDelta ,V_0,\varLambda ,\textsf {AP},\varSigma )$ in which:

- the set of states is *V*,
- the alphabet $\varSigma$ of transition labels is $\{\mathsf {tok}\}$,
- the set of atomic propositions $\textsf {AP}$ is $\{p_1,\ldots ,p_k\}$,
- the transition relation $\varDelta$ is
  $$\begin{aligned} \{(u,\mathsf {tok},v) \mid (u,v)\in E\} \end{aligned}$$
- the set of initial states $V_0$ is $T_1=\{ init _G\}$, and
- the state-labeling function $\varLambda$ of $v\in V$ is $\varLambda (v) = \{p_i \mid v = g_i\}$.

#### $\textsf {CTL}^*_{d}\backslash \textsf {X}$-equivalence and $\textsf {CTL}^*_{d}\backslash \textsf {X}$-character

Recall that $$\textsf {CTL}^*_d$$ is the fragment of $$\textsf {CTL}^*$$ with at most *d* path quantifiers. For two LTSs $$ LTS _1$$ and $$ LTS _2$$, we write that $$ LTS _1$$ and $$ LTS _2$$ are $$\textsf {CTL}^*_{d}\backslash \textsf {X}$$-*equivalent* if they agree on all $$\textsf {CTL}^*_{d}\backslash \textsf {X}$$ formulas: for every $$\textsf {CTL}^*_{d}\backslash \textsf {X}$$ formula $$\phi $$ it holds that $$ LTS _1 \models \phi $$ iff $$ LTS _2 \models \phi $$. We denote that $$ LTS _1$$ and $$ LTS _2$$ are $$\textsf {CTL}^*_{d}\backslash \textsf {X}$$-equivalent by $$ LTS _1 \equiv _{\textsf {CTL}^*_{d}\backslash \textsf {X}} LTS _2$$. Note that the definition of $$\textsf {CTL}^*_{d}\backslash \textsf {X}$$ *-equivalence* applies in particular to our two abstractions, the projection LTS of $$\overline{P}^{G}$$ onto $$\bar{g}$$ and the graph LTS of *G* and $$\bar{g}$$.

**The composition property**

The composition theorem says that the $\textsf {CTL}^*_{d}\backslash \textsf {X}$-equivalence of projections ${{\overline{P}^{G}}}|{\bar{g}}$ and ${{\overline{P}^{H}}}|{\bar{h}}$ can be reduced to the $\textsf {CTL}^*_{d}\backslash \textsf {X}$-equivalence of their graph LTSs ${{G}}|{\bar{g}}$ and ${{H}}|{\bar{h}}$. The composition property says that if two graph LTSs are indistinguishable, then so are their corresponding projections.

##### Theorem 21

(The composition Theorem [6]) For every $k,d\in \mathbb {N}$, system template $\overline{P}\in {\mathcal {P}}$, topologies *G*, *H*, and *k*-tuples ${\bar{g}}$ and ${\bar{h}}$ of vertices of *G* and *H* respectively:

$$\begin{aligned} {{G}}|{\bar{g}}\equiv _{\textsf {CTL}^*_{d}\backslash \textsf {X}}{{H}}|{\bar{h}} \,\,{ implies}\,\, {{\overline{P}^{G}}}|{\bar{g}}\equiv _{\textsf {CTL}^*_{d}\backslash \textsf {X}}{{\overline{P}^{H}}}|{\bar{h}} \end{aligned}$$

The $\equiv _{\textsf {CTL}^*_{d}\backslash \textsf {X}}$-equivalence class of a graph LTS is uniquely determined by a $(k+1)$-vector which is called the $\textsf {CTL}^*_{d}\backslash \textsf {X}$ *-character*. This vector consists of pairs of labellings $\varLambda$ and *markings* $\varXi ^k_d$.

Fix $k,d\in \mathbb {N}$, topology $G=(V,E,\overline{T})$, and *k*-tuple ${\bar{g}}$ over *V*. Let ${{G}}|{\bar{g}} = (V,\varDelta ,V_{0},\varLambda )$ be the graph LTS of *G* and $\bar{g}$. We will recursively define below a marking function $\varXi _d^k$ that associates with each vertex $v \in V$ a $(k+1)$-dimensional vector $\varXi _d^k(v)$ whose *i*th coordinate $\varXi ^k_d(v)[i]$ is a set of strings over the alphabet

$$\begin{aligned} \{\varXi ^k_{d-1}(u): u\in V\}. \end{aligned}$$

The $\textsf {CTL}^*_{d}\backslash \textsf {X}$ *-character* of ${{G}}|{\bar{g}}$ is the $(k+1)$-tuple:

$$\begin{aligned}&\left( \left\langle \varLambda ( init _G), \varXi ^k_d( init _G)\right\rangle , \left\langle \varLambda (g_1), \varXi ^k_d(g_1) \right\rangle , \ldots ,\right. \\&\quad \left. \left\langle \varLambda (g_k), \varXi ^k_d(g_k) \right\rangle \right) . \end{aligned}$$

The crucial properties of the $\textsf {CTL}^*_{d}\backslash \textsf {X}$-character are:

- The $\textsf {CTL}^*_{d}\backslash \textsf {X}$-character determines whether ${{G}}|{\bar{g}}\models \varphi$ for every formula $\varphi \in \textsf {CTL}^*_{d}\backslash \textsf {X}$.
- The number of $\textsf {CTL}^*_{d}\backslash \textsf {X}$-characters for any fixed *d* and *k* is finite and computable. We discuss the set $\varUpsilon ^k_d$ containing all $\textsf {CTL}^*_{d}\backslash \textsf {X}$-characters for *k* and *d* below.

*The marking* $$\varXi ^k_d$$

For every vertex $v\in V$, let $v^\leadsto$ be the set of maximal paths in *G* starting in *v* that have no intermediate vertices in $\bar{g}$, i.e.:

1. an infinite path $\pi = v_1,v_2,\ldots$ is in $v^\leadsto$ iff $v_1 = v$ and $v_i\notin \bar{g}$ for all $i> 1$;
2. a finite path $\pi = v_1,v_2,\ldots ,v_s$ is in $v^\leadsto$ iff $v_1 = v$, $s>1$, $v_s \in \bar{g}$ and $v_i\notin \bar{g}$ for all $1<i<s$.

We write $v^{\leadsto 0}$ for the set of infinite paths in $v^\leadsto$. For every $j\in [k]$, we write $v^{\leadsto j}$ for the set of $v^\leadsto$ paths which end in $g_j$.

##### Running Example 2

We write $L(\mathrm {Reg})$ for the language of the regular expression $\mathrm {Reg}$. For ${{G_{re}}}|{\bar{g}}$ with $\bar{g}=(g_1,g_2)$, $g_1 = b$, and $g_2 = d$, we have:

$$\begin{aligned} \begin{array}{lllllllllll} a^{\leadsto 0} &{}=&{} \{(ac)^\omega \} &{} &{}\quad a^{\leadsto 1} &{}=&{} L((ac)^\star ab) &{} &{} \quad a^{\leadsto 2} &{}=&{} \emptyset \\ b^{\leadsto 0} &{}=&{} \{b(ac)^\omega \} &{} &{}\quad b^{\leadsto 1} &{}=&{} L(b(ac)^\star ab) &{} &{} \quad b^{\leadsto 2} &{}=&{} \{bd\} \\ c^{\leadsto 0} &{}=&{} \{c(ac)^\omega \} &{} &{}\quad c^{\leadsto 1} &{}=&{} L(c(ac)^\star ab) &{} &{} \quad c^{\leadsto 2} &{}=&{} \emptyset \\ d^{\leadsto 0} &{}=&{} \emptyset &{} &{} \quad d^{\leadsto 1} &{}=&{} \{db\} &{} &{} \quad d^{\leadsto 2} &{}=&{} \emptyset \end{array} \end{aligned}$$

For a (finite or infinite) path $\pi = v_1,v_2,\ldots$ we denote by $\varXi ^k_d(\pi )$ the concatenation of the *d* markings of the vertices of $\pi$, i.e., $\varXi ^k_d(\pi ) = \varXi ^k_d(v_1)\varXi ^k_d(v_2)\ldots$. We define the *marking* $\varXi ^k_d$ of a vertex inductively (on *d*) as follows:

- $$\varXi ^k_0(v) = \varLambda (v)$$, for every $$v\in V$$;
- For $d>0$, $\varXi ^k_d(v)$ is the $(k+1)$-vector
  $$\begin{aligned} (\varXi ^k_d(v)[0],\ldots ,\varXi ^k_d(v)[k]) \end{aligned}$$
  where
  $$\begin{aligned} \begin{array}{llcl} \varXi ^k_d(v)[0] &{}=&{} \displaystyle {\bigcup _{\pi \in v^{\leadsto 0}}} &{} \{\mathsf {destutter}(\varXi ^k_{d-1}(\pi ))\}\\ \varXi ^k_d(v)[i] &{}=&{} \displaystyle {\bigcup _{\begin{array}{c} \pi =(\pi _0,v_s)\in v^{\leadsto i} \\ \pi _0=(v_1,v_2,\ldots ,v_{s-1}) \end{array}}}&\{\mathsf {destutter}(\varXi ^k_{d-1}(\pi _0))\} \end{array} \end{aligned}$$
  for every $i\in [k]$. The union in $\varXi ^k_d(v)[i]$ ranges over paths $\pi =(v_1,\ldots ,v_s)$.

That is, for $d=0$ the marking $\varXi ^k_0(v)$ is the label $\varLambda (v)$. For $d>0$ the marking $\varXi ^k_d$ is a vector of sets of strings, where the *i*th coordinate of the vector contains the set of strings obtained by de-stuttering the $\varXi ^k_{d-1}$ markings of the vertices of paths in $v^\leadsto$, excluding the last vertex of those paths which are finite. For every $0 \le i\le k$ and $d>0$, the marking $\varXi ^k_d(v)[i]$ is a set of strings over the alphabet $\{\varXi ^k_{d-1}(u): u\in V\}$, and all strings in $\varXi ^k_d(v)[i]$ start with the letter $\varXi ^k_{d-1}(v)$. Note that for an infinite path $\pi$, $\mathsf {destutter}(\varXi ^k_{d-1}(\pi ))$ is a finite string by Lemma 5 below.

We have:

##### Theorem 22

([6]) For every $k,d\in \mathbb {N}$, topologies $G,G^{\prime }$, and *k*-tuples ${\bar{g}},{\bar{g}^{\prime }}$: If ${{G}}|{\bar{g}}$ and ${{G^{\prime }}}|{\bar{g}^{\prime }}$ have the same $\textsf {CTL}^*_{d}\backslash \textsf {X}$-character, then ${{G}}|{\bar{g}}\equiv _{\textsf {CTL}^*_{d}\backslash \textsf {X}}{{G^{\prime }}}|{\bar{g}^{\prime }}$.

##### Running Example 3

For every $v\in \{a,b,c,d\}$, $\varXi ^k_0(v)$ is a member of the power set $2^{\{p_1,p_2\}}$ of $\{p_1,p_2\}$, and $\varXi ^k_1(v)$ is a set of strings over the alphabet $\varSigma _1 = 2^{\{p_1,p_2\}}$. For readability of the values of $\varXi ^k_1(v)$, we underline the letters of $\varSigma _1$ (e.g., we write $\underline{\{p_1\}}$ rather than $\{p_1\}$).

$$\begin{aligned}&\begin{array}{llllllll} \varXi ^2_0(a) &{}=&{} \emptyset &{} &{} \quad \varXi ^2_1(b)[0] &{}=&{} \{\underline{\{p_1\}}\underline{\emptyset }\} \\ \varXi ^2_0(b) &{}=&{} \{p_1\} &{} &{} \quad \varXi ^2_1(b)[1] &{}=&{} \{\underline{\{p_1\}}\underline{\emptyset }\} \\ \varXi ^2_0(c) &{}=&{} \emptyset &{} &{} \quad \varXi ^2_1(b)[2] &{}=&{} \{\underline{\{p_1\}}\} \\ \varXi ^2_0(d) &{}=&{} \{p_2\} \end{array}\\&\begin{array}{lllllllllll} \varXi ^2_1(a)[0] &{}=&{} \{\underline{\emptyset }\} &{} &{} \quad \varXi ^2_1(c)[0] &{}=&{} \{\underline{\emptyset }\} &{} &{} \quad \varXi ^2_1(d)[0] &{}=&{} \emptyset \\ \varXi ^2_1(a)[1] &{}=&{} \{\underline{\emptyset }\} &{} &{} \quad \varXi ^2_1(c)[1] &{}=&{} \{\underline{\emptyset }\} &{} &{} \quad \varXi ^2_1(d)[1] &{}=&{} \{\underline{\{p_2\}}\}\\ \varXi ^2_1(a)[2] &{}=&{} \emptyset &{} &{}\quad \varXi ^2_1(c)[2] &{}=&{} \emptyset &{} &{} \quad \varXi ^2_1(d)[2] &{}=&{} \emptyset \end{array} \end{aligned}$$

The $\textsf {CTL}^*_{1}\backslash \textsf {X}$-character of ${{G_{re}}}|{\bar{g}}$ is the 3-tuple:

$$\begin{aligned} \begin{array}{llll} \left( \left\langle \varLambda (a), \varXi ^2_1(a)\right\rangle , \left\langle \varLambda (b),\varXi ^2_1(b) \right\rangle , \left\langle \varLambda (d), \varXi ^2_1(d) \right\rangle \right) =\\ \left( \left\langle \emptyset , \left( \{\underline{\emptyset }\}, \{\underline{\emptyset }\}, \emptyset \right) \right\rangle , \left\langle \{p_1\}, \left( \{\underline{\{p_1\}}\underline{\emptyset }\},\{\underline{\{p_1\}}\underline{\emptyset }\},\{\underline{\{p_1\}}\}\right) \right\rangle , \right. \\ \left. \left\langle \{p_2\}, \left( \emptyset ,\{\underline{\{p_2\}}\},\emptyset \right) \right\rangle \right) \end{array} \end{aligned}$$

*The set* $\varUpsilon ^k_d$ *and the set of characters* ${{ Char}^d_k}$

The marking $\varXi _d^k(v)$ belong to a finite poset $\varUpsilon ^k_d$ which does not depend on *v* or *G*. We state the properties of $\varUpsilon ^k_d$ that needed in this paper in Lemma 5. We do not define $\varUpsilon ^k_d$ explicitly, since the definition is quite involved. We denote

$$\begin{aligned} \mathrm {Char^d_k} = (2^{\{p_1,\ldots ,p_k\}} \times \varUpsilon ^k_d)^{k+1} \end{aligned}$$

and we have:

##### Lemma 5

([6])

1. $$\varUpsilon ^k_0 = 2^{\{p_1,\ldots ,p_k\}}$$.
2. For every $k,d\in \mathbb {N}$, $d>0$, there is a partial order $\preceq _{d}^k$ such that $(\varUpsilon ^k_d, \preceq ^k_d)$ is a finite poset.
3. For every path $\pi$ in *G*, $\mathsf {destutter}(\varXi ^k_{d}(\pi ))$ is a strictly decreasing chain in the poset $(\varUpsilon ^k_{d-1}, \preceq ^k_{d-1})$.
4. For every $k,d \in \mathbb {N}$, $d>0$, each member of $\varUpsilon ^k_d$ is a set of $(k+1)$-vectors of sets of strictly decreasing chains in $(\varUpsilon ^k_{d-1}, \preceq ^k_{d-1})$.
5. The $\textsf {CTL}^*_{d}\backslash \textsf {X}$-character of every graph LTS ${{G}}|{\bar{g}}$ with $|\bar{g}| = k$ belongs to $\mathrm {Char^d_k}$.

### Preliminaries II: Monadic Second Order Logic and clique-width

#### Monadic Second Order Logic

We assume the reader is familiar with First Order Logic, see, e.g., [23]. Monadic Second Order Logic (or $\textsf {MSO}$) is a powerful logic for graphs and graph-like structures. It is the extension of First Order Logic with set quantification. $\textsf {MSO}$ can define classic graph-theoretic concepts such as planarity, connectivity, *c*-regularity and *c*-colorability. An excellent introduction to $\textsf {MSO}$ is Courcelle and Engelfriet’s book [17], but here we introduce some of the core notions of MSO.

Let $\eta$ be a vocabulary consisting of unary relation symbols $R_{i}$, a binary relation symbol *E* and constant symbols $c_{i}$.

*Syntax*

We define the logic $\textsf {MSO}(\eta )$ inductively. We have two types of variables: first order variables, $x_{i}$ $(i\in \mathbb {N})$ and unary second order variables $U_{i}$ $(i\in \mathbb {N})$. Atomic formulas are of the form $t_{i}=t_{j}$, $E(t_{i},t_{j})$, $R_{i}(t_{j})$, $U_{i}(t_{j})$ where $t_{i},t_{j}$ are first order variables or constant symbols. The logical formulas of $\textsf {MSO}$ are built inductively by using the Boolean connectives $\vee$, $\wedge$, $\lnot$ and $\rightarrow$, and the quantifiers $\forall x_{i}$, $\exists x_{i}$, $\forall U_{i}$, $\exists U_{i}$.

A variable $x_{i}$, $U_{i}$ is *free* if it is not in the scope of an appropriate quantifier. The *quantifier rank* $qr(\varphi )$ of $\varphi \in \textsf {MSO}$ is the maximum number of nested quantifiers.

*Semantics*

Let $\mathcal {M}$ be a structure with universe *M* such that the interpretation of a symbol *R* in $\mathcal {M}$ is $R^{\mathcal {M}}$. Let *m* be a mapping of the free variables to their values: $m(x_{i})$ ranges over *M* and $m(U_{i})$ ranges over subsets of *M*. We extend *m* to $c_{i}$ by setting $m(c_{i})=c_{i}^{\mathcal {M}}$. For the atomic formulas $\varphi _{1}=(t_{i}=t_{j})$, $\varphi _{2}=E(t_{i},t_{j})$, $\varphi _{3}=T_{i}(t_{j})$, $\mathcal {M},m\models \varphi _{j}$, $j=1,2,3$, is defined as in First Order Logic. $\mathcal {M},m\models U_{i}(t_{j})$ is defined as $m(t_{j})\in m(U_{i})$. The semantics of the Boolean connectives $\vee$, $\wedge$, $\lnot$ and $\rightarrow$, and the quantifiers $\exists x_{i}$ is defined as in First Order Logic. We define $\mathcal {M},m\models \exists U_{i}\varphi$ if there exists $X\subseteq M$ such that $\mathcal {M},m_{X}\models \varphi$, where $m_{X}$ is obtained from *m* by setting $m(U_{i})=X$.

Given two $\eta$-structures $\mathcal {M}_1$ and $\mathcal {M}$, we say $\mathcal {M}_1$ and $\mathcal {M}_2$ are $\textsf {MSO}^q$ *-equivalent*, and write $\mathcal {M}_1\equiv _{q}^{\textsf {MSO}}\mathcal {M}_2$, if $\mathcal {M}_1$ and $\mathcal {M}_2$ agree on all $\textsf {MSO}$ sentences of quantifier rank at most *q*.

An (*r*, *w*)-topology *G* is a finite structure over the vocabulary $\left\langle E,T_1,\ldots ,T_r,C_1,\ldots ,C_w\right\rangle$ in which the $T_i$ and $C_i$ are unary, and *E* is binary. An *r*-topology *G* is a finite structure over the vocabulary $\left\langle E,T_1,\ldots ,T_r\right\rangle$. The definition of $\textsf {MSO}^q$-equivalence therefore applies to (*r*, *w*)-topologies and *r*-topologies. Note that using the same notation *E*, $T_i$ and $C_i$ for the symbols appearing in the vocabulary and formulas and for their interpretations in structures ((*r*, *w*)-topologies) is an abuse of notation. However, it should be clear from the context whether we means the symbols or the interpretations.

#### Clique-width

Clique-width is a graph parameter which generalizes the more familiar tree-width. The class of graphs of clique-width at most *w* is defined inductively.

An (*r*, *w*)*-topology* is an expansion $$(V,E,T_1,\ldots ,T_r, C_1,\ldots , C_w)$$ of $$(V,E,T_1,\ldots ,T_r)$$ by a partition $$(C_1, \ldots , C_w)$$ of *V*. For every $$u\in V$$, if $$u\in C_i$$ then we say *u* has *color* *i*. We define the *w*-terms inductively. $$\epsilon $$ is a *w*-term. If *x*, *y* are *w*-terms, then $$ add _{i,t}(x)$$, $$ recol _{i,j}(x)$$, $$ edge _{i,j}(x)$$ and $$x\sqcup y$$ are *w*-terms for $$i,j\in [w]$$, $$t\in [r]$$. Every *w*-term *x* has an associated (*r*, *w*)-topology [[*x*]]:

- $[[\epsilon ]]$ has $V = E = \emptyset$ and empty labeling.
- $[[ add _{i,t} (x)]]$ is formed by adding a new vertex of color *i* and type *t* to [[*x*]].
- $[[ recol _{i,j} (x)]]$ is formed by recoloring every vertex with color *i* of [[*x*]] by *j*.
- $[[ edge _{i,j} (x)]]$ is formed from [[*x*]] by adding an edge from every vertex of color *i* to every vertex of color *j*.
- $[[x \sqcup y]]$ is the disjoint union of *x* and *y* and the union of the labeling.

An *r*-topology *G* has *clique-width at most* *w* if there is a *w*-term $\rho$ such that *G* is isomorphic to $[[\rho (\epsilon )]]$ (forgetting the coloring $C_1,\ldots ,C_w$). Every topology of size *n* has clique-width at most *n*. A class of topologies ${\mathcal G}$ has *bounded clique-width* if there exists *w* such that every graph in ${\mathcal G}$ has clique-width at most *w*.

##### Running Example 4

The topology $G_{re}$ has clique-width at most 3. For $(i,t) \in \{(1,1), (3,2)\}$, let

$$\begin{aligned} \begin{array}{lll} \rho _{i,t} &{}=&{} edge _{2,i}( edge _{i,2}( add _{i,t}( add _{2,2}(\epsilon )))\\ \rho ^{ca} &{}=&{} \rho _{1,1}\\ \rho ^{bd} &{}=&{} \rho _{3,2}\\ \rho _{cabd} &{}=&{} edge _{1,3}( edge _{3,1}(\rho _{ca}\sqcup \rho _{bd})). \end{array} \end{aligned}$$

$\rho ^{ca}$ creates two vertices $u_c$ and $u_a$ such that $u_a$ has color 1 and type 1 and $u_c$ has color 2 and type 2, and adds the edges $(u_a,u_b)$ and $(u_b,u_a)$. $\rho ^{bd}$ creates two vertices $u_b$ and $u_d$ such that $u_b$ has color 3, $u_d$ has color 2, and both vertices have type 2, and adds the edges $(u_d,u_b)$ and $(u_b,u_d)$. $\rho _{cabd}$ adds the edges $(u_a,u_b)$ and $(u_b,u_a)$. $[[\rho _{abcd}]]$ is isomorphic to $G_{re}$ when forgetting the colors.

##### Example 1

**(Cliques)** Let $K_n$ be the 1-topology $G=(V,E,T_1)$ such that $V = T_1 = [n]$ and

$$\begin{aligned} E = \{(i,j) \mid i,j\in [n], i\not =j\}. \end{aligned}$$

Let $$\rho ^{ clq }_0 = \epsilon $$, and

$$\begin{aligned} \rho ^{ clq }_n = recol _{1,2}( edge _{1,2}( edge _{2,1}( add _{1,1} (\rho ^{ clq }_{n-1})))). \end{aligned}$$

We have that $[[\rho ^{ clq }_n]]$ is isomorphic to $K_n$ when ignoring the coloring $C_1,C_2$. Hence, the cliques all have clique-width at most 2. Note that all the elements of $[[\rho ^{ clq }_n]]$ have color 2.

##### Example 2

**(Unidirectional lines)** Let $L_n$ be the 1-topology $G=(V,E,T_1)$ such that $V = T_1 = [n]$ and

$$\begin{aligned} E = \{(i,i+1) \mid 1\le i \le n-1\}. \end{aligned}$$

Let $$\theta _0 = \epsilon $$, and

$$\begin{aligned} \theta _n = recol _{1,2}( recol _{2,3}( edge _{1,2}( add _{1,1} (\theta _{n-1})))). \end{aligned}$$

We have that $[[\theta _n]]$ is isomorphic to $L_n$ when ignoring the coloring. Hence, the unidirectional lines all have clique-width at most 3. Note that all the elements of $[[\theta _n]]$ have color 3, except for *n* which has color 2.

We assume the reader is familiar with tree-width, otherwise see [17] for an introduction to tree-width. The following theorem presents some properties of clique-width (see [17, Propositions 2.106 and 2.114]):

##### Theorem 23

1. All undirected cycles with least four vertices have clique-width 4 and tree-width 2.
2. All cliques with at least two vertices have clique-width 2. On the other hand, the class of cliques has unbounded tree-width.
3. The class of undirected grids has unbounded tree-width and unbounded clique-width.
4. If a class of topologies has bounded tree-width then it has bounded clique-width.

#### Monadic Second Order Logic and clique-width

A parameterized topology ${\mathcal G}$ is $\textsf {MSO}$-*definable* if there exists an $\textsf {MSO}$-formula $\varPhi$ such that $G \in {\mathcal G}$ iff $G \models \varPhi$. For instance, the set of bipartite graphs is defined as:

$$\begin{aligned} \exists U.\, \forall x, y.\, (E(x,y)\rightarrow (U(x)\leftrightarrow \lnot U(y))) \end{aligned}$$

##### Theorem 24

(Courcelle’s Theorem, see [17]) Let $w\ge 1$.

1. The $\textsf {MSO}$ theory of *r*-topologies of clique-width at most *w* is decidable. I.e., on input $\varphi \in \textsf {MSO}$, the problem “is there an *r*-topology of clique-width at most *w* which satisfies $\varphi$” is decidable.
2. For every *q*, the number of equivalence classes in $\equiv _{q}^{\textsf {MSO}}$ is finite.
3. There is a computable function $f:\mathbb {N}^{2}\rightarrow \mathbb {N}$ such that, for every $\psi$, $\psi$ is satisfiable by an *r*-topology of clique-width at most *w* iff it is satisfiable by such a structure of size at most $f(w,qr(\psi ))$. Moreover, $f(w,qr(\psi ))$ can be taken to be a tower of exponents in $w+qr(\psi )$ of height $O(w+qr(\psi ))$.

##### Remark 1

(Tree-width) Tree-width is a well-known graph parameter similar in spirit to clique-width [18, 19]. Every parameterized topology of bounded tree-width also has bounded clique-width, but the converse is not true. If we restrict ourselves to parameterized topologies of bounded tree-width, we may extend $\textsf {MSO}$ by allowing quantification on sets of edges while keeping the decidability.

##### Definition 3

($\textsf {MSO}$ *smoothness,* [45]) A unary operation $\Box$ on (*r*, *w*)-topologies is called $\textsf {MSO}$ *-smooth*, if for all $q\in \mathbb {N}$ whenever $G\equiv _{q}^{\textsf {MSO}}H$, $\Box (G)\equiv _{q}^{\textsf {MSO}}\Box (H)$.

##### Theorem 25

([18]) For any fixed $$i,t,j\in \mathbb {N}$$, the operations $$ add _{i,t}$$, $$ recol _{i,j}$$ and $$ edge _{i,j}$$ are $$\textsf {MSO}$$-smooth.

Indeed, any operation which can be defined as a quantifier-free transduction is $\textsf {MSO}$-smooth (see [45]). Smoothness is also defined for binary operations (e.g., the disjoint union $\sqcup$ is $\textsf {MSO}$-smooth) but we do not use this generality here.

#### Iteratively constructible parameterized topologies

We now introduce a user-friendly and expressive formalism that can be used to generate natural parameterized topologies. A parameterized topology is *iteratively constructible* if it can be built from an initial labeled graph by means of repeating a fixed succession of elementary operations involving addition of vertices and edges, deletion of edges, and relabeling. More precisely, an *r*-ary parameterized topology ${\mathcal G}$ is *iteratively-constructible* if there are *w*-terms $\rho (x),\sigma (x)$ with one variable *x* and no use of disjoint union, and a *w*-graph $H_0$ such that (i) $G \in {\mathcal G}$ iff $G = \sigma (\rho ^n(H_0))$ for some $n \in \mathbb {N}$, where $\rho ^0(H)=H$, (ii) exactly one vertex of $H_0$ has type 1, and (iii) no vertex of type 1 is added in $\rho$ or $\sigma$. For terms $\rho (\cdot )$ and $\rho ^{\prime }(\cdot )$ we write $\rho {:}{:} \rho ^{\prime }$ instead of $\rho (\rho ^{\prime }(\cdot ))$. Intuitively, $\rho$ “builds up” the topology, and $\sigma$ puts on the “finishing touch” (see examples below). The unique vertex of type 1 acts as the initial token position in TPSs. By definition, any parameterized topology has bounded clique-width.

##### Example 3

**(Cliques and rings)** The set of cliques (irreflexive) is iteratively constructible: let $$H_0$$ consist of a single vertex *v* of color 1 and type 1, let $$\rho (x)$$ be $$ edge _{1,1} {:}{:} add _{1,2}(x)$$, and $$\sigma (x)$$ be the identity.

The set of uni-directional rings is iteratively constructible: let $H_0$ consist of two vertices, one of color 1 and type 1 and one of color 2 and type 2 with an edge from 1 to 2. Let $\rho (x)$ be $recol _{4,2} {:}{:} recol _{2,3} {:}{:} edge _{2,4} {:}{:} add _{4,2}$ and $\sigma (x)= edge _{2,1}$.

#### Homogeneous parameterized topologies revisited

All homogeneous parameterized topologies have bounded clique-width and are definable in $\textsf {MSO}$.

##### Proposition 2

Let *H* be a directed graph with vertex set $V_H = [r]$ and edge set $E_H$ and let $B_{sng},B_{clq},B_{ind}$ be a partition of [*r*]. Let ${\mathcal G}$ be the homogeneous parameterized topology defined by *H* and $B_{sng},B_{clq},B_{ind}$. Then:

1. ${\mathcal G}$ has clique-width at most $r + |B_{sng}|$.
2. $${\mathcal G}$$ is $$\textsf {MSO}$$-definable.

##### Proof

First, lets assume for simplicity that we have 2*r* colors. For every function $$ size :[r]\rightarrow \mathbb {N}$$ such that $$ size (i)=1$$ if $$i\in B_{sng}$$, there is a unique topology $$G_ size $$ in $${\mathcal G}$$ such that $$|T_i| = size (i)$$ for all *i*. Conversely, for every topology *G* in $${\mathcal G}$$ there is $$ size $$ such that $$G = G_ size $$.

For every $i\in [r]$, let $\nu ^{[i]}_0 = \epsilon$. For all $i\in B_{ind} \cup B_{sng}$ and $n\in \mathbb {N}$, let $\nu ^{[i]}_n = add _{i,i}(\nu ^{[i]}_{n-1})$. For all $i\in B_{clq}$ and $n\in \mathbb {N}$, let $\nu ^{[i]}_n = recol _{i,r+i}( edge _{i,r+i}( add _{i,i} (\nu ^{[i]}_{n-1})))$. Let $\nu ^{[i]} = \nu ^{[i]}_{ size (i)}$.

Observe that (1) $$[[\nu ^{[i]}]]$$ is a singleton if $$i \in B_{sng}$$, (2) $$[[\nu ^{[i]}]]$$ is an edgeless graph with $$ size (i)$$ vertices if $$i\in B_{ind}$$, and (3) $$[[\nu ^{[i]}]]$$ is a clique with $$ size (i)$$ vertices if $$i\in B_{clq}$$ (see also Example 1).

Let $$\theta _0 = \bigsqcup _{i\in [r]} \nu ^{[i]}$$. The topology $$[[\theta _0]]$$ is the disjoint union of of singletons, independent sets (i.e., edgeless sets), and cliques, whose numbers and sizes are determined by $$B_{sng},B_{clq},B_{ind}$$ and *size*. We denote $$\theta _0^ size = \theta _0$$ when we would like to make $$ size $$ explicit in the notation. It remains to add the edges between the vertices in different $$T_i$$’s. Let $$E_H = \{e_1,\ldots ,e_m\}$$. For every $$e_n = (i,j) \in E_H$$, let $$\theta _n = edge _{i,j}(\theta _{n-1})$$. We have that $$[[\theta _m]]$$ is isomorphic to $$G_ size $$.

Finally we note that a color $r+i> r$ was only used if $i \in B_{clq}$. Hence, only $r + |B_{sng}|$ colors are really used, and $G_{ size }$ has clique-width at most $r + |B_{sng}|$.

Now we turn to $\textsf {MSO}$-definability. We need a few auxiliary sentences:

$$\begin{aligned} \begin{array}{lll} \mathrm {ind}_i &{} = &{} \forall x,y.\, \big ((T_i(x)\wedge T_i(y))\rightarrow (\lnot E(x,y))\big )\\ \mathrm {clq}_i &{} = &{} \forall x,y.\, \big ((T_i(x)\wedge T_i(y))\rightarrow ((i\not =j)\leftrightarrow E(x,y))\big )\\ \mathrm {sng}_i &{} = &{} \big (\exists x.\,T_i(x)\big ) \wedge \big (\forall x,y.\,((T_i(x)\wedge T_i(y))\rightarrow (x=y))\big )\\ \end{array} \end{aligned}$$

A topology *G* satisfies $\mathrm {ind}_i$ iff $T_i$ induces an edgeless graph, *G* satisfies $\mathrm {clq}_i$ iff $T_i$ induces a clique, and *G* satisfies $\mathrm {sng}_i$ iff $T_i$ is a singleton. For every $i \in [r]$, let $\phi _i = \mathrm {ind}_i$, $\phi _i = \mathrm {clq}_i$, and $\phi _i = \mathrm {sng}_i$ if, respectively, $i\in B_{ind}$, $i\in B_{clq}$, or $i\in B_{sng}$. For every $i,j\in [r]$, $i\not =j$, let

$$\begin{aligned} \psi _{(i,j)} = \forall x,y.\, \big ((T_i(x) \wedge T_j(y)) \rightarrow MaybeEdge _{i,j}(x,y)\big ) \end{aligned}$$

where $$ MaybeEdge _{i,j}(x,y) = E(x,y)$$ if $$(i,j)\in E_H$$, and otherwise $$ MaybeEdge _{i,j}(x,y) = \lnot E(x,y)$$. Hence *G* satisfies the following sentence $$\phi _H$$ iff there is $$ size $$ such that *G* is isomorphic to $$G_ size $$:

$$\begin{aligned} \phi _H = \bigwedge _{i\in [r]} \phi _i \wedge \bigwedge _{i,j\in [r], i\not =j} \psi _{(i,j)}. \end{aligned}$$

### Decidability of PMCP

#### $\textsf {MSO}$-definable topologies of bounded clique-width

The purpose of this subsection is to prove the following theorem.

##### Theorem 26

Let ${\mathcal {P}}$ be the set of token-passing system templates. Let ${\mathcal G}$ be a parameterized topology that is $\textsf {MSO}$-definable and contains only topologies of clique-width at most $w\in \mathbb {N}$. Then

1. The problem ${{\textsf {PMCP}}}_{{\mathcal G}}({\mathcal {P}},{\textsf {i-CTL}}^*\backslash \textsf {X})$ is decidable;
2. There is an algorithm that given *k* and *d* produces a cutoff $F({\mathcal G},k,d)$ for ${\textsf {PMCP}}_{\mathcal G}({\mathcal {P}},\text{ k- }\textsf {CTL}^*_d\backslash \textsf {X})$.

We give an outline of the proof before diving into the details:

(I) We give an alternative definition of the marking function $\varXi$ with simple (i.e., cycle-free) paths rather than any paths.
(II) We show that the $$\text{ k- }\textsf {CTL}^*_d\backslash \textsf {X}$$-character of the graph LTS $${{G}}|{\bar{g}}$$ is $$\textsf {MSO}$$-definable. That is, for every member $$\mathfrak {C}$$ of $$\mathrm {Char}^k_d$$, we construct an $$\textsf {MSO}$$-formula $$ chr _\mathfrak {C}$$ such that $${{G}}|{\bar{g}}$$ has $$\text{ k- }\textsf {CTL}^*_d\backslash \textsf {X}$$-character $$\mathfrak {C}$$ if and only if *G*, $$\bar{g}$$, and $$ init _G$$ satisfy $$ chr _\mathfrak {C}$$. To do so, we use the alternative definition of $$\varXi $$ with simple paths.
(III) We show how to compute a mapping $$ rep ^k_{d,w}$$ assigning to every $$\text{ k- }\textsf {CTL}^*_d\backslash \textsf {X}$$-character $$\mathfrak {C}\in \mathrm {Char}^k_d$$ a *representative* $${{H}}|{\bar{h}}$$. The representative $${{H}}|{\bar{h}}$$ is a graph LTS such that *H* has clique-width at most *w*. To do so, we use the $$\textsf {MSO}$$-definability of $$\text{ k- }\textsf {CTL}^*_d\backslash \textsf {X}$$-characters and the decidability of $$\textsf {MSO}$$ on topologies of bounded clique-width.
(IV) We show how to reduce the problem of whether a token-passing system with topology *G* satisfies a $\text{ k- }\textsf {CTL}^*_d\backslash \textsf {X}$-sentence to the problem of whether *G* satisfies an $\textsf {MSO}$-sentence. To do so, we use the composition theorem, Theorem 21, as well as the mapping of $\text{ k- }\textsf {CTL}^*_d\backslash \textsf {X}$-characters to representatives, and the $\textsf {MSO}$-definability of $\text{ k- }\textsf {CTL}^*_d\backslash \textsf {X}$-characters.
(V) Finally, using the reduction from the previous item and the $\textsf {MSO}$-definability of ${\mathcal G}$, we express the PMCP as a satisfiability problem of a certain $\textsf {MSO}$-sentence. We use that ${\mathcal G}$ has bounded clique-width and that deciding $\textsf {MSO}$-satisfiability over bounded clique-width is decidable. The cutoff is then obtained from the bound on the size of the minimal satisfying model of an $\textsf {MSO}$-sentence.

**(I) Defining markings using simple paths**

Let ${{G}}|{\bar{g}} = (V,\varDelta ,V_{0},\varLambda )$ be a graph LTS for an *r*-topology $G=(V,E,\bar{T})$. First, we can move to finite paths instead of infinite paths:

##### Lemma 6

Let $\pi = (v_1,v_2,\ldots )$ be an infinite path. Let $j\in \mathbb {N}$ be such that $v_j=v_i$ for infinitely many $i\in \mathbb {N}$. Let $\pi _0 = (v_1,v_2,\ldots ,v_j)$ be the path obtained from $\pi$ by cutting the path $\pi$ at $v_j$. For every $d,k \in \mathbb {N}$, $\varXi ^k_d(\pi ) = \varXi ^k_d(\pi _{0})$.

##### Proof

For every *i* such that $v_j=v_i$, $\varXi ^k_d(v_j)=\varXi ^k_d(v_i)$. By Lemma 5, $\mathsf {destutter}(\varXi ^k_d(\pi ))$ is strictly decreasing in the poset $(\varUpsilon ^k_{d-1}, \preceq ^k_{d-1})$. This implies that $\varXi ^k_d(\pi )$ is non-increasing, and, using that there are infinitely many *i* such that $v_j=v_i$, for every $\ell >j$, $\varXi ^k_d(v_j)=\varXi ^k_d(v_\ell )$. Hence, $\mathsf {destutter}(\varXi ^k_d(v_j, v_{j+1},\ldots )) = \mathsf {destutter}(\varXi ^k_d(v_j))$. $\square$

Second, we can move to simple finite paths instead of finite paths (by repeated application of the following lemma):

##### Lemma 7

Let $\pi = (v_1,v_2,\ldots )$ be a (finite or infinite) path with a cycle, i.e., there are $i < j$ such that $v_i = v_j$. Let $\pi _{1}$ be the path obtained from $\pi$ by removing the subpath $(v_{i+1},\ldots ,v_{j})$ from $\pi$. For every $d,k \in \mathbb {N}$, $\varXi ^k_d(\pi ) = \varXi ^k_d(\pi _{1})$.

##### Proof

Since $v_i=v_j$ we have that $\varXi ^k_d(v_j)=\varXi ^k_d(v_i)$. By Lemma 5, $\mathsf {destutter}(\varXi ^k_d(\pi ))$ is strictly decreasing in the poset $(\varUpsilon ^k_{d-1}, \preceq ^k_{d-1})$, implying that $\varXi ^k_d(\pi )$ is non-increasing. Hence, for every $i\le \ell \le j$, $\varXi ^k_d(v_\ell )=\varXi ^k_d(v_j)$, implying that: $\mathsf {destutter}(\varXi ^k_d(v_i,\ldots , v_{j})) = \mathsf {destutter}(\varXi ^k_d(v_i))$. $\square$

For every $j\in \{0,\ldots ,k\}$, and every vertex $v\in V$, let $v^{\curvearrowright j}$ be the set of simple finite paths $\pi$ in *G* which start at *v*, have no vertices in $\bar{g}$, and whose last vertex *u* has an edge to $g_j$ if $j>0$, or to some vertex in $\pi$ if $j=0$. Lemmas 6 and 7, we have:

##### Lemma 8

Let $k,d\in \mathbb {N}$ with $d>0$. Let ${{G}}|{\bar{g}}$ be a graph LTS. For every $j \in \{0,\ldots ,k\}$, we have:

1 $$\begin{aligned} \begin{array}{ll} \displaystyle {\varXi ^k_d(v)[j] = \bigcup _{\pi \in v^{\curvearrowright j}} \{\mathsf {destutter}(\varXi ^k_{d-1}(\pi ))\}} \end{array} \end{aligned}$$

##### Running Example 5

For ${{G_{re}}}|{\bar{g}}$ with $\bar{g}=(g_1,g_2)$, $g_1 = b$, and $g_2 = d$, we have:

$$\begin{aligned} \begin{array}{lllllllllll} a^{\curvearrowright 0} &{}=&{} \{ac\} &{} &{} a^{\curvearrowright 1} &{}=&{} \{a\} &{} &{} a^{\curvearrowright 2} &{}=&{} \emptyset \\ b^{\curvearrowright 0} &{}=&{} \{bac\} &{} &{} b^{\curvearrowright 1} &{}=&{} \{ba\} &{} &{} b^{\curvearrowright 2} &{}=&{} \{b\}\\ c^{\curvearrowright 0} &{}=&{} \{ca\} &{} &{} c^{\curvearrowright 1} &{}=&{} \{ca\} &{} &{} c^{\curvearrowright 2} &{}=&{} \emptyset \\ d^{\curvearrowright 0} &{}=&{} \emptyset &{} &{} d^{\curvearrowright 1} &{}=&{} \{d\} &{} &{} d^{\curvearrowright 2} &{}=&{} \emptyset \end{array} \end{aligned}$$

The reader can verify that applying Equation (1) of Lemma 8 gives the same values of $\varXi ^k_d(v)[j]$ as those computed in Running Example 3.

**(II)** $\text{ k- }\textsf {CTL}^*_d\backslash \textsf {X}$ **-character is** $\textsf {MSO}$ **-definable**

##### Proposition 3

(The marking $\varXi ^k_d$ of a graph LTS is $\textsf {MSO}$-definable) Let $d,k \in \mathbb {N}$ and $\mathfrak {a} \in \varUpsilon ^k_d$. There is an $\textsf {MSO}$-formula $mark _\mathfrak {a}$ with $k+1$ free first-order variables such that, for every *r*-topology $G = (V,E,\bar{T})$ and $v,g_1\ldots ,g_k\in V$, $G \models mark _\mathfrak {a}(v,\bar{g})$ iff $\varXi ^k_d(v) = \mathfrak {a}$, where $\varXi ^k_d$ is the marking of ${{G}}|{\bar{g}}$.

##### Proof

We will prove this proposition by induction on *d*.

Let $d = 0$. By Lemma 5, $\varUpsilon ^k_0$ consists of subsets of $\{p_1,\ldots ,p_k\}$. The desired $mark _{\mathfrak {a}}(x,w_1,\ldots ,w_k)$ is:

$$\begin{aligned} \begin{array}{lll} mark _{\mathfrak {a}}(x)= & {} \displaystyle \bigwedge _{i:\,p_i\in \mathfrak {a}} (x = w_i) \wedge \bigwedge _{i:\,p_i\notin \mathfrak {a}} \lnot (x = w_i) \end{array} \end{aligned}$$

since: $G \models mark _\mathfrak {a}(v,\bar{g})$ iff $\{i \mid v = g_i\} = \{i \mid p_i \in \mathfrak {a}\}$ iff $\varLambda (v) = \mathfrak {a}$ iff $\varXi ^k_0(v) = \varLambda (v)$.

Let $d>0$ and $\mathfrak {a}\in \varUpsilon ^k_d$. For every $\mathfrak {b} \in \varUpsilon ^k_{d-1}$, let $mark _{\mathfrak {b}}$ be the $\textsf {MSO}$-formula guaranteed by the induction hypothesis for $\mathfrak {b}$.

Let $$ chains ^k_{d-1}$$ denote the set of strictly decreasing chains of elements in $$(\varUpsilon ^k_{d-1},\preceq ^k_{d-1})$$. By Lemma 5, $$\mathfrak {a} = (\mathfrak {A}_0,\ldots ,\mathfrak {A}_k)$$, where each $$\mathfrak {A}_i \subseteq chains ^k_{d-1}$$.

We will use the definition of $$\varXi ^k_d$$ in Lemma 8 to define $$ mark _\mathfrak {a}$$. The formula $$ mark _\mathfrak {a}(x,w_1,\ldots ,w_k)$$ is the conjunction $$\bigwedge _{i=0}^k mark - coord _i$$, where $$ mark - coord _i$$ will be defined below to guarantee that the *i*th coordinate $$\mathfrak {A}_i$$ of the vector $$\mathfrak {a}$$ follows the definition of $$\varXi ^k_d[i]$$ in Lemma 8.

By the definition of $$\varXi ^k_d[j]$$ in Lemma 8, $$\varXi ^k_d$$ consists of the set of $$ chains ^k_{d-1}$$ elements $$ ch $$ for which there exists a path $$\pi $$ in $$v^{\curvearrowright j}$$ such that $$\mathsf {destutter}(\varXi ^k_d(\pi )) = ch $$. For every $$ ch \in chains ^k_{d-1}$$, we will define below $$\theta _ ch $$ to express that there exists a path $$\pi $$ in $$v^{\curvearrowright j}$$ such that $$\mathsf {destutter}(\varXi ^k_d(\pi )) = ch $$. Using the $$\theta _ ch $$, we can define $$ mark - coord _j$$ as follows:

$$\begin{aligned} mark - coord _j = \bigwedge _{ ch \in \mathfrak {A}_j} \theta _ ch \wedge \bigwedge _{ ch \in chains ^k_{d-1} {\setminus } \mathfrak {A}_j} \lnot \theta _ ch \end{aligned}$$

To define $$\theta _ ch $$, observe that for a path $$\pi =v_1,\ldots ,v_r$$ and a chain $$ch= ch _1,\ldots , ch _s$$ in $$ chains ^k_{d-1}$$, the following are equivalent:

(i) $$\mathsf {destutter}(\varXi ^k_d(\pi )) = ch $$.
(ii) There are $1=f_0\le f_1<\ldots <f_s= r$ such that $\varXi ^k_d(v_{j}) = ch _i$ for all $f_{i-1}\le j \le f_i$.

Hence, for a chain $$ch= ch _1,\ldots , ch _s$$ in $$ chains ^k_{d-1}$$, the following are equivalent:

(i) There is $\pi$ in $v^{\curvearrowright j}$ such that $\mathsf {destutter}(\varXi ^k_d(\pi )) = ch$.
(ii) There are simple finite paths $\pi _1,\ldots ,\pi _s$ which are consecutive (i.e., the last vertex of $\pi _{i}$ is adjacent to the first vertex of $\pi _{i+1}$ for all *i*) such that if $v\in \pi _i$ then $\varXi ^k_{d-1}(v) = ch _i$. Let the last vertex of $\pi _s$ be *u*. If $j>0$ then *u* has an edge to $u^{\prime } = g_j$. If $j=0$ then *u* has an edge to some $u^{\prime }$ in one of $\pi _1\ldots ,\pi _s$.

For every $\mathfrak {b} \in \varUpsilon ^k_{d-1}$, $reach _\mathfrak {b}(y,y^{\prime })$ below expresses that there is a path $z_1,\ldots ,z_t$, $t\in \mathbb {N}$, starting from $z_1 = y$ and ending at $z_t = y^{\prime }$ such that $\varXi ^k_{d-1}(z_i) = \mathfrak {b}$ for all *i*:

$$\begin{aligned} \begin{array}{lll} reach _\mathfrak {b}(y,y^{\prime }) &{}=&{} \exists Z.\,\forall z.\,(Z(z)\rightarrow mark _\mathfrak {b}(z))\ \wedge \\ &{}&{} reach (Z,y,y^{\prime })\\ reach (Z,y,y^{\prime }) &{}=&{} \forall Y.\,\big (( subset (Y,Z)\wedge Y(y)\wedge \lnot Y(y^{\prime })) \\ &{} &{} \rightarrow (\exists z_1.\,\exists z_2.\,Y(z_1) \wedge \\ &{} &{} \lnot Y(z_2) \wedge E(z_1,z_2))\big )\\ subset (Y,Z) &{}=&{} \forall y.\,(Y(y)\rightarrow Z(y)) \end{array} \end{aligned}$$

where $reach (Z,y,y^{\prime })$ expresses that there is a path between *y* and $y^{\prime }$ in the subgraph induced by *Z* similarly to the classical definition of connectivity in $\textsf {MSO}$ e.g., in [44, Proposition 7.14].

Finally, $\theta _{ch}$ is given by

$$\begin{aligned} \begin{array}{ll} \exists y_1.\,\exists y_1^{\prime }\ldots \exists y_s.\, \exists y_s^{\prime }.\, \\ \left( last _j\wedge \bigwedge _{i=1}^s reach _{ ch _i}(y_i,y_i^{\prime }) \wedge \bigwedge _{i=1}^{s-1} E(y_i^{\prime },y_{i+1}) \right) \\ \end{array} \end{aligned}$$

where $$ last _j$$ expresses that the last vertex $$y_s^{\prime }$$ of $$\pi _s$$ has an edge to $$ g_j$$ if $$j>0$$, or to some vertex of $$\pi _1\ldots ,\pi _s$$ if $$j=0$$:

$$\begin{aligned} \begin{array}{lll} last _0(\bar{y},\bar{y^{\prime }}) &{}=&{} \displaystyle \bigvee _{p \in \{y_1, y_1^{\prime },\ldots ,y_s,y_s^{\prime }\}} E(y_s^{\prime },p)\\ last _j(y_s^{\prime },w_j) &{}=&{} E(y_s^{\prime },w_j)\\ \end{array} \end{aligned}$$

for $$j\in [k]$$. $$\square $$

##### Proposition 4

(The $\textsf {CTL}^*_{d}\backslash \textsf {X}$-character of a graph LTS is $\textsf {MSO}$-definable) Let $d,k \in \mathbb {N}$ and $\mathfrak {C} \in \mathrm {Char^d_k}$. There is an $\textsf {MSO}$-formula $chr _\mathfrak {C}$ with *k* free first-order variables such that, for every *r*-topology $G = (V,E,\bar{T})$ with $T_1=\{init\}$ and $g_1\ldots ,g_k\in V$, $G \models chr _\mathfrak {C}(init,\bar{g})$ iff the $\textsf {CTL}^*_{d}\backslash \textsf {X}$-character of ${{G}}|{\bar{g}}$ is $\mathfrak {C}$.

##### Proof

Let $init$ be the unique vertex in $T_1$. Let $\mathfrak {C} = (\mathfrak {a}_{ init }, \mathfrak {b}_{ init }, \mathfrak {a}_1,\mathfrak {b}_1,\ldots ,\mathfrak {a}_k,\mathfrak {b}_k)$. By the definition of $\textsf {CTL}^*_{d}\backslash \textsf {X}$-character, the graph LTS ${{G}}|{\bar{g}}$ has character $\mathfrak {C}$ iff for every *v* of $init ,g_1,\ldots ,g_k$, $\varXi ^k_0(v)=\mathfrak {a}_v$ and $\varXi ^k_d(v)= \mathfrak {b}_v$. We use here that $\varXi ^k_0(v)=\varLambda (v)$. Using the formulas of the form $mark _{\mathfrak {a}}$ guaranteed in Proposition 3, ${{G}}|{\bar{g}}$ has $\textsf {CTL}^*_{d}\backslash \textsf {X}$-character $\mathfrak {C}$ iff $G \models chr _\mathfrak {C}$, where $chr _\mathfrak {C}(\bar{w})$ is

$$\begin{aligned} \begin{array}{l} \exists x. \, (T_1(x)\wedge mark _{\mathfrak {a}_{ init }}(x,\bar{w})\wedge mark _{\mathfrak {b}_{ init }}(x,\bar{w}))\\ \quad \wedge \displaystyle \bigwedge _{i=1}^k mark _{\mathfrak {a}_{i}}(w_i,\bar{w})\wedge mark _{\mathfrak {b}_{i}}(w_i,\bar{w}) \end{array} \end{aligned}$$

and $\bar{w}=(w_1,\ldots ,w_k)$ is a tuple of first-order variables. Observe that we quantify over *x* to obtain the vertex *init* which starts with the token; *init* is the unique vertex belonging to $T_1$. $\square$

**(III) The representative mapping**

##### Lemma 9

(Computable representatives) Assume $$k,d,w\in \mathbb {N}$$. There is a computable function $$ rep = rep ^k_{d,w}$$ which maps every $$\mathfrak {C}\in Char ^k_d$$ either to a graph LTS $${{H}}|{\bar{h}}$$ or to $$\bot $$. If $$ rep (\mathfrak {C})={{H}}|{\bar{h}}$$, then *H* has clique-width at most *w* and the $$\textsf {CTL}^*_{d}\backslash \textsf {X}$$-character of $${{H}}|{\bar{h}}$$ is $$\mathfrak {C}$$. If $$ rep (\mathfrak {C})=\bot $$, then there is no $${{H}}|{\bar{h}}$$ such that *H* has clique-width as most *w* and whose $$\textsf {CTL}^*_{d}\backslash \textsf {X}$$-character is $$\mathfrak {C}$$.

##### Proof

Let $\mathfrak {C} \in \mathrm {Char}^k_d$. By Proposition 4, there does not exist a graph LTS ${{G}}|{\bar{g}}$ whose $\textsf {CTL}^*_{d}\backslash \textsf {X}$-character is $\mathfrak {C}$ iff the sentence

$$\begin{aligned} unfeasible _\mathfrak {C} = \lnot \exists w_1 \ldots \exists w_k.\, chr _\mathfrak {C}(w_1,\ldots ,w_k) \end{aligned}$$

belongs to the $\textsf {MSO}$ theory of topologies of clique-width at most *w*. By Theorem 24, the $\textsf {MSO}$ theory of topologies of clique-width at most *w* is decidable. If $unfeasible _\mathfrak {C}$ is valid for topologies of clique-width at most *k*, then $rep (\mathfrak {C}) = \bot$. Otherwise, we search for ${{H}}|{\bar{h}}$ whose character is $\mathfrak {C}$ by iteratively checking all graphs $H=[[t]]$ and *k*-tuples of their elements $\bar{h}$, where in the *i*th stage of the iteration, *t* iterates over all *w*-terms *t* of size at most *i*. When we find ${{H}}|{\bar{h}}$ whose $\textsf {CTL}^*_{d}\backslash \textsf {X}$-character is $\mathfrak {C}$, as is guaranteed to occur, we set $rep (\mathfrak {C})$ to ${{H}}|{\bar{h}}$ and end the search. $\square$

**(IV) Reduction from** $\text{ k- }\textsf {CTL}^*_d\backslash \textsf {X}$ **on token-passing systems to** $\textsf {MSO}$ **on topologies**

Consider a $\text{ k- }\textsf {CTL}^*_d\backslash \textsf {X}$-formula $\psi$ for which we want to verify that, for every $G\in {\mathcal G}$, $\overline{P}^G\models \psi$. We show in Lemma 10 that there is an $\textsf {MSO}$-sentence $\alpha _\psi$ such that $\alpha _\psi$ is satisfied by $G\in {\mathcal G}$ iff the system $\overline{P}^G$ satisfies $\psi$. The formula $\psi$ is of the form $Q_{1}x_{1}\ldots Q_{k}x_{k}.\, \varphi (\bar{x})$, where $\varphi$ is a $\textsf {CTL}^*_{d}\backslash \textsf {X}$-formula. We construct $\alpha _\psi$ to have the form $\alpha _\psi = Q_{1}x_{1}\ldots Q_{k}x_{k}.\beta _\psi$, where $\beta _\psi$ is an $\textsf {MSO}$-formula. To build $\beta _\psi$ we use the *composition* property of $\text{ k- }\textsf {CTL}^*_d\backslash \textsf {X}$ from Theorem 21. Coupled with Theorem 22, the composition property says that the token-passing systems ${{\overline{P}^G}}|{\bar{g}}$ and ${{\overline{P}^H}}|{\bar{h}}$ have the same $\textsf {CTL}^*_{d}\backslash \textsf {X}$-character if their graph LTSs have the same $\textsf {CTL}^*_{d}\backslash \textsf {X}$-character.

##### Lemma 10

For every formula

$$\begin{aligned} \psi =Q_{1}x_{1}\ldots Q_{k}x_{k}.\ \varphi (\bar{x}) \in \text{ k- }\textsf {CTL}^*_d\backslash \textsf {X}, \end{aligned}$$

there exists a computable $\alpha _{\psi }\in \textsf {MSO}$ such that for every *G* with clique-width at most *w*,

$$\begin{aligned} \overline{P}^{G}\models \psi \text{ if } \text{ and } \text{ only } \text{ if } G\models \alpha _{\psi }. \end{aligned}$$

##### Proof

Let $G\in {\mathcal G}$ be a topology and $\overline{P}\in {\mathcal {P}}$ be a system template.

$$\overline{P}^{G}\models Q_{1}x_{1}\ldots Q_{k}x_{k}. \varphi $$

$$\iff Q_{1}g_{1}\in V_{G}\ldots Q_{k}g_{k}\in V_{G}:\overline{P}^{G}\models \varphi [p_{x_{j}}{\mapsto } p_{g_{j}}]$$

$${\iff } Q_{1}g_{1}\in V_{G}\ldots Q_{k}g_{k}\in V_{G}:{{\overline{P}^{G}}}|{\bar{g}}\models \varphi [p_{x_{j}}{\mapsto } p_{g_{j}}]$$

$${\iff } Q_{1}g_{1}{\in } V_{G}\ldots Q_{k}g_{k}{\in } V_{G}: \text{ for } \text{ all } \mathfrak {C}\in \mathrm {Char^d_k} \text{ and } $$

$$ rep ^k_{d,w}(\mathfrak {C})={{H}}|{\bar{h}}, \text{ if } \text{ the } \textsf {CTL}^*_{d}\backslash \textsf {X}\text{-character } \text{ of } {{G}}|{\bar{g}}$$

$$ \text{ is } \mathfrak {C}, \text{ then } {{\overline{P}^{H}}}|{\bar{h}}\models \varphi [p_{x_{j}}\mapsto p_{g_{j}}]$$

$$\iff G\models \alpha _{\psi }$$, where $$\alpha _\psi $$ is

$${\displaystyle Q_1 x_{1} \ldots Q_k x_{k}\bigvee _{\begin{array}{c} \mathfrak {C}\in \mathrm {Char}^k_d: rep ^k_{d,w}(\mathfrak {C})={{H}}|{\bar{h}} \\ \text{ and } {{\overline{P}^{H}}}|{\bar{h}}\models \varphi [p_{x_{j}}\mapsto p_{g_{j}}] \end{array}}} chr _\mathfrak {C}.$$

The disjunction in the last formula is over all elements $$\mathfrak {C}$$ of $$\mathrm {Char}^k_d$$ which are the $$\textsf {CTL}^*_{d}\backslash \textsf {X}$$-characters of some $${{H}}|{\bar{h}}$$ such that $${{\overline{P}^H}}|{\bar{h} \models \varphi [p_{x_{j}}\mapsto p_{g_{j}}]}$$. Here we denote by $$\varphi [p_{x_{j}}\mapsto p_{g_{j}}]$$ the formula that results from replacing every atom in $$\varphi $$ of the form $$p_{x_{j}}$$ by the atom $$p_{g_{j}}$$, for $$p\in \textsf {AP}_{\textsf {pr}}$$ and $$1\le j\le k$$. The first equivalence is by the definition of semantics of indexed temporal logic; the second is by the definition of $${{P^{G}}}|{\bar{g}}$$; the third is by Theorem 21 and by the definition of $$ rep ^{k}_{d,w}$$ in Lemma 9; the fourth is by Proposition 4.

The formula $$\alpha _{\psi }$$ is computable because $$ rep ^k_{d,w}$$ is computable (Lemma 9), $$ chr _\mathfrak {C}$$ is computable, for each $$\mathfrak {C}$$ in $$\mathrm {Char}^k_d$$ (Proposition 4), and model checking whether $${{P^{H}}}|{\bar{h}}\models \psi [p_{x_{j}}\mapsto p_{g_{j}}]$$ is computable. $$\square $$

**(V) Decidability of PMCP**

##### Proof

(*T*heorem 26) Let $\varPhi$ be the $\textsf {MSO}$ formula defining ${\mathcal G}$. By Lemma 10, there is $G\in {\mathcal G}$ such that $\overline{P}^{G}\not \models \psi \text{ if } \text{ and } \text{ only } \text{ if }$ there is $G\in {\mathcal G}$ such that $G\models \varPhi \wedge \lnot \alpha _{\psi }$. By Theorem 24, there is $G\in {\mathcal G}$ such that $G\models \varPhi \wedge \lnot \alpha _{\psi }$ iff there exists such *G* of size at most $f(w,|\varphi |)$, so $f(w,|\varphi |)$ is a cutoff for the problem. $\square$

##### Remark 2

(Fairness) The conference version of this paper [3] assumed that token-passing systems satisfy a fairness condition, namely that the token visits every process infinitely often. In contrast, the presentation in this paper does not require this fairness condition to hold. Elimination of the fairness condition is the reason that we replaced the treatment of $\equiv _{\textsf {CTL}^*_{d}\backslash \textsf {X}}$ equivalence classes using *contractions* based on [2] in [3] with a treatment using $\textsf {CTL}^*_{d}\backslash \textsf {X}$-characters and markings based on [6] in the current paper.

Combining Theorem 26 with Proposition 1 we get:

##### Corollary 3

Let ${\mathcal G}$ be a parameterized topology that is $\textsf {MSO}$-definable and contains only topologies of clique-width at most $w\in \mathbb {N}$. Let ${\mathcal F}$ be the set of 1-index $\textsf {LTL}\backslash \textsf {X}$ formulas, and let $\overline{P}$ be an *r*-ary system template. Then the set of executions $\textsc {1-exec}_{{\mathcal G}}{(\overline{P})}$ is $\omega$-regular.

Using Theorem 26 and Proposition 2 we have:

##### Corollary 4

Let ${\mathcal {P}}$ be the set of token-passing system templates. Let ${\mathcal G}$ be a homogeneous parameterized topology. Then

1. The problem ${{\textsf {PMCP}}}_{{\mathcal G}}({\mathcal {P}},{\textsf {i-CTL}}^*\backslash \textsf {X})$ is decidable;
2. There is an algorithm that given *k* and *d* produces a cutoff $F({\mathcal G},k,d)$ for ${\textsf {PMCP}}_{\mathcal G}({\mathcal {P}},\text{ k- }\textsf {CTL}^*_d\backslash \textsf {X})$.

#### Iteratively-constructible parameterized topologies

The decidability of the PMCP problem for iteratively-constructible parameterized topologies can be reduced to decidability of $\textsf {MSO}$ in the presence of an auxiliary order relation (see for instance the discussion of the iteratively constructible class $\mathrm {EQCLIQUE}$ of graphs consisting of two cliques of equal size in [33, Example 1(ix)]). However, another approach to the decidability of the PMCP problem of iteratively-constructible parameterized topologies via $\textsf {MSO}$ will be easier.

##### Theorem 27

Let ${\mathcal {P}}$ be the set of token-passing system templates. For every iteratively-constructible ${\mathcal G}$,

1. $${{\textsf {PMCP}}}_{{\mathcal G}}({\mathcal {P}},{\textsf {i-CTL}}^*\backslash \textsf {X})$$ is decidable;
2. There is an algorithm that given *k* and *d* produces a cutoff for $\text{ k- }\textsf {CTL}^*_d\backslash \textsf {X}$.

##### Proof

Let $\psi \in {\textsf {i-CTL}}^*\backslash \textsf {X}$. Let $\sigma (x)$ and $\rho (x)$ be *w*-terms and $G_{n}=[[\sigma (\rho ^{n}(\epsilon ))]]$. Since there are finitely many equivalence classes of $\equiv _{q}^{\textsf {MSO}}$, there are $n_{1}<n_{2}\in \mathbb {N}$ such that $[[\rho ^{n_{1}}(\epsilon )]]\equiv _{q}^{\textsf {MSO}}[[\rho ^{n_{2}}(\epsilon )]]$. By Theorem 25, applying the same sequence of clique-width operations on $\equiv _{q}^{\textsf {MSO}}$-equivalent topologies leads again to $\equiv _{q}^{\textsf {MSO}}$-equivalent topologies, i.e., $[[\sigma (\rho ^{n_{1}+e}(\epsilon ))]]\equiv _{q}^{\textsf {MSO}}[[\sigma (\rho ^{n_{2}+e}(\epsilon ))]]$ for every $e\in \mathbb {N}$. Therefore, for every $\varphi \in \textsf {MSO}$, $G_{n}\models \varphi$ for every *n* iff $G_{n}\models \varphi$ for every $n<n_{2}$. Let $\alpha _{\psi }\in \textsf {MSO}$ be the formula guaranteed by Lemma 10 such that for every $G\in {\mathcal G}$, $\overline{P}^{G}\models \psi \text{ if } \text{ and } \text{ only } \text{ if } G\models \alpha _{\psi }$ and $\alpha _{\psi }$ is computable. We get that for every $G\in {\mathcal G}$, $\overline{P}^{G}\models \psi$ iff $G_{n}\models \alpha _{\psi }$ for every $n<n_{2}$. $\square$

**Fig. 11:**
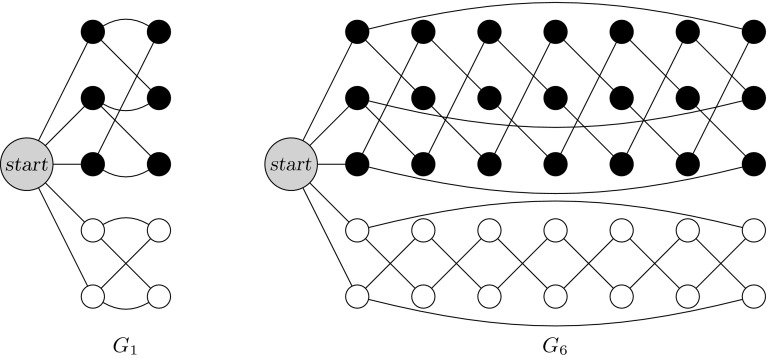
The figure depicts $G_1$ and $G_6$ for *k* in the range $3(pr_1+pr_2)+1=16\le k<3(pr_1+pr_2+pr_3)+1=31$ (where $pr_1 = 2, pr_2 = 3, pr_3 =5$). For such *k*, we have $t=2$, $k^{\prime } = pr_1+pr_2=5$. The *white* vertices of $G_{n}$ are $\{1\}\times [pr_{1}]\times \{0,\ldots ,n\}$. The *black* vertices of $G_{n}$ are $\{2\}\times [pr_{2}]\times \{0,\ldots ,n\}$. The clique-width of $G_n$ is at most $3k^{\prime }+1=16$. The *black* vertices of $G_{6}$ induce three disjoint cycles and $G_{6}\not \models \varphi =\lnot \alpha$, whilst the *black* vertices of $G_{1}$ induce one cycle. We have $G_{n}\models \varphi =\lnot \alpha$ for all $n< \prod _{i\le t}pr_i = 2\cdot 3 = 6$

### Cutoffs

The algorithms obtained from the proofs of Theorem 26 and Theorem 27 give non-elementary upper bounds on the cutoffs due to the number of equivalence classes in $\equiv _{q}^{\textsf {MSO}}$. It is well-known that the latter also has a non-elementary lower bound (this is true already for first order logic, see [35, Lemma 10.21]). Therefore, in order to find low cutoffs, one must look at formalisms which are inherently simpler than $\textsf {MSO}$ such as iteratively-constructible parameterized topologies. We believe that a more direct proof of decidability of PMCP for iteratively-constructible parameterized topologies will give rise to elementary cutoffs close to the following lower bound:

#### Theorem 28

There exists a 2-ary system template $\overline{P}$, and, for every $k\in \mathbb {N}$, an iteratively constructible parameterized 2-topology ${\mathcal G}$ of clique-width at most *k* and a *k*-indexed $\textsf {LTL}\backslash \textsf {X}$ formula^12^ $\varphi$ such that the smallest cutoff for ${\textsf {PMCP}}_{{\mathcal G}}(\{\overline{P}\},\{\varphi \})$ is $2^{\varOmega (\sqrt{k})}$.

#### Proof

The templates $\overline{P}$ Let *W* be a process template whose state set consists of one state *s*, and whose transitions are $(s,\mathsf {tok}!,s)$ and $(s,\mathsf {tok}?,s)$. The state-labeling of *s* is $\{p\}$. Let $\overline{P} = (W,W)$. For any topology $G=(V,E,T_{1},T_2)$ with $|T_{1}|=1$, $\overline{P}^{G}$ behaves similarly to the graph LTS: the token moves freely between the processes on along the edges of *G*.

We now describe the parameterized topology ${\mathcal G}= \{G_n \mid n > 0\}$. See also Fig. 11. Let $pr_{i}:i\in \mathbb {N}$ be the sequence of prime numbers arranged according to size. Let $t,k^{\prime }\in \mathbb {N}$ be the maximal integers such that

$$\begin{aligned} \begin{array}{lll} k &{}\ge &{} 3k^{\prime }+1\\ k^{\prime }&{}{:=} &{} \displaystyle {\sum _{i \le t}pr_{i}} \end{array} \end{aligned}$$

Let $R=\{(i,j) \mid j\in [pr_i]\}$. For every $n \in \mathbb {N}$, let $R_n = \{(i,j,n) \mid (i,j) \in R\}$. Let $H_0$ be a star $S_{k^{\prime }}$ with $k^{\prime }$ leaves whose center is denoted $start$. Let $H_{n+1}$ be obtained from $H_{n}$ by adding the vertices of $R_{n+1}$ and, for every $(i,j)\in R$, adding an edge between (*i*, *j*, *n*) and $(i,j+1,n+1)$, where $j+1$ is taken modulo $pr_{i}$. Let $G_0=H_0$ and let $G_n$ be obtained from $H_n$ by adding, for every $(i,j)\in R$, an edge between (*i*, *j*, 1) and (*i*, *j*, *n*). Note that $G_0$ is not in ${\mathcal G}$.

There is a $(3k^{\prime }+1)$-expression $\rho (x)$ such that $H_n = \rho ^{n-1}(H_1)$. The colors of the vertices of $H_n$ are as follows:

- the $k^{\prime }$ neighbors of $start$ have distinct colors from $2k^{\prime }+1,\ldots ,3k^{\prime }$;
- the vertices in $R_n$ have distinct colors from $1,\ldots ,k^{\prime }$.
- all other vertices (including $start$) have color $3k^{\prime }+1$;

The $(3k^{\prime }+1)$-expression $\rho (x)$ adds $k^{\prime }$ new vertices with distinct colors from $k^{\prime }+1,\ldots ,2k^{\prime }$, connects them appropriately with the vertices of $R_n$ colored $1,\ldots ,k^{\prime }$, recolors all vertices with colors $1,\ldots ,k^{\prime }$ to color $3k^{\prime }+1$, and recolors every vertex of color $c = k^{\prime }+1,\ldots ,2k^{\prime }$ to $c-k^{\prime }$. The $(3k^{\prime }+1)$-expression $\sigma (x)$ adds an edge between the vertices colored *c* and $c+2k^{\prime }$ for every *c* in $1,\ldots ,k^{\prime }$. We have $G_n = \sigma (\rho ^{n-1}(H_1))$. We get that ${\mathcal G}$ is an iteratively constructible 2-topology with width $3k^{\prime }+1 \le k$.

The crucial property of ${\mathcal G}$ is as follows. Let $n\in \mathbb {N}$. The set

$$\begin{aligned} X_{i}=\left\{ (i,j,\ell )\mid j\in [pr_{i}],\,\ell \in [n]\right\} \end{aligned}$$

induces a single undirected cycle in $G_n$ iff $pr_{i}$ does not divide *n*, for all *i*; $X_{i}$ induces $pr_{i}$ undirected cycles in $G_n$ iff $pr_{i}$ divides *n*. Hence, for every $1<n\le \prod _{i\le t}pr_{i}$, $G_{n}$ contains less than $k^{\prime }$ cycles iff $n \not = \prod _{i\le t} pr_i$. Let $\alpha =\exists x_{1}\ldots x_{k^{\prime }+2}\beta$ where

$$\begin{aligned} \begin{array}{lll} \beta &{} = &{} \displaystyle AG\Bigg (\bigwedge _{1\le a\not =b\le k^{\prime }+1}(\lnot p_{x_{a}}\vee \lnot p_{x_{b}}) \wedge \\ &{}&{} \displaystyle \bigwedge _{2\le a\not =b\le k^{\prime }+1}(\lnot p_{x_{a}}Up_{x_{1}})\vee (\lnot p_{x_{b}}Up_{x_{1}})\Bigg ) \end{array} \end{aligned}$$

So, $\alpha$ says there exists $g_{1},\ldots ,g_{k^{\prime }+1}$ such that (1) $g_{1},\ldots ,g_{k^{\prime }+1}$ are all distinct vertices, which in particular implies that $n\ge 2$, and (2) all paths between $g_{a}$ and $g_{b}$, $a\not =b$, pass through $g_{1}$. Hence, $\overline{P}^{G_{n}}\not \models \alpha$ for any $n < \prod _{i\le t}pr_i$. On the other hand, $\overline{P}^{G_{n}}\models \alpha$ for $n = \prod _{i\le t}pr_i$ with $g_1$ at $start$, and $g_2,\ldots ,g_{k^{\prime }+1}$ on pairwise distinct cycles. Hence $\prod _{i\le t}pr_i$ is the smallest cutoff for ${\textsf {PMCP}}_{{\mathcal G}}(\{\overline{P}\},\{\lnot \alpha \})$.

From well-known properties of primes, $t=\varTheta (\sqrt{k^{\prime }})$, $pr_{t+1}= \theta (t \log t)$ and $\prod _{i\le t}pr_{i}=\varTheta (e^{t})$ (see e.g., [39]). By the maximality of *t* and $k^{\prime }$, $k-(3k^{\prime }+1)< 3pr_{t+1}$, implying that $k=\varTheta (k^{\prime })$. Hence, and $\prod _{i\le t}p_{i}=2^{\varTheta (\sqrt{k})}$. $\square$

Since the parameterized topology in Theorem 28 is $\textsf {MSO}$-definable and has bounded clique-width, we have:

#### Theorem 29

There exists a 2-ary system template $\overline{P}$, and, for every $k\in \mathbb {N}$, an $\textsf {MSO}$-definable 2-topology ${\mathcal G}$ of clique-width at most *k* and a *k*-indexed $\textsf {LTL}\backslash \textsf {X}$ formula $\varphi$ such that the smallest cutoff for ${\textsf {PMCP}}_{{\mathcal G}}(\{\overline{P}\},\{\varphi \})$ is $2^{\varOmega (\sqrt{k})}$.

## Discussion and related work

The applicability of the reduction of the PMCP to finitely many classical model checking problems as a technique for solving the PMCP depends on the communication primitive, the specification language, and the set of topologies of the system. The wide-ranging nature of our work along these axes gives us some insights which may be pertinent to system models different from our own:

**Decidability but no cutoffs.** Theorems 4 and 10 show that, for certain sets of specifications formula, cutoffs do not exist yet the PMCP problem is decidable.

**Cutoffs may not be optimal.** Theorem 15 and Theorem 17 imply that even in cases that cutoffs exist and are computable, they may not yield optimal algorithms for solving the PMCP.

**Table 1:** Complexity of PMCP for different controlled topologies

	PR	DG	TPS
*Ring*			
1-$\textsf {LTL}\backslash \textsf {X}$	Undecidable (Theorem 13)	Undecidable (Theorem 13)	Pspace-complete [16, 28]$^\mathrm{a}$
1-$\textsf {CTL}^*\backslash \textsf {X}$	Undecidable (Theorem 13)	Undecidable (Theorem 13)	Pspace-complete [28]$^\mathrm{b}$
*Homogeneous*			
1-$\textsf {LTL}\backslash \textsf {X}$	ExPACE-complete$^\mathrm{c}$ (Theorem 10)	Pspace-complete (Theorem 19)	Decidable (Corollary 4)
1-$\textsf {CTL}^*\backslash \textsf {X}$	Undecidable (Theorem 2)	Undecidable (Theorem 12)	Decidable (Corollary 4)
*MSO-definable and bounded clique-width*			
1-$\textsf {LTL}\backslash \textsf {X}$	Undecidable [28, 52]	Undecidable (Theorem 13)	Decidable (Theorem 26)
1-$\textsf {CTL}^*\backslash \textsf {X}$	Undecidable ([28, 52], Theorem 2)	Undecidable (Theorem 12)	Decidable (Theorem 26)

${}^\mathrm{a}$ The cited papers show that a cutoff exists and is independent from the input; Sistla and Clarke [50] showed that model checking $\textsf {LTL}\backslash \textsf {X}$ is already Pspace-complete

${}^\mathrm{b}$ Same as for 1-$\textsf {LTL}\backslash \textsf {X}$

${}^\mathrm{c}$ The hardness result holds for controlled clique parameterized topologies

**Table 2:** Complexity of PMCP of 1-indexed formula for different controllerless topologies (the TPS column is missing, since our definition of TPS requires a controlled topology)

	PR	DG
*Ring*		
$\textsf {LTL}\backslash \textsf {X}$	?	?
$\textsf {CTL}^*_{2}\backslash \textsf {X}$	?	?
*Homogeneous (complexity/program complexity)*		
$\textsf {LTL}\backslash \textsf {X}$	Pspace-complete/Ptime (Theorem 11)	Pspace-complete/Ptime (Theorem 19, Corollary 2)
$\textsf {CTL}^*_{2}\backslash \textsf {X}$	Undecidable (Theorem 2)	Undecidable (Theorem 12)
*MSO and bounded clique-width*		
$\textsf {LTL}\backslash \textsf {X}$	?	?
$\textsf {CTL}^*_{2}\backslash \textsf {X}$	Undecidable (Theorem 2)	Undecidable (Theorem 12)

**Table 3:** Regularity for the set of executions on homogeneous topologies. Sizes of NBW are given where appropriate

	PR	DG$^\mathrm{a}$	TPS
*Controlled*			
controller	non $\omega$-regular (Theorem 8)	$O(C^2 \cdot 2^{U})$ (Theorem 18)	$\omega$-regular (Corollary 3)
user	non $\omega$-regular (Theorem 8)	$O(C^2 \cdot 2^{U})$ (Theorem 18)	$\omega$-regular (Corollary 3)
*Controllerless*			
user	*O*(|*U*|) (Theorem 7)	$O(2^U)$ (Theorem 18)	–${}^\mathrm{b}$

$^\mathrm{a}$ Here *C* is the product of sizes of controller templates, *U* is the sum of sizes of user templates

$^\mathrm{b}$ Our definition of TPS requires the presence of a controller, thus this combination does not represent a meaningful question

**Formalisms for topologies are useful.** Many results in Sects. 3 and 5 show that decidability and complexity of PMCP can be extended from concrete examples of sets of topologies such as rings and cliques to infinite classes of topologies given as user-friendly yet powerful formalisms. The formalisms we study may be useful for other system models; for instance, in the context of model-checking multi-agent systems in unknown parameterized environments [4, 5, 47].

In the context of cutoffs, it is worth noting that we considered cutoffs with respect to sets of formulas and process templates. As Theorem 4 shows, there is a parameterized topology ${\mathcal G}$, and a pairwise-rendezvous system template $\overline{P}$, for which no cutoff exists for the set of 1-indexed $\textsf {LTL}\backslash \textsf {X}$ formulas. Note, however, that if the set of formulas ${\mathcal F}$ being considered is finite, then a cutoff always exists. Indeed, given ${\mathcal G}, \overline{P}, \varphi \in {\mathcal F}$, let $G_\varphi$ be a smallest topology *G* for which $\overline{P}^G\not \models \varphi$, and if none exists, then let $G_\varphi$ be a smallest topology in ${\mathcal G}$. Then $\max _{\varphi \in {\mathcal F}} |V_{G_\varphi }|$ is a (minimal) cutoff for ${\textsf {PMCP}}_{{\mathcal G}}(\{\overline{P}\},{\mathcal F})$ in case ${\mathcal F}$ is finite.

Let us underline that the cutoffs we compute are linear in the number of states in the case of disjunctively-guarded systems, and exponential in the case of token-passing systems. We have shown that such cutoffs are already useful for establishing the decidability of the parameterized model checking problem. On the other hand, some experimental results on PMCP from real-world case studies (e.g., [25, 42] or [38, Sec. 6.2]) suggest that templates in such systems usually have tens or hundreds of states, suggesting that small cutoffs are desirable if one is to use them to solve the PMCP in practice. Interestingly, case studies have found that sometimes very small cutoffs do exist. For instance, [42] provide experimental results for checking reachability properties on Boolean programs and Petri nets. Note that both Boolean programs and Petri nets can be modeled by pairwise-rendezvous systems in controlled-clique topologies. Moreover, since there are only finitely many reachability properties, cutoffs are guaranteed to exist. They provide a dynamic approach to detecting cutoffs, i.e., they do a reachability analysis on systems with an increasing number of processes, until a certain stopping criterion is reached, producing a cutoff. To the best of our knowledge, it is an open research question whether a notion of dynamic cutoff exists for doing PMCP of pairwise-rendezvous systems against indexed $\textsf {LTL}\backslash \textsf {X}$ specifications in practice.

As previously discussed, this work draws on and generalizes the work in [36] on pairwise rendezvous on cliques, the work in [24] on disjunctive guards on cliques, and the work in [2, 16, 28] on token-passing systems. There are very few published complexity lower-bounds for PMCP (notable exceptions are [30, 48]), and to the best of our knowledge, our lower bounds on the sizes of cutoffs are the first proven non-trivial lower bounds for these types of systems.

In Tables 1, 3 and 4 we summarized the answers given to the aforementioned problems earlier for *controlled topologies*. In Table 2 we collect answers to the complexity problems for the case of controllerless topologies. Such answers and questions, collected together, give an idea of the several combinations of *process templates*, *synchronization mechanisms*, *process topologies* and *specification language* that have been considered in this and previous works. We hope that analyzing such combinations as a “problem space” as well as the differences among the provided answers, will be helpful to further understanding the role played by every item in such space. Finally, question marks appearing in the tables represent combinations that have not been explored in the research area of parameterized model checking of rendezvous-systems and we hope they might represent good starting points for further research.

**Table 4:** Cutoffs for PMCP on different logics and controlled and controllerless parameterized topologies

	PR	DG	TPS${}^\mathrm{a}$
*Ring*			
1-$\textsf {LTL}\backslash \textsf {X}$	Does not exist (Theorem 13)	Does not exist (Theorem 13)	2 or 3 [16, 28]
1-$\textsf {CTL}^*\backslash \textsf {X}$	Does not exist (Theorem 13)	Does not exist (Theorem 13)	2 or 3 [28]
*Homogeneous*			
1-$\textsf {LTL}\backslash \textsf {X}$	Does not exist (Theorem 5)	$2 + nc + \varSigma _i |U_i|$ (Theorem 14)$^\mathrm{b}$	2 [2]
1-$\textsf {CTL}^*\backslash \textsf {X}$	Does not exist (Theorem 5, Theorem 2)	Does not exist (Theorem 12)	2 [2]
MSO * and bounded clique-width*			
k-$\textsf {LTL}\backslash \textsf {X}$	Does not exist (Theorem 5)	?	$F(\mathcal {G},k,1)$ (Theorem 26) $2^{\varOmega (\sqrt{k})}$ (Theorem 29)
k-$\textsf {CTL}^*\backslash \textsf {X}$	Does not exist (Theorem 2)	Does not exist (Theorem 12)	$F(\mathcal {G},k,d)$ (Theorem 26) $2^{\varOmega (\sqrt{k})}$ (Theorem 29)

${}^\mathrm{a}$ In this column we answer the question whether there are cutoffs which are *computable given parameterized topology* $\mathcal {G}$. $F(\mathcal {G},k,d)$ is some computable function of $\mathcal {G}$, *k*, and *d* defined in the respective theorems, where *d* is the number of nested path quantifiers

${}^\mathrm{b}$ Here *nc* denotes the number of controller templates, $|U_i|$ denotes the size of the *i*-th user template

In this context, it is worth noting that all the upper-bounds presented in this paper concerning 1-indexed $\textsf {LTL}\backslash \textsf {X}$ can be easily extended to the existential or universal fragments of *k*-indexed $\textsf {LTL}\backslash \textsf {X}$ (for $k \in \mathbb {N}$), i.e., to the case of many process quantifiers of the same type (all existential or all universal). Furthermore, this is also the case if one allows enhanced versions of these quantifiers that specify that the processes quantified are different, and/or are neighbours (or not) in the topology (see [2] for a definition of these enhanced quantifiers). This allows one, for example, to express mutual-exclusion: $\forall i \ne j.\; \textsf {G}(\lnot \text{(critical }, i) \vee \lnot \text{(critical }, j))$. For the case of full *k*-indexed $\textsf {LTL}\backslash \textsf {X}$  where alternation of universal and existential quantifiers is allowed, many of the corresponding upper bounds are still unknown, and represent another direction for future work.

We now briefly describe what needs to be done to get this extension. All the upper bounds concerning 1-indexed $\textsf {LTL}\backslash \textsf {X}$ were stated with respect to homogeneous parameterized topologies.^13^ Lemma 3 shows that, for 1-indexed $\textsf {LTL}\backslash \textsf {X}$, such systems can be simulated by cliques. However, looking at the proof of the lemma, it is not hard to see that this simulation actually works irrespective of the specification logic. Indeed, in the controllerless case we actually get that the set of runs of the cliques is exactly equal to the set of runs of the homogeneous topologies; and in the controlled case this is also true except for a slight technical mismatch between the structure of global configurations in these two types of systems—due to the fact that the single controller of a controlled clique simulates (using a product process template) all the controllers specified by the homogeneous parameterised topology skeleton. However, this technical mismatch is syntactic in nature, and is easily overcome by mapping each coordinate in the state of the unique clique controller to the corresponding controller vertex in the homogeneous topology. Also note that runs of a given topology *G* in the homogeneous parameterized topology are simulated by runs of a clique topology $G^{\prime }$ of the same size or smaller; conversely, all runs of a simulating clique topology $G^{\prime }$ correspond to runs of topologies in the parameterized homogeneous topology that are larger by at most a constant factor (namely, the number of controllers in the skeleton of the homogeneous topology minus 1).

Armed with the above observations, extending our upper-bounds from 1-indexed $\textsf {LTL}\backslash \textsf {X}$ to the universal and existential fragments of *k*-indexed $\textsf {LTL}\backslash \textsf {X}$ now requires the following. First, we can easily extend the construction in the proof of Lemma 3 to have the controller of the clique simulate not only the controllers of the homogeneous topology, but also any other *k* nodes of *k* different types. Combining this with the observation made in Lemma 2 that, due to symmetry, in a homogeneous system the executions of all processes of a given type are exactly the same, we reach the following conclusion: we can replace reasoning about properties of the set of all runs of a homogeneous parameterised system projected onto processes of *k* different types with reasoning about the 1-executions of the unique controller of a parameterized clique topology, projected onto the relevant *k* simulated nodes of interest. Moreover, this reduction incurs only a constant blowup (assuming *k* and the communication alphabet are fixed). Observe that in case we started with a homogeneous parameterized topology with no controllers (and *k* types of interest), we do not have to simulate it with a controlled clique-topology. Instead, we can simulate it with a clique topology with two types: one type that is the disjoint union of all the process templates (as in the basic construction in Lemma 3), and one which is the product of the *k* process templates of interest (similar to the controller case—but not designated as a controller, i.e., allowing one to have many nodes of this product type). Thus, in all cases we can reduce questions about universal and existential *k*-indexed $\textsf {LTL}\backslash \textsf {X}$ formulas with respect to a homogeneous parameterized topology to a question about a 1-indexed $\textsf {LTL}\backslash \textsf {X}$ formula with respect to a clique topology of the same type (controlled or uncontrolled), with a constant blowup.

As a final remark, observe that when the given existential *k*-indexed $\textsf {LTL}\backslash \textsf {X}$ formula does not specify that two quantified vertices *x*, *y* should be different, we can replace it with the disjunction (conjunction for a universal formula) of two formulas: one specifying that $x \ne y$, and one with one quantifier less and replacing every occurrence of *y* with *x*. For a fixed *k*, performing this for every possible pair of variables, incurs a constant blowup.

## Footnotes

1 Cutoffs are also useful for parameterized synthesis, see e.g., [13, 40, 43].

2 Actually, the set of executions is defined to be the destuttering of this set of projections, as explained in Sect. 2.6.

3 In the body of the paper, sets of topologies are called “parameterized topologies”.

4 A controller refers to a process-template that is not duplicated, i.e., it occurs exactly once.

5 We remark that allowing processes to send in certain directions, e.g., send left and send right in a bi-directional ring, quickly makes the PMCP undecidable [2].

6 In this paper, the sets in a partition may be empty.

7 The definition we give in this paper for indexed temporal logic uses a longhand notation. Thus, in a system with a single process template, instead of writing $\forall i,j$ we write $\forall i:type(i)=1 \ \forall j:type(j)=1$. Also, for the sake of simplicity, we only consider the basic universal and existential quantifiers, ignoring quantifiers of the form $\forall i \ne j$ that require that the process assigned to *i* is different than the one assigned to *j* (see for example [2] for a definition that allows such enhanced quantifiers). All the results in this paper also hold for the case where the enhanced quantifiers as defined in [2] are used, with the natural changes applied to the proofs.

8 These multiple initial states can be removed by introducing a dummy initial state $\iota ^{\prime }_1$; in this case the statement of the Lemma needs to be slightly amended to remove the first state of each execution in $\textsc {1-exec}_{{\mathcal G}^\prime }{(\overline{P}^{\prime })}$.

9 Note that it is very common to assume that the atomic propositions are equal to the states.

10 Note that not all such products are legal paths in *A*, since *A* contains a transition from (*p*, *q*) to $(p^{\prime },q^{\prime })$ only if $A_{\lnot \phi ^{\prime }}$ has a transition from *p* to $p^{\prime }$ while reading the atomic propositions that are true in *q*.

11 To see this, note that $K^{\prime }(C,U)$ is the destuttering of an automaton with $O(|{T}|)$ many states and input alphabet of size |*C*|, and apply Lemma 1.

12
$\varphi$ is also a $\textsf {CTL}\backslash \textsf {X}$ formula.

13 With the exception of Theorem 1. However, the construction in this Theorem can be easily extended without requiring any new ideas.

## References

[1] Abdulla, P.A., Atig, M.F., Rezine, O.: Verification of directed acyclic ad hoc networks. In: Beyer, D., Boreale, M. (eds) Formal Techniques for Distributed Systems: Joint IFIP WG 6.1 International Conference, FMOODS/FORTE 2013, Held as Part of the 8th International Federated Conference on Distributed Computing Techniques, DisCoTec 2013, Florence, Italy, 3-5 June 2013, Proceedings, pp. 193–208, Springer, Berlin, Heidelberg (2013). doi:10.1007/978-3-642-38592-6_14

[2] Aminof, B., Jacobs, S., Khalimov, A., Rubin, S.: Parameterized model checking of token-passing systems. In: McMillan, K.L., Rival, X. (eds.) Verification, Model Checking, and Abstract Interpretation—15th International Conference, VMCAI 2014, San Diego, CA, USA, January 19–21, 2014, Proceedings, Volume 8318 of Lecture Notes in Computer Science, pp. 262–281. Springer (2014)

[3] Aminof, B., Kotek, T., Rubin, S., Spegni, F., Veith, H.: Parameterized model checking of rendezvous systems. In: Baldan, P., Gorla, D. (eds.) CONCUR 2014—Concurrency Theory—25th International Conference, CONCUR 2014, Rome, Italy, September 2–5, 2014. Proceedings, Volume 8704 of Lecture Notes in Computer Science, pp. 109–124. Springer (2014)

[4] Aminof, B., Murano, A., Rubin, S., Zuleger, F.: Verification of asynchronous mobile-robots in partially-known environments. In: PRIMA 2015: Principles and Practice of Multi-agent Systems—18th International Conference, Bertinoro, Italy, October 26–30, 2015, Proceedings, pp. 185–200 (2015)

[5] Aminof, B., Murano, A., Rubin, S., Zuleger, F.: Automatic verification of multi-agent systems in parameterised grid-environments. In: Proceedings of the 2016 International Conference on Autonomous Agents & Multiagent Systems, Singapore, May 9–13, 2016, pp. 1190–1199 (2016)

[6] Aminof, B., Rubin, S.: Model checking parameterised multi-token systems via the composition method. In: Olivetti, N., Tiwari, A. (eds.) Automated Reasoning—8th International Joint Conference, IJCAR 2016, Coimbra, Portugal, June 27–July 2, 2016, Proceedings, Volume 9706 of Lecture Notes in Computer Science, pp. 499–515. Springer (2016)

[7] Aminof, B., Rubin, S., Zuleger, F.: On the expressive power of communication primitives in parameterised systems. In: Davis, M., Fehnker, A., McIver, A., Voronkov, A. (eds.) Logic for Programming, Artificial Intelligence, and Reasoning—20th International Conference, LPAR-20 2015, Suva, Fiji, November 24–28, 2015, Proceedings, Volume 9450 of Lecture Notes in Computer Science, pp. 313–328. Springer (2015)

[8] Aminof, B., Rubin, S., Zuleger, F., Spegni, F.: Liveness of parameterized timed networks. In: Halldórsson, M.M., Iwama, K., Kobayashi, N., Speckmann, B. (eds.) Automata, Languages, and Programming—42nd International Colloquium, ICALP 2015, Kyoto, Japan, July 6–10, 2015, Proceedings, Part II, Volume 9135 of Lecture Notes in Computer Science, pp. 375–387. Springer (2015)

[9] Angluin D, Aspnes J, Eisenstat D, Ruppert E (2007). The computational power of population protocols. Distrib. Comput..

[10] Außerlechner, S., Jacobs, S., Khalimov, A.: Tight cutoffs for guarded protocols with fairness. In: Jobstmann, B., Leino, K.R.M. (eds.) Verification, Model Checking, and Abstract Interpretation—17th International Conference, VMCAI 2016, St. Petersburg, FL, USA, January 17–19, 2016. Proceedings, Volume 9583 of Lecture Notes in Computer Science, pp. 476–494. Springer (2016)

[11] Baier C, Katoen J-P (2008). Principles of Model Checking.

[12] Ball, T., Bounimova, E., Cook, B., Levin, V., Lichtenberg, J., McGarvey, C., Ondrusek, B., Rajamani, S.K., Ustuner, A.: Thorough static analysis of device drivers. In: Berbers, Y., Zwaenepoel, W. (eds.) Proceedings of the 2006 EuroSys Conference, Leuven, Belgium, April 18–21, 2006, pp. 73–85. ACM (2006)

[13] Bloem, R., Jacobs, S., Khalimov, A.: Parameterized synthesis case study: AMBA AHB. In: Proceedings 3rd Workshop on Synthesis, SYNT 2014, Volume 157 of EPTCS (2014)

[14] Bloem R, Jacobs S, Khalimov A, Konnov I, Rubin S, Veith H, Widder J (2015). Decidability of parameterized verification. Synth Lect. Distrib. Comput. Theory.

[15] Browne MC, Clarke EM, Grumberg O (1989). Reasoning about networks with many identical finite state processes. Inf. Comput..

[16] Clarke, E.M., Talupur, M., Touili, T., Veith, H.: Verification by network decomposition. In: Gardner, P., Yoshida, N. (eds.) CONCUR 2004—Concurrency Theory, 15th International Conference, London, UK, August 31–September 3, 2004, Proceedings, Volume 3170 of Lecture Notes in Computer Science, pp. 276–291. Springer (2004)

[17] Courcelle B, Engelfriet J (2012). Graph Structure and Monadic Second-Order Logic—A Language-Theoretic Approach, Volume 138 of Encyclopedia of Mathematics and its Applications.

[18] Courcelle B, Makowsky JA, Rotics U (2000). Linear time solvable optimization problems on graphs of bounded clique-width. Theory Comput. Syst..

[19] Courcelle B, Olariu S (2000). Upper bounds to the clique width of graphs. Discrete Appl. Math..

[20] Delzanno, G., Raskin, J.-F., Van Begin, L.: Towards the automated verification of multithreaded java programs. In: Proceedings of the 8th International Conference on Tools and Algorithms for the Construction and Analysis of Systems, TACAS ’02. Springer (2002)

[21] Delzanno, G., Sangnier, A., Traverso, R.: Parameterized verification of broadcast networks of register automata. In: Proceedings of the 7th International Workshop on Reachability Problems (RP’13), Volume 8169 of Lecture Notes in Computer Science. Springer (2013)

[22] Delzanno, G., Traverso, R.: Decidability and complexity results for verification of asynchronous broadcast networks. In: Dediu, A-H., Martín-Vide, C., Truthe, B. (eds.) Language and Automata Theory and Applications: 7th International Conference, LATA 2013, Bilbao, Spain, 2-5 April 2013, Proceedings, pp. 239–249, Springer, Berlin, Heidelberg (2013). doi:10.1007/978-3-642-37064-9_22

[23] Ebbinghaus H-D, Flum J (2005). Finite Model Theory.

[24] Emerson, E.A., Kahlon, V.: Reducing model checking of the many to the few. In: CADE. Springer (2000)

[25] Emerson, E.A., Kahlon, V.: Exact and efficient verification of parameterized cache coherence protocols. In: Geist, D., Tronci, E. (eds.) Correct Hardware Design and Verification Methods, 12th IFIP WG 10.5 Advanced Research Working Conference, CHARME 2003, L’Aquila, Italy, October 21–24, 2003, Proceedings, Volume 2860 of Lecture Notes in Computer Science, pp. 247–262. Springer (2003)

[26] Emerson, E. A., Kahlon, V.: Parameterized model checking of ring-based message passing systems. In: Marcinkowski, J., Tarlecki, A. (eds.) Computer Science Logic, 18th International Workshop, CSL 2004, 13th Annual Conference of the EACSL, Karpacz, Poland, September 20–24, 2004, Proceedings, Volume 3210 of Lecture Notes in Computer Science, pp. 325–339. Springer (2004)

[27] Emerson, E.A., Namjoshi, K.S.: On model checking for non-deterministic infinite-state systems. In: Thirteenth Annual IEEE Symposium on Logic in Computer Science, Indianapolis, Indiana, USA, June 21–24, 1998, pp. 70–80. IEEE Computer Society (1998)

[28] Emerson EA, Namjoshi KS (2003). On reasoning about rings. Int. J. Found. Comput. Sci..

[29] Esparza, J.: Decidability and complexity of petri net problems—an introduction. In: Reisig, W., Rozenberg, G. (eds.) Lectures on Petri Nets I: Basic Models: Advances in Petri Nets, pp. 374–428, Springer, Berlin, Heidelberg. doi:10.1007/3-540-65306-6_20

[30] Esparza, J.: Keeping a crowd safe: on the complexity of parameterized verification (invited talk). In: Mayr, E.W., Portier, N. (eds.) 31st International Symposium on Theoretical Aspects of Computer Science (STACS 2014), STACS 2014, March 5–8, 2014, Lyon, France, Volume 25 of LIPIcs, pp. 1–10. Schloss Dagstuhl - Leibniz-Zentrum fuer Informatik (2014)

[31] Esparza, J., Finkel, A., Mayr, R.: On the verification of broadcast protocols. In: 14th Annual IEEE Symposium on Logic in Computer Science, Trento, Italy, July 2–5, 1999, pp. 352–359. IEEE Computer Society (1999)

[32] Esparza, J., Ganty, P., Leroux, J., Majumdar, R.: Verification of population protocols. In: 26th International Conference on Concurrency Theory, CONCUR 2015, Volume 42 of LIPIcs. Schloss Dagstuhl - Leibniz-Zentrum fuer Informatik (2015)

[33] Fischer, E., Makowsky, J.A.: The Specker-Blatter theorem revisited. In: Computing and Combinatorics, 9th Annual International Conference, COCOON 2003, Proceedings (2003)

[34] Fischer, E., Makowsky, J.A.: Linear recurrence relations for graph polynomials. In: In: Avron, A., Dershowitz, N., Rabinovich, A. (eds.) Pillars of Computer Science: Essays Dedicated to Boris (Boaz) Trakhtenbrot on the Occasion of His 85th Birthday, pp. 266–279, Springer, Berlin, Heidelberg (2008). doi:10.1007/978-3-540-78127-1_15

[35] Flum J, Grohe M (2006). Parameterized Complexity Theory (Texts in Theoretical Computer Science. An EATCS Series).

[36] German SM, Sistla AP (1992). Reasoning about systems with many processes. J. ACM.

[37] Glaister I, Shallit J (1996). A lower bound technique for the size of nondeterministic finite automata. Inf. Process. Lett..

[38] Gmeiner, A., Konnov, I., Schmid, U., Veith, H., Widder, J.: Tutorial on parameterized model checking of fault-tolerant distributed algorithms. In: Formal Methods for Executable Software Models—14th International School on Formal Methods for the Design of Computer, Communication, and Software Systems, SFM 2014, Bertinoro, Italy, June 16–20, 2014, Advanced Lectures, pp. 122–171 (2014)

[39] Graham, R.L., Knuth, D.E., Patashnik, O.: Concrete Mathematics: A Foundation for Computer Science, 2nd edn. Addison-Wesley, Reading (1994)

[40] Jacobs, S., Bloem, R.: Parameterized synthesis. Log. Methods Comput. Sci. **10**(1), 362–376 (2014)

[41] John, A., Konnov, I., Schmid, U., Veith, H., Widder, J.: Counter attack on byzantine generals: parameterized model checking of fault-tolerant distributed algorithms. CoRR, abs/1210.3846 (2012)

[42] Kaiser, A., Kroening, D., Wahl, T.: Dynamic cutoff detection in parameterized concurrent programs. In: Touili, T., Cook, B., Jackson, P.B. (eds.) Computer Aided Verification, 22nd International Conference, CAV 2010, Edinburgh, UK, July 15–19, 2010. Proceedings, Volume 6174 of Lecture Notes in Computer Science, pp. 645–659. Springer (2010)

[43] Khalimov, A., Jacobs, S., Bloem, R.: PARTY parameterized synthesis of token rings. In: Computer Aided Verification—25th International Conference, CAV 2013, Proceedings, Volume 8044 of Lecture Notes in Computer Science. Springer (2013)

[44] Libkin, L.: Elements of Finite Model Theory. In: Brauer, W., Rozenburg, G., Salomaa, A. (eds.) Texts in Theoretical Computer Science. An EATCS Series. Springer Berlin, Heidelberg (2004)

[45] Makowsky JA (2004). Algorithmic uses of the Feferman–Vaught theorem. Ann. Pure Appl. Log..

[46] Minsky ML (1967). Computation: Finite and Infinite Machines.

[47] Rubin, S.: Parameterised verification of autonomous mobile-agents in static but unknown environments. In: Proceedings of the 2015 International Conference on Autonomous Agents and Multiagent Systems, AAMAS 2015, pp. 199–208 (2015)

[48] Schmitz, S., Schnoebelen, P.: The power of well-structured systems. In: D’Argenio, P.R., Melgratti, H.C. (eds.) CONCUR 2013—Concurrency Theory—24th International Conference, CONCUR 2013, Buenos Aires, Argentina, August 27-30, 2013. Proceedings, Volume 8052 of Lecture Notes in Computer Science, pp. 5–24. Springer (2013)

[49] Shamir S, Kupferman O, Shamir E (2003). Branching-depth hierarchies. Electron. Notes Theor. Comput. Sci..

[50] Sistla AP, Clarke EM (1985). The complexity of propositional linear temporal logics. J. ACM (JACM).

[51] Spalazzi, L., Spegni, F.: Parameterized model-checking of timed systems with conjunctive guards. In: Verified Software: Theories, Tools and Experiments—6th International Conference, VSTTE 2014, Volume 8471 of Lecture Notes in Computer Science. Springer (2014)

[52] Suzuki I (1988). Proving properties of a ring of finite-state machines. Inf. Process. Lett..

[53] Vardi M, Wolper P (1986). Automata-theoretic techniques for modal logics of programs. J. Comput. Syst. Sci..

